# Supplementary material for: microRNA-122 amplifies hepatitis C virus translation by shaping the structure of the internal ribosomal entry site
Source: Nat Commun. 2018 Jul 4;9:2613. doi: 10.1038/s41467-018-05053-3 (PMC6031695; doi:10.1038/s41467-018-05053-3)
Supplement: Supplementary file 1 — Supplementary Data 1 [file 41467_2018_5053_MOESM1_ESM.docx]

**­­**

**Supplementary Data 1 - Sequences for structural phylogeny**

1. JF735116.1/1-119 ACCUGCCCCUAA-UA----GGGG-CGACACUCCACCAUGAAUCACUCCCCUGUGAGGAACUACUGUCUUCACGCAGAAAGCGUCUAGCCAUGGCGUUAGUAUGAGUGUCGUACAGCCUCCAGGCC
2. JF735115.1/1-119 ACCUGCCCCUAA-UA----GGGG-CGACACUCCACCAUGAAUCACUCCCCUGUGAGGAACUACUGUCUUCACGCAGAAAGCGUCUAGCCAUGGCGUUAGUAUGAGUGUCGUACAGCCUCCAGGCC
3. KM504113.1/1-118 GCCAGCCCCCUA-AU----GGGG-CGACACUCCACCAUA-AUCACUCCCCUGUGAGGAACUACUGUCUUCACGCAGAAAGCGUCUAGCCAUGGCGUUAGUAUGAGUGUCGUGCAGCCUCCAGGAC
4. AB031663.1/2-120 GCCCGCCCCUAA-AU----GGGG-CGACACUCCGCCAUGAAUCACUCCCCUGUGAGGAACUACUGUCUUCACGCAGAAAGCGUCUAGCCAUGGCGUUAGUAUGAGUGUCGUACAGCCUCCAGGCC
5. JF735112.1/1-119 ACCUGCCCCCUA-UA----GGGG-CGACACUCCGCCAUGAAUCACUCCCCUGUGAGGAACUACUGUCUUCACGCAGAAAGCGUCUAGCCAUGGCGUUAGUAUGAGUGUCGUACAGCCUCCAGGCC
6. KY620329.1/2-120 ACCCGCCCCUAA-AU----GGGG-CGACACUCCGCCAUGAAUCACUCCCCUGUGAGGAACUACUGUCUUCACGCAGAAAGCGUCUAGCCAUGGCGUUAGUAUGAGUGUCGUACAGCCUCCAGGCC
7. AJ132997.1/1-119 GCCAGCCCCUGU-UG----GGGG-CGACACUCCACCAUAGAUCACUCCCCUGUGAGGAACUACUGUCUUCACGCAGAAAGCGUCUAGCCAUGGCGUUAGUAUGAGUGUCGUGCAGCCUCCAGGAC
8. AJ132996.1/1-119 GCCAGCCCCUGU-UG----GGGG-CGACACUCCACCAUAGAUCACUCCCCUGUGAGGAACUACUGUCUUCACGCAGAAAGCGUCUAGCCAUGGCGUUAGUAUGAGUGUCGUGCAGCCUCCAGGAC
9. KJ925146.1/1-118 ACCCGCCCCUUA-UU----GGGG-CGACACUCCACCAUG-AUCACUCCCCUGUGAGGAACUACUGUCUUCACGCAGAAAGCGUCUAGCCAUGGCGUUAGUAUGAGUGUCGUACAGCCUCCAGGAC
10. KJ439781.1/1-120 GCCAGCCCCCUG-AU----GGGGgCGACACUCCACCAUGAAUCACUCCCCUGUGAGGAACUACUGUCUUCACGCAGAAAGCGUCUAGCCAUGGCGUUAGUAUGAGUGUCGUACAGCCUCCAGGAC
11. KC248193.1/1-120 GCCAGCCCCCUG-AU----GGGGgCGACACUCCACCAUGAAUCACUCCCCUGUGAGGAACUACUGUCUUCACGCAGAAAGCGUCUAGCCAUGGCGUUAGUAUGAGUGUCGUACAGCCUCCAGGAC
12. D50409.1/1-119 ACCCGCCCCUAA-AA----GGGG-CGACACUCCGCCAUGAAUCACUCCCCUGUGAGGAACUACUGUCUUCACGCAGAAAGCGUCUAGCCAUGGCGUUAGUAUGAGUGUCGUACAGCCUCCAGGCC
13. KM587621.1/1-119 ACCCGCCCCUAA-AA----GGGG-CGACACUCCGCCAUGAAUCACUCCCCUGUGAGGAACUACUGUCUUCACGCAGAAAGCGUCUAGCCAUGGCGUUAGUAUGAGUGUCGUACAGCCUCCAGGCC
14. KM587620.1/1-119 ACCCGCCCCUAA-AA----GGGG-CGACACUCCGCCAUGAAUCACUCCCCUGUGAGGAACUACUGUCUUCACGCAGAAAGCGUCUAGCCAUGGCGUUAGUAUGAGUGUCGUACAGCCUCCAGGCC
15. JF735120.1/1-119 ACCUGCCCCUAA-UA----GGGG-CGACACUCCGCCAUGAAUCACUCCCCUGUGAGGAACUACUGUCUUCACGCAGAAAGCGUCUAGCCAUGGCGUUAGUAUGAGUGUCGUACAGCCUCCAGGCC
16. JF735119.1/1-119 ACCUGCCCCUAA-UA----GGGG-CGACACUCCGCCAUGAAUCACUCCCCUGUGAGGAACUACUGUCUUCACGCAGAAAGCGUCUAGCCAUGGCGUUAGUAUGAGUGUCGUACAGCCUCCAGGCC
17. JF735118.1/1-119 ACCUGCCCCUAA-UA----GGGG-CGACACUCCGCCAUGAAUCACUCCCCUGUGAGGAACUACUGUCUUCACGCAGAAAGCGUCUAGCCAUGGCGUUAGUAUGAGUGUCGUACAGCCUCCAGGCC
18. JF735111.1/1-119 ACCUGCCCCUAA-UA----GGGG-CGACACUCCGCCAUGAAUCACUCCCCUGUGAGGAACUACUGUCUUCACGCAGAAAGCGUCUAGCCAUGGCGUUAGUAUGAGUGUCGUACAGCCUCCAGGCC
19. NC_009823.1/1-119 ACCCGCCCCUAA-UA----GGGG-CGACACUCCGCCAUGAAUCACUCCCCUGUGAGGAACUACUGUCUUCACGCAGAAAGCGUCUAGCCAUGGCGUUAGUAUGAGUGUCGUACAGCCUCCAGGCC
20. KF676352.1/1-119 ACCCGCCCCUAA-UA----GGGG-CGACACUCCGCCAUGAAUCACUCCCCUGUGAGGAACUACUGUCUUCACGCAGAAAGCGUCUAGCCAUGGCGUUAGUAUGAGUGUCGUACAGCCUCCAGGCC
21. KF676351.1/1-119 ACCCGCCCCUAA-UA----GGGG-CGACACUCCGCCAUGAAUCACUCCCCUGUGAGGAACUACUGUCUUCACGCAGAAAGCGUCUAGCCAUGGCGUUAGUAUGAGUGUCGUACAGCCUCCAGGCC
22. JF343782.1/1-119 ACCCGCCCCUAA-UA----GGGG-CGACACUCCGCCAUGAAUCACUCCCCUGUGAGGAACUACUGUCUUCACGCAGAAAGCGUCUAGCCAUGGCGUUAGUAUGAGUGUCGUACAGCCUCCAGGCC
23. AF169005.1/1-119 ACCCGCCCCUAA-UA----GGGG-CGACACUCCGCCAUGAAUCACUCCCCUGUGAGGAACUACUGUCUUCACGCAGAAAGCGUCUAGCCAUGGCGUUAGUAUGAGUGUCGUACAGCCUCCAGGCC
24. AF169002.1/1-119 ACCCGCCCCUAA-UA----GGGG-CGACACUCCGCCAUGAAUCACUCCCCUGUGAGGAACUACUGUCUUCACGCAGAAAGCGUCUAGCCAUGGCGUUAGUAUGAGUGUCGUACAGCCUCCAGGCC
25. AF177036.1/1-119 ACCCGCCCCUAA-UA----GGGG-CGACACUCCGCCAUGAAUCACUCCCCUGUGAGGAACUACUGUCUUCACGCAGAAAGCGUCUAGCCAUGGCGUUAGUAUGAGUGUCGUACAGCCUCCAGGCC
26. AB690461.1/1-119 ACCCGCCCCUAA-UA----GGGG-CGACACUCCGCCAUGAAUCACUCCCCUGUGAGGAACUACUGUCUUCACGCAGAAAGCGUCUAGCCAUGGCGUUAGUAUGAGUGUCGUACAGCCUCCAGGCC
27. KJ439779.1/1-120 GCCAGCCCCCUG-AU----GGGGgCGACACUCCACCAUAAAUCACUCCCCUGUGAGGAACUACUGUCUUCACGCAGAAAGCGUCUAGCCAUGGCGUUAGUAUGAGUGUCGUGCAGCCUCCAGGAC
28. KF268446.1/1-119 ACCUGCCCCUAA-UA----GGGG-CGACACUCCGCCAUGAAUCACUCCCCUGUGAGGAACUACUGUCUUCACGCAGAAAGCGCCUAGCCAUGGCGUUAGUAUGAGUGUCGUACAGCCUCCAGGCC
29. KF700370.1/1-119 ACCUGCCCCUAA-UA----GGGG-CGACACUCCGCCAUGAAUCACUCCCCUGUGAGGAACUACUGUCUUCACGCAGAAAGCGCCUAGCCAUGGCGUUAGUAUGAGUGUCGUACAGCCUCCAGGCC
30. KF693783.1/1-119 ACCUGCCCCUAA-UA----GGGG-CGACACUCCGCCAUGAAUCACUCCCCUGUGAGGAACUACUGUCUUCACGCAGAAAGCGCCUAGCCAUGGCGUUAGUAUGAGUGUCGUACAGCCUCCAGGCC
31. KF693782.1/1-119 ACCUGCCCCUAA-UA----GGGG-CGACACUCCGCCAUGAAUCACUCCCCUGUGAGGAACUACUGUCUUCACGCAGAAAGCGCCUAGCCAUGGCGUUAGUAUGAGUGUCGUACAGCCUCCAGGCC
32. KF693781.1/1-119 ACCUGCCCCUAA-UA----GGGG-CGACACUCCGCCAUGAAUCACUCCCCUGUGAGGAACUACUGUCUUCACGCAGAAAGCGCCUAGCCAUGGCGUUAGUAUGAGUGUCGUACAGCCUCCAGGCC
33. KF693780.1/1-119 ACCUGCCCCUAA-UA----GGGG-CGACACUCCGCCAUGAAUCACUCCCCUGUGAGGAACUACUGUCUUCACGCAGAAAGCGCCUAGCCAUGGCGUUAGUAUGAGUGUCGUACAGCCUCCAGGCC
34. KF693779.1/1-119 ACCUGCCCCUAA-UA----GGGG-CGACACUCCGCCAUGAAUCACUCCCCUGUGAGGAACUACUGUCUUCACGCAGAAAGCGCCUAGCCAUGGCGUUAGUAUGAGUGUCGUACAGCCUCCAGGCC
35. KF693778.1/1-119 ACCUGCCCCUAA-UA----GGGG-CGACACUCCGCCAUGAAUCACUCCCCUGUGAGGAACUACUGUCUUCACGCAGAAAGCGCCUAGCCAUGGCGUUAGUAUGAGUGUCGUACAGCCUCCAGGCC
36. KF693777.1/1-119 ACCUGCCCCUAA-UA----GGGG-CGACACUCCGCCAUGAAUCACUCCCCUGUGAGGAACUACUGUCUUCACGCAGAAAGCGCCUAGCCAUGGCGUUAGUAUGAGUGUCGUACAGCCUCCAGGCC
37. KF693776.1/1-119 ACCUGCCCCUAA-UA----GGGG-CGACACUCCGCCAUGAAUCACUCCCCUGUGAGGAACUACUGUCUUCACGCAGAAAGCGCCUAGCCAUGGCGUUAGUAUGAGUGUCGUACAGCCUCCAGGCC
38. KC967479.1/1-119 ACCUGCCCCUAA-UA----GGGG-CGACACUCCGCCAUGAAUCACUCCCCUGUGAGGAACUACUGUCUUCACGCAGAAAGCGCCUAGCCAUGGCGUUAGUAUGAGUGUCGUACAGCCUCCAGGCC
39. KC967478.1/1-119 ACCUGCCCCUAA-UA----GGGG-CGACACUCCGCCAUGAAUCACUCCCCUGUGAGGAACUACUGUCUUCACGCAGAAAGCGCCUAGCCAUGGCGUUAGUAUGAGUGUCGUACAGCCUCCAGGCC
40. KC967477.1/1-119 ACCUGCCCCUAA-UA----GGGG-CGACACUCCGCCAUGAAUCACUCCCCUGUGAGGAACUACUGUCUUCACGCAGAAAGCGCCUAGCCAUGGCGUUAGUAUGAGUGUCGUACAGCCUCCAGGCC
41. KC967476.1/1-119 ACCUGCCCCUAA-UA----GGGG-CGACACUCCGCCAUGAAUCACUCCCCUGUGAGGAACUACUGUCUUCACGCAGAAAGCGCCUAGCCAUGGCGUUAGUAUGAGUGUCGUACAGCCUCCAGGCC
42. JX826592.1/1-119 ACCUGCCCCUAA-UA----GGGG-CGACACUCCGCCAUGAAUCACUCCCCUGUGAGGAACUACUGUCUUCACGCAGAAAGCGCCUAGCCAUGGCGUUAGUAUGAGUGUCGUACAGCCUCCAGGCC
43. JX014307.1/1-119 ACCUGCCCCUAA-UA----GGGG-CGACACUCCGCCAUGAAUCACUCCCCUGUGAGGAACUACUGUCUUCACGCAGAAAGCGCCUAGCCAUGGCGUUAGUAUGAGUGUCGUACAGCCUCCAGGCC
44. JN180460.1/1-119 ACCUGCCCCUAA-UA----GGGG-CGACACUCCGCCAUGAAUCACUCCCCUGUGAGGAACUACUGUCUUCACGCAGAAAGCGCCUAGCCAUGGCGUUAGUAUGAGUGUCGUACAGCCUCCAGGCC
45. JN180459.1/1-119 ACCUGCCCCUAA-UA----GGGG-CGACACUCCGCCAUGAAUCACUCCCCUGUGAGGAACUACUGUCUUCACGCAGAAAGCGCCUAGCCAUGGCGUUAGUAUGAGUGUCGUACAGCCUCCAGGCC
46. JN180458.1/1-119 ACCUGCCCCUAA-UA----GGGG-CGACACUCCGCCAUGAAUCACUCCCCUGUGAGGAACUACUGUCUUCACGCAGAAAGCGCCUAGCCAUGGCGUUAGUAUGAGUGUCGUACAGCCUCCAGGCC
47. JN180457.1/1-119 ACCUGCCCCUAA-UA----GGGG-CGACACUCCGCCAUGAAUCACUCCCCUGUGAGGAACUACUGUCUUCACGCAGAAAGCGCCUAGCCAUGGCGUUAGUAUGAGUGUCGUACAGCCUCCAGGCC
48. JN180456.1/1-119 ACCUGCCCCUAA-UA----GGGG-CGACACUCCGCCAUGAAUCACUCCCCUGUGAGGAACUACUGUCUUCACGCAGAAAGCGCCUAGCCAUGGCGUUAGUAUGAGUGUCGUACAGCCUCCAGGCC
49. JN180455.1/1-119 ACCUGCCCCUAA-UA----GGGG-CGACACUCCGCCAUGAAUCACUCCCCUGUGAGGAACUACUGUCUUCACGCAGAAAGCGCCUAGCCAUGGCGUUAGUAUGAGUGUCGUACAGCCUCCAGGCC
50. JN180454.1/1-119 ACCUGCCCCUAA-UA----GGGG-CGACACUCCGCCAUGAAUCACUCCCCUGUGAGGAACUACUGUCUUCACGCAGAAAGCGCCUAGCCAUGGCGUUAGUAUGAGUGUCGUACAGCCUCCAGGCC
51. JN180453.1/1-119 ACCUGCCCCUAA-UA----GGGG-CGACACUCCGCCAUGAAUCACUCCCCUGUGAGGAACUACUGUCUUCACGCAGAAAGCGCCUAGCCAUGGCGUUAGUAUGAGUGUCGUACAGCCUCCAGGCC
52. JN180452.1/1-119 ACCUGCCCCUAA-UA----GGGG-CGACACUCCGCCAUGAAUCACUCCCCUGUGAGGAACUACUGUCUUCACGCAGAAAGCGCCUAGCCAUGGCGUUAGUAUGAGUGUCGUACAGCCUCCAGGCC
53. HQ852469.1/1-119 ACCUGCCCCUAA-UA----GGGG-CGACACUCCGCCAUGAAUCACUCCCCUGUGAGGAACUACUGUCUUCACGCAGAAAGCGCCUAGCCAUGGCGUUAGUAUGAGUGUCGUACAGCCUCCAGGCC
54. HQ852468.1/1-119 ACCUGCCCCUAA-UA----GGGG-CGACACUCCGCCAUGAAUCACUCCCCUGUGAGGAACUACUGUCUUCACGCAGAAAGCGCCUAGCCAUGGCGUUAGUAUGAGUGUCGUACAGCCUCCAGGCC
55. HQ852467.1/1-119 ACCUGCCCCUAA-UA----GGGG-CGACACUCCGCCAUGAAUCACUCCCCUGUGAGGAACUACUGUCUUCACGCAGAAAGCGCCUAGCCAUGGCGUUAGUAUGAGUGUCGUACAGCCUCCAGGCC
56. HQ852466.1/1-119 ACCUGCCCCUAA-UA----GGGG-CGACACUCCGCCAUGAAUCACUCCCCUGUGAGGAACUACUGUCUUCACGCAGAAAGCGCCUAGCCAUGGCGUUAGUAUGAGUGUCGUACAGCCUCCAGGCC
57. HQ852465.1/1-119 ACCUGCCCCUAA-UA----GGGG-CGACACUCCGCCAUGAAUCACUCCCCUGUGAGGAACUACUGUCUUCACGCAGAAAGCGCCUAGCCAUGGCGUUAGUAUGAGUGUCGUACAGCCUCCAGGCC
58. HQ852464.1/1-119 ACCUGCCCCUAA-UA----GGGG-CGACACUCCGCCAUGAAUCACUCCCCUGUGAGGAACUACUGUCUUCACGCAGAAAGCGCCUAGCCAUGGCGUUAGUAUGAGUGUCGUACAGCCUCCAGGCC
59. HQ852463.1/1-119 ACCUGCCCCUAA-UA----GGGG-CGACACUCCGCCAUGAAUCACUCCCCUGUGAGGAACUACUGUCUUCACGCAGAAAGCGCCUAGCCAUGGCGUUAGUAUGAGUGUCGUACAGCCUCCAGGCC
60. HQ852462.1/1-119 ACCUGCCCCUAA-UA----GGGG-CGACACUCCGCCAUGAAUCACUCCCCUGUGAGGAACUACUGUCUUCACGCAGAAAGCGCCUAGCCAUGGCGUUAGUAUGAGUGUCGUACAGCCUCCAGGCC
61. HQ852461.1/1-119 ACCUGCCCCUAA-UA----GGGG-CGACACUCCGCCAUGAAUCACUCCCCUGUGAGGAACUACUGUCUUCACGCAGAAAGCGCCUAGCCAUGGCGUUAGUAUGAGUGUCGUACAGCCUCCAGGCC
62. HQ852460.1/1-119 ACCUGCCCCUAA-UA----GGGG-CGACACUCCGCCAUGAAUCACUCCCCUGUGAGGAACUACUGUCUUCACGCAGAAAGCGCCUAGCCAUGGCGUUAGUAUGAGUGUCGUACAGCCUCCAGGCC
63. HQ852459.1/1-119 ACCUGCCCCUAA-UA----GGGG-CGACACUCCGCCAUGAAUCACUCCCCUGUGAGGAACUACUGUCUUCACGCAGAAAGCGCCUAGCCAUGGCGUUAGUAUGAGUGUCGUACAGCCUCCAGGCC
64. HQ852458.1/1-119 ACCUGCCCCUAA-UA----GGGG-CGACACUCCGCCAUGAAUCACUCCCCUGUGAGGAACUACUGUCUUCACGCAGAAAGCGCCUAGCCAUGGCGUUAGUAUGAGUGUCGUACAGCCUCCAGGCC
65. HM049503.1/1-119 ACCUGCCCCUAA-UA----GGGG-CGACACUCCGCCAUGAAUCACUCCCCUGUGAGGAACUACUGUCUUCACGCAGAAAGCGCCUAGCCAUGGCGUUAGUAUGAGUGUCGUACAGCCUCCAGGCC
66. HQ852457.1/1-119 ACCUGCCCCUAA-UA----GGGG-CGACACUCCGCCAUGAAUCACUCCCCUGUGAGGAACUACUGUCUUCACGCAGAAAGCGCCUAGCCAUGGCGUUAGUAUGAGUGUCGUACAGCCUCCAGGCC
67. HQ852456.1/1-119 ACCUGCCCCUAA-UA----GGGG-CGACACUCCGCCAUGAAUCACUCCCCUGUGAGGAACUACUGUCUUCACGCAGAAAGCGCCUAGCCAUGGCGUUAGUAUGAGUGUCGUACAGCCUCCAGGCC
68. HQ852455.1/1-119 ACCUGCCCCUAA-UA----GGGG-CGACACUCCGCCAUGAAUCACUCCCCUGUGAGGAACUACUGUCUUCACGCAGAAAGCGCCUAGCCAUGGCGUUAGUAUGAGUGUCGUACAGCCUCCAGGCC
69. HQ852454.1/1-119 ACCUGCCCCUAA-UA----GGGG-CGACACUCCGCCAUGAAUCACUCCCCUGUGAGGAACUACUGUCUUCACGCAGAAAGCGCCUAGCCAUGGCGUUAGUAUGAGUGUCGUACAGCCUCCAGGCC
70. HQ852453.1/1-119 ACCUGCCCCUAA-UA----GGGG-CGACACUCCGCCAUGAAUCACUCCCCUGUGAGGAACUACUGUCUUCACGCAGAAAGCGCCUAGCCAUGGCGUUAGUAUGAGUGUCGUACAGCCUCCAGGCC
71. FJ230883.1/1-119 ACCUGCCCCUAA-UA----GGGG-CGACACUCCGCCAUGAAUCACUCCCCUGUGAGGAACUACUGUCUUCACGCAGAAAGCGCCUAGCCAUGGCGUUAGUAUGAGUGUCGUACAGCCUCCAGGCC
72. EU363761.1/1-119 ACCUGCCCCUAA-UA----GGGG-CGACACUCCGCCAUGAAUCACUCCCCUGUGAGGAACUACUGUCUUCACGCAGAAAGCGCCUAGCCAUGGCGUUAGUAUGAGUGUCGUACAGCCUCCAGGCC
73. FJ230884.1/1-119 ACCUGCCCCUAA-UA----GGGG-CGACACUCCGCCAUGAAUCACUCCCCUGUGAGGAACUACUGUCUUCACGCAGAAAGCGCCUAGCCAUGGCGUUAGUAUGAGUGUCGUACAGCCUCCAGGCC
74. FJ230882.1/1-119 ACCUGCCCCUAA-UA----GGGG-CGACACUCCGCCAUGAAUCACUCCCCUGUGAGGAACUACUGUCUUCACGCAGAAAGCGCCUAGCCAUGGCGUUAGUAUGAGUGUCGUACAGCCUCCAGGCC
75. FJ230881.1/1-119 ACCUGCCCCUAA-UA----GGGG-CGACACUCCGCCAUGAAUCACUCCCCUGUGAGGAACUACUGUCUUCACGCAGAAAGCGCCUAGCCAUGGCGUUAGUAUGAGUGUCGUACAGCCUCCAGGCC
76. FJ393024.1/1-119 ACCUGCCCCUAA-UA----GGGG-CGACACUCCGCCAUGAAUCACUCCCCUGUGAGGAACUACUGUCUUCACGCAGAAAGCGCCUAGCCAUGGCGUUAGUAUGAGUGUCGUACAGCCUCCAGGCC
77. EU204645.1/1-119 ACCUGCCCCUAA-UA----GGGG-CGACACUCCGCCAUGAAUCACUCCCCUGUGAGGAACUACUGUCUUCACGCAGAAAGCGCCUAGCCAUGGCGUUAGUAUGAGUGUCGUACAGCCUCCAGGCC
78. AB690460.1/1-119 ACCUGCCCCUAA-UA----GGGG-CGACACUCCGCCAUGAAUCACUCCCCUGUGAGGAACUACUGUCUUCACGCAGAAAGCGCCUAGCCAUGGCGUUAGUAUGAGUGUCGUACAGCCUCCAGGCC
79. AB047639.1/1-119 ACCUGCCCCUAA-UA----GGGG-CGACACUCCGCCAUGAAUCACUCCCCUGUGAGGAACUACUGUCUUCACGCAGAAAGCGCCUAGCCAUGGCGUUAGUAUGAGUGUCGUACAGCCUCCAGGCC
80. M67463.1/1-120 GCCAGCCCCCUG-AU----GGGGgCGACACUCCACCAUGAAUCACUCCCCUGUGAGGAACUACUGUCUUCACGCAGAAAGCGUCUAGCCAUGGCGUUAGUAUGAGUGUCGUGCAGCCUCCAGGAC
81. NC_004102.1/1-120 GCCAGCCCCCUG-AU----GGGGgCGACACUCCACCAUGAAUCACUCCCCUGUGAGGAACUACUGUCUUCACGCAGAAAGCGUCUAGCCAUGGCGUUAGUAUGAGUGUCGUGCAGCCUCCAGGAC
82. KJ439782.1/1-120 GCCAGCCCCCUG-AU----GGGGgCGACACUCCACCAUGAAUCACUCCCCUGUGAGGAACUACUGUCUUCACGCAGAAAGCGUCUAGCCAUGGCGUUAGUAUGAGUGUCGUGCAGCCUCCAGGAC
83. KJ439780.1/1-120 GCCAGCCCCCUG-AU----GGGGgCGACACUCCACCAUGAAUCACUCCCCUGUGAGGAACUACUGUCUUCACGCAGAAAGCGUCUAGCCAUGGCGUUAGUAUGAGUGUCGUGCAGCCUCCAGGAC
84. KJ439778.1/1-120 GCCAGCCCCCUG-AU----GGGGgCGACACUCCACCAUGAAUCACUCCCCUGUGAGGAACUACUGUCUUCACGCAGAAAGCGUCUAGCCAUGGCGUUAGUAUGAGUGUCGUGCAGCCUCCAGGAC
85. KJ439775.1/1-120 GCCAGCCCCCUG-AU----GGGGgCGACACUCCACCAUGAAUCACUCCCCUGUGAGGAACUACUGUCUUCACGCAGAAAGCGUCUAGCCAUGGCGUUAGUAUGAGUGUCGUGCAGCCUCCAGGAC
86. KJ439774.1/1-120 GCCAGCCCCCUG-AU----GGGGgCGACACUCCACCAUGAAUCACUCCCCUGUGAGGAACUACUGUCUUCACGCAGAAAGCGUCUAGCCAUGGCGUUAGUAUGAGUGUCGUGCAGCCUCCAGGAC
87. KJ439773.1/1-120 GCCAGCCCCCUG-AU----GGGGgCGACACUCCACCAUGAAUCACUCCCCUGUGAGGAACUACUGUCUUCACGCAGAAAGCGUCUAGCCAUGGCGUUAGUAUGAGUGUCGUGCAGCCUCCAGGAC
88. KJ439767.1/1-120 GCCAGCCCCCUG-AU----GGGGgCGACACUCCACCAUGAAUCACUCCCCUGUGAGGAACUACUGUCUUCACGCAGAAAGCGUCUAGCCAUGGCGUUAGUAUGAGUGUCGUGCAGCCUCCAGGAC
89. KC248198.1/1-120 GCCAGCCCCCUG-AU----GGGGgCGACACUCCACCAUGAAUCACUCCCCUGUGAGGAACUACUGUCUUCACGCAGAAAGCGUCUAGCCAUGGCGUUAGUAUGAGUGUCGUGCAGCCUCCAGGAC
90. KC248197.1/1-120 GCCAGCCCCCUG-AU----GGGGgCGACACUCCACCAUGAAUCACUCCCCUGUGAGGAACUACUGUCUUCACGCAGAAAGCGUCUAGCCAUGGCGUUAGUAUGAGUGUCGUGCAGCCUCCAGGAC
91. KC248196.1/1-120 GCCAGCCCCCUG-AU----GGGGgCGACACUCCACCAUGAAUCACUCCCCUGUGAGGAACUACUGUCUUCACGCAGAAAGCGUCUAGCCAUGGCGUUAGUAUGAGUGUCGUGCAGCCUCCAGGAC
92. KC248195.1/1-120 GCCAGCCCCCUG-AU----GGGGgCGACACUCCACCAUGAAUCACUCCCCUGUGAGGAACUACUGUCUUCACGCAGAAAGCGUCUAGCCAUGGCGUUAGUAUGAGUGUCGUGCAGCCUCCAGGAC
93. JF343780.2/1-120 GCCAGCCCCCUG-AU----GGGGgCGACACUCCACCAUGAAUCACUCCCCUGUGAGGAACUACUGUCUUCACGCAGAAAGCGUCUAGCCAUGGCGUUAGUAUGAGUGUCGUGCAGCCUCCAGGAC
94. AB520610.1/1-120 GCCAGCCCCCUG-AU----GGGGgCGACACUCCACCAUGAAUCACUCCCCUGUGAGGAACUACUGUCUUCACGCAGAAAGCGUCUAGCCAUGGCGUUAGUAUGAGUGUCGUGCAGCCUCCAGGAC
95. AF271632.1/1-120 GCCAGCCCCCUG-AU----GGGGgCGACACUCCACCAUGAAUCACUCCCCUGUGAGGAACUACUGUCUUCACGCAGAAAGCGUCUAGCCAUGGCGUUAGUAUGAGUGUCGUGCAGCCUCCAGGAC
96. D10749.1/1-120 GCCAGCCCCCUG-AU----GGGGgCGACACUCCACCAUGAAUCACUCCCCUGUGAGGAACUACUGUCUUCACGCAGAAAGCGUCUAGCCAUGGCGUUAGUAUGAGUGUCGUGCAGCCUCCAGGAC
97. EF621489.1/1-120 GCCAGCCCCCUG-AU----GGGGgCGACACUCCACCAUGAAUCACUCCCCUGUGAGGAACUACUGUCUUCACGCAGAAAGCGUCUAGCCAUGGCGUUAGUAUGAGUGUCGUGCAGCCUCCAGGAC
98. AF054249.1/1-120 GCCAGCCCCCUG-AU----GGGGgCGACACUCCACCAUGAAUCACUCCCCUGUGAGGAACUACUGUCUUCACGCAGAAAGCGUCUAGCCAUGGCGUUAGUAUGAGUGUCGUGCAGCCUCCAGGAC
99. AF054248.1/1-120 GCCAGCCCCCUG-AU----GGGGgCGACACUCCACCAUGAAUCACUCCCCUGUGAGGAACUACUGUCUUCACGCAGAAAGCGUCUAGCCAUGGCGUUAGUAUGAGUGUCGUGCAGCCUCCAGGAC
100. AF054247.1/1-120 GCCAGCCCCCUG-AU----GGGGgCGACACUCCACCAUGAAUCACUCCCCUGUGAGGAACUACUGUCUUCACGCAGAAAGCGUCUAGCCAUGGCGUUAGUAUGAGUGUCGUGCAGCCUCCAGGAC
101. M62321.1/1-120 GCCAGCCCCCUG-AU----GGGGgCGACACUCCACCAUGAAUCACUCCCCUGUGAGGAACUACUGUCUUCACGCAGAAAGCGUCUAGCCAUGGCGUUAGUAUGAGUGUCGUGCAGCCUCCAGGAC
102. KU180718.1/1-120 GCCAGCCCCCUG-AU----GGGGgCGACACUCCACCAUGAAUCACUCCCCUGUGAGGAACUACUGUCUUCACGCAGAAAGCGUCUAGCCAUGGCGUUAGUAUGAGUGUCGUGCAGCCUCCAGGAC
103. KU180714.1/1-120 GCCAGCCCCCUG-AU----GGGGgCGACACUCCACCAUGAAUCACUCCCCUGUGAGGAACUACUGUCUUCACGCAGAAAGCGUCUAGCCAUGGCGUUAGUAUGAGUGUCGUGCAGCCUCCAGGAC
104. KJ567647.1/1-118 GCCAGCCCCUUA-AC----GGGG-CGACACUCCACCAUG-AUCACUCCCCUGUGAGGAACUACUGUCUUCACGCAGAAAGCGUCUAGCCAUGGCGUUAGUAUGAGUGUCGUACAGCCUCCAGGAC
105. KJ439770.1/1-120 GCCAGCCCCCUG-AU----GGGGgCGACACUCCACCAUGGAUCACUCCCCUGUGAGGAACUACUGUCUUCACGCAGAAAGCGUCUAGCCAUGGCGUUAGUAUGAGUGUCGUACAGCCUCCAGGAC
106. AY045702.1/3-122 GCCAGCCCCCGA-UU----GGGGgCGACACUCCACCAUAGAUCACUCCCCUGUGAGGAACUACUGUCUUCACGCAGAAAGCGUCUAGCCAUGGCGUUAGUAUGAGUGUCGUGCAGCCUCCAGGAC
107. U16362.1/2-121 GCCAGCCCCCGA-UU----GGGGgCGACACUCCACCAUAGAUCACUCCCCUGUGAGGAACUACUGUCUUCACGCAGAAAGCGUCUAGCCAUGGCGUUAGUAUGAGUGUCGUGCAGCCUCCAGGAC
108. M84754.1/1-120 GCCAGCCCCCGA-UU----GGGGgCGACACUCCACCAUAGAUCACUCCCCUGUGAGGAACUACUGUCUUCACGCAGAAAGCGUCUAGCCAUGGCGUUAGUAUGAGUGUCGUGCAGCCUCCAGGAC
109. AB828701.1/1-120 GCCAGCCCCCGA-UU----GGGGgCGACACUCCACCAUAGAUCACUCCCCUGUGAGGAACUACUGUCUUCACGCAGAAAGCGUCUAGCCAUGGCGUUAGUAUGAGUGUCGUGCAGCCUCCAGGAC
110. AB828700.1/1-120 GCCAGCCCCCGA-UU----GGGGgCGACACUCCACCAUAGAUCACUCCCCUGUGAGGAACUACUGUCUUCACGCAGAAAGCGUCUAGCCAUGGCGUUAGUAUGAGUGUCGUGCAGCCUCCAGGAC
111. AB828699.1/1-120 GCCAGCCCCCGA-UU----GGGGgCGACACUCCACCAUAGAUCACUCCCCUGUGAGGAACUACUGUCUUCACGCAGAAAGCGUCUAGCCAUGGCGUUAGUAUGAGUGUCGUGCAGCCUCCAGGAC
112. JF343781.2/1-120 GCCAGCCCCCGA-UU----GGGGgCGACACUCCACCAUAGAUCACUCCCCUGUGAGGAACUACUGUCUUCACGCAGAAAGCGUCUAGCCAUGGCGUUAGUAUGAGUGUCGUGCAGCCUCCAGGAC
113. GU133617.1/1-120 GCCAGCCCCCGA-UU----GGGGgCGACACUCCACCAUAGAUCACUCCCCUGUGAGGAACUACUGUCUUCACGCAGAAAGCGUCUAGCCAUGGCGUUAGUAUGAGUGUCGUGCAGCCUCCAGGAC
114. AB049088.1/1-120 GCCAGCCCCCGA-UU----GGGGgCGACACUCCACCAUAGAUCACUCCCCUGUGAGGAACUACUGUCUUCACGCAGAAAGCGUCUAGCCAUGGCGUUAGUAUGAGUGUCGUGCAGCCUCCAGGAC
115. D14484.1/1-120 GCCAGCCCCCGA-UU----GGGGgCGACACUCCACCAUAGAUCACUCCCCUGUGAGGAACUACUGUCUUCACGCAGAAAGCGUCUAGCCAUGGCGUUAGUAUGAGUGUCGUGCAGCCUCCAGGAC
116. D11355.1/1-120 GCCAGCCCCCGA-UU----GGGGgCGACACUCCACCAUAGAUCACUCCCCUGUGAGGAACUACUGUCUUCACGCAGAAAGCGUCUAGCCAUGGCGUUAGUAUGAGUGUCGUGCAGCCUCCAGGAC
117. D11168.1/1-120 GCCAGCCCCCGA-UU----GGGGgCGACACUCCACCAUAGAUCACUCCCCUGUGAGGAACUACUGUCUUCACGCAGAAAGCGUCUAGCCAUGGCGUUAGUAUGAGUGUCGUGCAGCCUCCAGGAC
118. AF139594.2/1-120 GCCAGCCCCCGA-UU----GGGGgCGACACUCCACCAUAGAUCACUCCCCUGUGAGGAACUACUGUCUUCACGCAGAAAGCGUCUAGCCAUGGCGUUAGUAUGAGUGUCGUGCAGCCUCCAGGAC
119. AF176573.1/1-120 GCCAGCCCCCGA-UU----GGGGgCGACACUCCACCAUAGAUCACUCCCCUGUGAGGAACUACUGUCUUCACGCAGAAAGCGUCUAGCCAUGGCGUUAGUAUGAGUGUCGUGCAGCCUCCAGGAC
120. AB191333.1/1-120 GCCAGCCCCCGA-UU----GGGGgCGACACUCCACCAUAGAUCACUCCCCUGUGAGGAACUACUGUCUUCACGCAGAAAGCGUCUAGCCAUGGCGUUAGUAUGAGUGUCGUGCAGCCUCCAGGAC
121. AB442222.1/1-120 GCCAGCCCCCGA-UU----GGGGgCGACACUCCACCAUAGAUCACUCCCCUGUGAGGAACUACUGUCUUCACGCAGAAAGCGUCUAGCCAUGGCGUUAGUAUGAGUGUCGUGCAGCCUCCAGGAC
122. AB442220.1/1-120 GCCAGCCCCCGA-UU----GGGGgCGACACUCCACCAUAGAUCACUCCCCUGUGAGGAACUACUGUCUUCACGCAGAAAGCGUCUAGCCAUGGCGUUAGUAUGAGUGUCGUGCAGCCUCCAGGAC
123. AB442221.1/1-120 GCCAGCCCCCGA-UU----GGGGgCGACACUCCACCAUAGAUCACUCCCCUGUGAGGAACUACUGUCUUCACGCAGAAAGCGUCUAGCCAUGGCGUUAGUAUGAGUGUCGUGCAGCCUCCAGGAC
124. AB435162.2/1-120 GCCAGCCCCCGA-UU----GGGGgCGACACUCCACCAUAGAUCACUCCCCUGUGAGGAACUACUGUCUUCACGCAGAAAGCGUCUAGCCAUGGCGUUAGUAUGAGUGUCGUGCAGCCUCCAGGAC
125. AB426117.1/1-120 GCCAGCCCCCGA-UU----GGGGgCGACACUCCACCAUAGAUCACUCCCCUGUGAGGAACUACUGUCUUCACGCAGAAAGCGUCUAGCCAUGGCGUUAGUAUGAGUGUCGUGCAGCCUCCAGGAC
126. AB249644.1/1-120 GCCAGCCCCCGA-UU----GGGGgCGACACUCCACCAUAGAUCACUCCCCUGUGAGGAACUACUGUCUUCACGCAGAAAGCGUCUAGCCAUGGCGUUAGUAUGAGUGUCGUGCAGCCUCCAGGAC
127. D10750.1/1-120 GCCAGCCCCCGA-UU----GGGGgCGACACUCCACCAUAGAUCACUCCCCUGUGAGGAACUACUGUCUUCACGCAGAAAGCGUCUAGCCAUGGCGUUAGUAUGAGUGUCGUGCAGCCUCCAGGAC
128. D13558.1/1-120 GCCAGCCCCCGA-UU----GGGGgCGACACUCCACCAUAGAUCACUCCCCUGUGAGGAACUACUGUCUUCACGCAGAAAGCGUCUAGCCAUGGCGUUAGUAUGAGUGUCGUGCAGCCUCCAGGAC
129. D10934.1/1-120 GCCAGCCCCCGA-UU----GGGGgCGACACUCCACCAUAGAUCACUCCCCUGUGAGGAACUACUGUCUUCACGCAGAAAGCGUCUAGCCAUGGCGUUAGUAUGAGUGUCGUGCAGCCUCCAGGAC
130. AJ238799.1/1-120 GCCAGCCCCCGA-UU----GGGGgCGACACUCCACCAUAGAUCACUCCCCUGUGAGGAACUACUGUCUUCACGCAGAAAGCGUCUAGCCAUGGCGUUAGUAUGAGUGUCGUGCAGCCUCCAGGAC
131. AB080299.1/1-120 GCCAGCCCCCGA-UU----GGGGgCGACACUCCACCAUAGAUCACUCCCCUGUGAGGAACUACUGUCUUCACGCAGAAAGCGUCUAGCCAUGGCGUUAGUAUGAGUGUCGUGCAGCCUCCAGGAC
132. D85516.1/1-120 GCCAGCCCCCGA-UU----GGGGgCGACACUCCACCAUAGAUCACUCCCCUGUGAGGAACUACUGUCUUCACGCAGAAAGCGUCUAGCCAUGGCGUUAGUAUGAGUGUCGUGCAGCCUCCAGGAC
133. KU180728.1/1-120 GCCAGCCCCCGA-UU----GGGGgCGACACUCCACCAUAGAUCACUCCCCUGUGAGGAACUACUGUCUUCACGCAGAAAGCGUCUAGCCAUGGCGUUAGUAUGAGUGUCGUGCAGCCUCCAGGAC
134. KU180725.1/1-120 GCCAGCCCCCGA-UU----GGGGgCGACACUCCACCAUAGAUCACUCCCCUGUGAGGAACUACUGUCUUCACGCAGAAAGCGUCUAGCCAUGGCGUUAGUAUGAGUGUCGUGCAGCCUCCAGGAC
135. KU180711.1/1-120 GCCAGCCCCCGA-UU----GGGGgCGACACUCCACCAUAGAUCACUCCCCUGUGAGGAACUACUGUCUUCACGCAGAAAGCGUCUAGCCAUGGCGUUAGUAUGAGUGUCGUGCAGCCUCCAGGAC
136. KM587615.1/1-120 GCCAGCCCCCGA-UU----GGGGgCGACACUCCACCAUAGAUCACUCCCCUGUGAGGAACUACUGUCUUCACGCAGAAAGCGUCUAGCCAUGGCGUUAGUAUGAGUGUCGUGCAGCCUCCAGGAC
137. AB429050.1/1-120 GCCAGCCCCCGA-UU----GGGGgCGACACUCCACCAUAGAUCACUCCCCUGUGAGGAACUACUGUCUUCACGCAGAAAGCGUCUAGCCAUGGCGUUAGUAUGAGUGUCGUGCAGCCUCCAGGAC
138. JC286039.1/1-120 GCCAGCCCCCGA-UU----GGGGgCGACACUCCACCAUAGAUCACUCCCCUGUGAGGAACUACUGUCUUCACGCAGAAAGCGUCUAGCCAUGGCGUUAGUAUGAGUGUCGUGCAGCCUCCAGGAC
139. KU180716.1/1-120 GCCAGCCCCCGA-UU----GGGGgCGACACUCCACCAUGGAUCACUCCCCUGUGAGGAACUACUGUCUUCACGCAGAAAGCGUCUAGCCAUGGCGUUAGUAUGAGUGUCGUGCAGCCUCCAGGAC
140. KJ439772.1/1-120 GCCAGCCCCCUG-AU----GGGGgCGACACUCCACCAUAGAUCACUCCCCUGUGAGGAACUACUGUCUUCACGCAGAAAGCGUCUAGCCAUGGCGUUAGUAUGAGUGUCGUGCAGCCUCCAGGAC
141. KJ439769.1/1-120 GCCAGCCCCCUG-AU----GGGGgCGACACUCCACCAUAGAUCACUCCCCUGUGAGGAACUACUGUCUUCACGCAGAAAGCGUCUAGCCAUGGCGUUAGUAUGAGUGUCGUGCAGCCUCCAGGAC
142. EU857431.1/1-120 GCCAGCCCCCUG-AU----GGGGgCGACACUCCACCAUAGAUCACUCCCCUGUGAGGAACUACUGUCUUCACGCAGAAAGCGUCUAGCCAUGGCGUUAGUAUGAGUGUCGUGCAGCCUCCAGGAC
143. LC011929.1/1-120 GCCAGCCCCCUG-AU----GGGGgCGACACUCCACCAUAGAUCACUCCCCUGUGAGGAACUACUGUCUUCACGCAGAAAGCGUCUAGCCAUGGCGUUAGUAUGAGUGUCGUGCAGCCUCCAGGAC
144. LC011931.1/1-120 GCCAGCCCCCUG-AU----GGGGgCGACACUCCACCAUAGAUCACUCCCCUGUGAGGAACUACUGUCUUCACGCAGAAAGCGUCUAGCCAUGGCGUUAGUAUGAGUGUCGUGCAGCCUCCAGGAC
145. LC011930.1/1-120 GCCAGCCCCCUG-AU----GGGGgCGACACUCCACCAUAGAUCACUCCCCUGUGAGGAACUACUGUCUUCACGCAGAAAGCGUCUAGCCAUGGCGUUAGUAUGAGUGUCGUGCAGCCUCCAGGAC
146. LC011928.1/1-120 GCCAGCCCCCUG-AU----GGGGgCGACACUCCACCAUAGAUCACUCCCCUGUGAGGAACUACUGUCUUCACGCAGAAAGCGUCUAGCCAUGGCGUUAGUAUGAGUGUCGUGCAGCCUCCAGGAC
147. LC011927.1/1-120 GCCAGCCCCCUG-AU----GGGGgCGACACUCCACCAUAGAUCACUCCCCUGUGAGGAACUACUGUCUUCACGCAGAAAGCGUCUAGCCAUGGCGUUAGUAUGAGUGUCGUGCAGCCUCCAGGAC
148. AB691953.1/1-120 GCCAGCCCCCUG-AU----GGGGgCGACACUCCACCAUAGAUCACUCCCCUGUGAGGAACUACUGUCUUCACGCAGAAAGCGUCUAGCCAUGGCGUUAGUAUGAGUGUCGUGCAGCCUCCAGGAC
149. D30613.1/1-120 GCCAGCCCCCUG-AU----GGGGgCGACACUCCACCAUAGAUCACUCCCCUGUGAGGAACUACUGUCUUCACGCAGAAAGCGUCUAGCCAUGGCGUUAGUAUGAGUGUCGUGCAGCCUCCAGGAC
150. KJ470624.1/1-118 GCCAGCCCCUUA-AC----GGGG-CGACACUCCACCAUG-AUCACUCCCCUGUGAGGAACUACUGUCUUCACGCAGAAAGCGUCUAGCCAUGGCGUUAGUAUGAGUGUCGUGCAGCCUCCAGGAC
151. KJ439777.1/1-120 GCCAGCCCCCUG-AU----GGGGgCGACACUCCACCAUGGAUCACUCCCCUGUGAGGAACUACUGUCUUCACGCAGAAAGCGUCUAGCCAUGGCGUUAGUAUGAGUGUCGUGCAGCCUCCAGGAC
152. FN435993.1/1-120 GCCAGCCCCCUG-UU----GGGGgCGACACUCCACCAUAGAUCACUCCCCUGUGAGGAACUACUGUCUUCACGCAGAAAGCGUCUAGCCAUGGCGUUAGUAUGAGUGUCGUGCAGCCUCCAGGAC
153. JF735114.1/1-119 ACCUGCCCCUAA-UA----GGGG-CGACACUCCACCAUGGACCACUCCCCUGUGAGGAACUACUGUCUUCACGCAGAAAGCGUCUAGCCAUGGCGUUAGUAUGAGUGUCGUACAGCCUCCAGGCC
154. KJ567648.1/1-118 GCCAGCCCCU-A-AU----GGGG-CGACACUCCACCAUGAAUCACUCCCCUGUGAGGAACUACUGUCUUCACGCAGAAAGCGUCUAGCCAUGGCGUUAGUAUGAGUGUCGUGCAGCCUCCAGGAC
155. KJ439771.1/1-120 GCCAGCCCCCUG-AU----GGGGgCGACACUCCACCAUGAAUCACUCCCCUGUGAGGAACUACUGUCUUCACGCAGAAAGCGUCUAGCCAUGGCGUUAGUAUGAGUGUCGUGCAGCCUCCAGGAU
156. KC248199.1/1-120 GCCAGCCCCCUG-AU----GGGGgCGACACUCCACCAUGAAUCACUCCCCUGUGAGGAACUACUGUCUUCACGCAGAAAGCGUCUAGCCAUGGCGUUAGUAUGAGUGUCGUGCAGCCUCCAGGAU
157. KJ678800.1/1-118 GCCAGCCCCUUA-AC----GGGG-CGACACUCCACCAUU-AUCACUCCCCUGUGAGGAACUACUGUCUUCACGCAGAAAGCGUCUAGCCAUGGCGUUAGUAUGAGUGUCGUACAGCCUCCAGGAC
158. KM504109.1/1-118 GCCAGCCCCUUA-AC----GGGG-CGACACUCCACCAUG-AUCACUCCCCUGUGAGGAACUACUGUCUUCACGCAGAAAGCGUCUAGCCAUGGCGUUAGUAUGAGUGUCGUACAGCCUCCAGGAU
159. JF735110.1/1-119 ACCUGCCCCCUA-UA----GGGG-CGACACUCCGCCAUGAAUCACUCCCCUGUGAGGAACUACUGUCUUCACGCAGAAAGCGUCUAGCCAUGGCGUUAGUAUGAGUGUCGAACAGCCUCCAGGCC
160. KJ439768.1/1-120 GCCAGCCCCCUG-AU----GGGGgCGACACUCCACCAUAGAUCACUCCCCUGUGAGGAACUACUGUCUUCACGCAGAAAGCGUCUAGCCAUGGCGUUAGUAUGAGUGUCGUGCAGCCUCCAGGAU
161. KJ470623.1/1-118 GCCAGCCCCUUA-AC----GGGG-CGACACUCCACCAUG-AUCACUCCCCUGUGAGGAACUACUGUCUUCACGCAGAAAGCGUCUAGCCAUGGCGUUAGUAUGAGUGUCGUGCAGCCUCCAGGAU
162. KJ470620.1/1-118 GCCAGCCCCUUA-AC----GGGG-CGACACUCCACCAUG-AUCACUCCCCUGUGAGGAACUACUGUCUUCACGCAGAAAGCGUCUAGCCAUGGCGUUAGUAUGAGUGUCGUGCAGCCUCCAGGAU
163. KM587616.1/1-119 ACCCGCCCCUAG-UA----GGGG-CGACACUCCGCCAUGAAUCACUCCCCUGUGAGGAACUACUGUCUUCACGCAGAAAGCGUCUAGCCAUGGCGUUAGUAUGAGUGUCGUACAGCCUCCAGGCC
164. JF735113.1/1-119 ACCUGCCCCUAA-UA----GGGG-CGACACUCCACCAUGAUUCACUCCCCUGUGAGGAACUACUGUCUUCACGCAGAAAGCGCCUAGCCAUGGCGUUAGUAUGAGUGUCGUGCAGCCUCCAGGCC
165. U89019.1/1-120 GCCAGCCCCCGA-UU----GGGGgCGACACUCCACCAUAGAUCACUCCCCUGUGAGGAACUACUGUCUUCACGCAGAAAGCGUCUAGCCAUGGCGUUAGUAUGAGUGUCGUGCAGCCUCCAGGUC
166. AB442219.1/1-120 GCCAGCCCCCGA-UU----GGGGgCGACACUCCACCAUAGAUCACUCCCCUGUGAGGAACUACUGUCUUCACGCAGAAAGCGUCUAGCCAUGGCGUUAGUAUGAGUGUCGUGCAGCCUCCAGGUC
167. S62220.1/1-119 ACCCGCCCCGAU-UG----GGGG-CGACACUCCACCAUAGAUCACUCCCCUGUGAGGAACUACUGUCUUCACGCAGAAAGCGUCUAGCCAUGGCGUUAGUAUGAGUGUCGUGCAGCCUCCAGGAC
168. AY651061.1/1-120 GCCAGCCCCCUG-AU----GGGGgCGACACUCCGCCAUGAAUCACUCCCCUGUGAGGAACUACUGUCUUCACGCAGAAAGCGUCUAGCCAUGGCGUUAGUAUGAGUGUCGUGCAGCCUCCAGGAC
169. D14853.1/1-120 GCCAGCCCCCUG-AU----GGGGgCGACACUCCGCCAUGAAUCACUCCCCUGUGAGGAACUACUGUCUUCACGCAGAAAGCGUCUAGCCAUGGCGUUAGUAUGAGUGUCGUGCAGCCUCCAGGAC
170. NC_009827.1/1-118 GCCAGCCCCUUA-AC----GGGG-CGACACUCCACCAUG-AUCACUCCCCUGUGAGGAACUACUGUCUUCACGCAGAAAGCGUCUAGCCAUGGCGUUAGUAUGAGUGUCGUGCAGCCUCCAGGCC
171. D84262.2/1-118 GCCAGCCCCUUA-AC----GGGG-CGACACUCCACCAUG-AUCACUCCCCUGUGAGGAACUACUGUCUUCACGCAGAAAGCGUCUAGCCAUGGCGUUAGUAUGAGUGUCGUGCAGCCUCCAGGCC
172. U45476.1/1-120 GCCAGCCCCCGA-UU----GGGGgCGACACUCCACCAUAGAUCACUCCCCUGUGAGGAACUACUGUCUUCACGCAGAAAGCGUCUAGCCAUGGCGUUAGUAUGAGUGUCGUGCAGCCUCCAGGGC
173. KJ925149.1/1-118 ACCCGCCCCUUA-UU----GGGG-CGACACUCCACCAUG-AUCACUCCCCUGUGAGGAACUACUGUCUUCACGCAGAAAGCGUCUAGCCAUGGCGUUAGUAUGAGUGUCGAACAGCCUCCAGGAC
174. JF343786.2/1-118 ACCCGCCCCUUA-UU----GGGG-CGACACUCCACCAUG-AUCACUCCCCUGUGAGGAACUACUGUCUUCACGCAGAAAGCGUCUAGCCAUGGCGUUAGUAUGAGUGUCGAACAGCCUCCAGGAC
175. KM587626.1/1-118 ACCCGCCCCUUA-UU----GGGG-CGACACUCCACCAUG-AUCACUCCCCUGUGAGGAACUACUGUCUUCACGCAGAAAGCGUCUAGCCAUGGCGUUAGUAUGAGUGUCGAACAGCCUCCAGGAC
176. JF735117.1/1-119 ACCUGCCCCUAA-UA----GGGG-CGACACUCCGCCAUGAACCACUCCCCUGUGAGGAACUACUGUCUUCACGCAGAAAGCGUCUAGCCAUGGCGUUAGUAUGAGUGUCGUACAGCCUCCAGGCC
177. AF169004.1/1-119 ACCCGCCCCUAA-UA----GGGG-CGACACUCCGCCAUGAACCACUCCCCUGUGAGGAACUACUGUCUUCACGCAGAAAGCGUCUAGCCAUGGCGUUAGUAUGAGUGUCGUACAGCCUCCAGGCC
178. AF169003.1/1-119 ACCCGCCCCUAA-UA----GGGG-CGACACUCCGCCAUGAACCACUCCCCUGUGAGGAACUACUGUCUUCACGCAGAAAGCGUCUAGCCAUGGCGUUAGUAUGAGUGUCGUACAGCCUCCAGGCC
179. D00944.1/1-119 ACCCGCCCCUAA-UA----GGGG-CGACACUCCGCCAUGAACCACUCCCCUGUGAGGAACUACUGUCUUCACGCAGAAAGCGUCUAGCCAUGGCGUUAGUAUGAGUGUCGUACAGCCUCCAGGCC
180. U01214.1/2-121 GCCAGCCCCCGA-UU----GGGGgCGACACUCCACCAUAGAUCACUCCCCUGUGAGGAACUAUUGUCUUCACGCAGAAAGCGUCUAGCCAUGGCGUUAGUAUGAGUGUCGUGCAACCUCCAGGAC
181. KJ678816.1/1-118 GCCAGCCCCUUA-AC----GGGG-CGACACUCCACCAUU-AUCACUCCCCUGUGAGGAACUACUGUCUUCACGCAGAAAGCGUCUAGCCAUGGCGUUAGUAUGAGUGUCGUACAGCCUCCAGGCC
182. KJ678810.1/1-118 GCCAGCCCCUUA-AC----GGGG-CGACACUCCACCAUU-AUCACUCCCCUGUGAGGAACUACUGUCUUCACGCAGAAAGCGUCUAGCCAUGGCGUUAGUAUGAGUGUCGUACAGCCUCCAGGCC
183. KJ678809.1/1-118 GCCAGCCCCUUA-AC----GGGG-CGACACUCCACCAUU-AUCACUCCCCUGUGAGGAACUACUGUCUUCACGCAGAAAGCGUCUAGCCAUGGCGUUAGUAUGAGUGUCGUACAGCCUCCAGGCC
184. KJ678799.1/1-118 GCCAGCCCCUUA-AC----GGGG-CGACACUCCACCAUU-AUCACUCCCCUGUGAGGAACUACUGUCUUCACGCAGAAAGCGUCUAGCCAUGGCGUUAGUAUGAGUGUCGUACAGCCUCCAGGCC
185. KJ678795.1/1-118 GCCAGCCCCUUA-AC----GGGG-CGACACUCCACCAUU-AUCACUCCCCUGUGAGGAACUACUGUCUUCACGCAGAAAGCGUCUAGCCAUGGCGUUAGUAUGAGUGUCGUACAGCCUCCAGGCC
186. KJ678794.1/1-118 GCCAGCCCCUUA-AC----GGGG-CGACACUCCACCAUU-AUCACUCCCCUGUGAGGAACUACUGUCUUCACGCAGAAAGCGUCUAGCCAUGGCGUUAGUAUGAGUGUCGUACAGCCUCCAGGCC
187. KJ678791.1/1-118 GCCAGCCCCUUA-AC----GGGG-CGACACUCCACCAUU-AUCACUCCCCUGUGAGGAACUACUGUCUUCACGCAGAAAGCGUCUAGCCAUGGCGUUAGUAUGAGUGUCGUACAGCCUCCAGGCC
188. KJ678783.1/1-118 GCCAGCCCCUUA-AC----GGGG-CGACACUCCACCAUU-AUCACUCCCCUGUGAGGAACUACUGUCUUCACGCAGAAAGCGUCUAGCCAUGGCGUUAGUAUGAGUGUCGUACAGCCUCCAGGCC
189. KJ678782.1/1-118 GCCAGCCCCUUA-AC----GGGG-CGACACUCCACCAUU-AUCACUCCCCUGUGAGGAACUACUGUCUUCACGCAGAAAGCGUCUAGCCAUGGCGUUAGUAUGAGUGUCGUACAGCCUCCAGGCC
190. KJ678781.1/1-118 GCCAGCCCCUUA-AC----GGGG-CGACACUCCACCAUU-AUCACUCCCCUGUGAGGAACUACUGUCUUCACGCAGAAAGCGUCUAGCCAUGGCGUUAGUAUGAGUGUCGUACAGCCUCCAGGCC
191. KJ678754.1/1-118 GCCAGCCCCUUA-AC----GGGG-CGACACUCCACCAUU-AUCACUCCCCUGUGAGGAACUACUGUCUUCACGCAGAAAGCGUCUAGCCAUGGCGUUAGUAUGAGUGUCGUACAGCCUCCAGGCC
192. KJ678745.1/1-118 GCCAGCCCCUUA-AC----GGGG-CGACACUCCACCAUU-AUCACUCCCCUGUGAGGAACUACUGUCUUCACGCAGAAAGCGUCUAGCCAUGGCGUUAGUAUGAGUGUCGUACAGCCUCCAGGCC
193. KJ678744.1/1-118 GCCAGCCCCUUA-AC----GGGG-CGACACUCCACCAUU-AUCACUCCCCUGUGAGGAACUACUGUCUUCACGCAGAAAGCGUCUAGCCAUGGCGUUAGUAUGAGUGUCGUACAGCCUCCAGGCC
194. KJ925147.1/1-118 ACCCGCCCCUUA-UU----GGGG-CGACACUCCACCAUG-AUCACUCCCCUGUGAGGAACUACUGUCUUCACGCAGAAAGCAUCUAGCCAUGGUGUUAGUAUGAGUGUCGUACAGCCUCCAGGAC
195. LC150893.1/1-120 GCCAGCCCCCUG-AU----GGGGgCGACACUCCGCCAUGAAUCACUCCCCUGUGAGGAACUACUGUCUUCACGCAGAAAGCGUCUAGCCAUGGCGUUAGUAUGAGUGUCGUACAGCCUCCAGGCC
196. D89815.1/1-120 GCCAGCCCCCUG-AU----GGGGgCGACACUCCACCAUAGAUCACCCCCCUGUGAGGAACUACUGUCUUCACGCAGAAAGCGUCUAGCCAUGGCGUUAGUAUGAGUGUCGUGCAGCCUCCAGGAC
197. KY620348.1/2-121 GCCAGCCCCCUG-AU----GGGGgCGACACUCCGCCAUGAAUCACUCCCCUGUGAGGAACUACUGUCUUCACGCAGAAAGCGUCUAGCCAUGGCGUUAGUAUGAGUGUCGUGCAGCCUCCAGGCC
198. AY460204.1/1-120 GCCAGCCCCCGA-UU----GGGGgCGACACUCCACCAUGAACCACUCCCCUGUGAGGAACUACUGUCUUCACGCAGAAAGCGUCUAGCCAUGGCGUUAGUAUGAGUGUCGUACAGCCUCCAGGCC
199. JQ745651.1/1-120 GCCCGCCCCCUG-AU----GGGGgCGACACUCCGCCAUGAAUCACUCCCCUGUGAGGAACUACUGUCUUCACGCAGAAAGCGUCUAGCCAUGGCGUUAGUAUGAGUGUCGUACAGCCUCCAGGCC
200. JF343783.1/1-120 GCCCGCCCCCUG-AU----GGGGgCGACACUCCGCCAUGAAUCACUCCCCUGUGAGGAACUACUGUCUUCACGCAGAAAGCGUCUAGCCAUGGCGUUAGUAUGAGUGUCGUACAGCCUCCAGGCC
201. AB558135.2/1-120 GCCCGCCCCCUG-AU----GGGGgCGACACUCCGCCAUGAAUCACUCCCCUGUGAGGAACUACUGUCUUCACGCAGAAAGCGUCUAGCCAUGGCGUUAGUAUGAGUGUCGUACAGCCUCCAGGCC
202. AB030907.1/1-120 GCCCGCCCCCUG-AU----GGGGgCGACACUCCGCCAUGAAUCACUCCCCUGUGAGGAACUACUGUCUUCACGCAGAAAGCGUCUAGCCAUGGCGUUAGUAUGAGUGUCGUACAGCCUCCAGGCC
203. AB677533.1/1-120 GCCCGCCCCCUG-AU----GGGGgCGACACUCCGCCAUGAAUCACUCCCCUGUGAGGAACUACUGUCUUCACGCAGAAAGCGUCUAGCCAUGGCGUUAGUAUGAGUGUCGUACAGCCUCCAGGCC
204. AB677532.1/1-120 GCCCGCCCCCUG-AU----GGGGgCGACACUCCGCCAUGAAUCACUCCCCUGUGAGGAACUACUGUCUUCACGCAGAAAGCGUCUAGCCAUGGCGUUAGUAUGAGUGUCGUACAGCCUCCAGGCC
205. AB677528.1/1-120 GCCCGCCCCCUG-AU----GGGGgCGACACUCCGCCAUGAAUCACUCCCCUGUGAGGAACUACUGUCUUCACGCAGAAAGCGUCUAGCCAUGGCGUUAGUAUGAGUGUCGUACAGCCUCCAGGCC
206. AB677527.1/1-120 GCCCGCCCCCUG-AU----GGGGgCGACACUCCGCCAUGAAUCACUCCCCUGUGAGGAACUACUGUCUUCACGCAGAAAGCGUCUAGCCAUGGCGUUAGUAUGAGUGUCGUACAGCCUCCAGGCC
207. KY620341.1/2-121 GCCCGCCCCCUG-AU----GGGGgCGACACUCCGCCAUGAAUCACUCCCCUGUGAGGAACUACUGUCUUCACGCAGAAAGCGUCUAGCCAUGGCGUUAGUAUGAGUGUCGUACAGCCUCCAGGCC
208. KU180730.1/1-120 GCCCGCCCCCUG-AU----GGGGgCGACACUCCGCCAUGAAUCACUCCCCUGUGAGGAACUACUGUCUUCACGCAGAAAGCGUCUAGCCAUGGCGUUAGUAUGAGUGUCGUACAGCCUCCAGGCC
209. KU180729.1/1-120 GCCCGCCCCCUG-AU----GGGGgCGACACUCCGCCAUGAAUCACUCCCCUGUGAGGAACUACUGUCUUCACGCAGAAAGCGUCUAGCCAUGGCGUUAGUAUGAGUGUCGUACAGCCUCCAGGCC
210. KU180723.1/1-120 GCCCGCCCCCUG-AU----GGGGgCGACACUCCGCCAUGAAUCACUCCCCUGUGAGGAACUACUGUCUUCACGCAGAAAGCGUCUAGCCAUGGCGUUAGUAUGAGUGUCGUACAGCCUCCAGGCC
211. KM587618.1/1-120 GCCCGCCCCCUG-AU----GGGGgCGACACUCCGCCAUGAAUCACUCCCCUGUGAGGAACUACUGUCUUCACGCAGAAAGCGUCUAGCCAUGGCGUUAGUAUGAGUGUCGUACAGCCUCCAGGCC
212. KM587617.1/1-120 GCCCGCCCCCUG-AU----GGGGgCGACACUCCGCCAUGAAUCACUCCCCUGUGAGGAACUACUGUCUUCACGCAGAAAGCGUCUAGCCAUGGCGUUAGUAUGAGUGUCGUACAGCCUCCAGGCC
213. KJ925148.1/1-118 ACCCGCCCCUUA-UU----GGGG-CGACACUCCACCAUG-AUCACUCCCCUGUGAGGAACUACUGUCUUCACGCAGAAAGCGUCUAGCCAUGGCGUUAGUAUGAGUGUCGAACAGCCUCCAGGCC
214. KY620338.1/13-132 ACCCGCCCCCUU-AU----GGGGgCGACACUCCGCCAUGAAUCACUCCCCUGUGAGGAACUACUGUCUUCACGCAGAAAGCGUCUAGCCAUGGCGUUAGUAUGAGUGUCGUACAGCCUCCAGGCC
215. KJ678792.1/1-118 GCCAGCCCCUUA-AC----GGGG-CGACACUCCACCAUU-RUCACUCCCCUGUGAGGAACUACUGUCUUCACGCAGAAAGCGUCUAGCCAUGGCGUUAGUAUGAGUGUCGUACAGCCUCCAGGCC
216. JF343787.2/1-118 GCCAGCCCCUUA-AC----GGGG-CGACACUCCGCCAUU-AUCACUCCCCUGUGAGGAACUACUGUCUUCACGCAGAAAGCGUCUAGCCAUGGCGUUAGUAUGAGUGUCGUACAGCCUCCAGGCC
217. KJ470625.1/1-118 GCCAGCCCCUUA-AC----GGGG-SGACACUCCACCAUG-AUCACUCCCCUGUGAGGAACUACUGUCUUCACGCAGAAAGCGUCUAGCCAUGGCGUUAGUAUGAGUGUCGUGCAGCCUCCAGGAU
218. KY620328.1/3-121 UUCCGCCUCUAA-AA----GGGG-CGACACUCCGCCAUGAAUCACUCCCCUGUGAGGAACUACUGUCUUCACGCAGAAAGCGUCUAGCCAUGGCGUUAGUAUGAGUGUCGUACAGCCUCCAGGCC
219. KY620332.1/8-126 GCCCGCCCCUCC-AA----GGGG-CGACACUCCGCCAUGAACCACUCCCCUGUGAGGAACUACUGUCUUCACGCAGAAAGCGUCUAGCCAUGGCGUUAGUAUGAGUGUCGUACAGCCUCCAGGCC
220. KJ470621.1/1-118 GCCAGCCCCUUA-AC----GGGG-SGACACUCCACCAUG-AUCACUCCCCUGUGAGGAACUACUGUCUUCACGCAGAAAGCGUCUAGCCAUGGCGUUAGUAUGAGUGUCGUACAGCCUCCAGGCC
221. EF424628.1/1-117 GCCAGCCCCU-A-AU----GGGG-CGACACUCCACCAUG-AUCACUCCCCUGUGAGGAACUACUGUCUUCACGCAGAAAGCGUCUAGCCAUGGCGUUAGUAUGAGUGUCGUACAGCCUCCAGGAC
222. KJ439776.1/1-120 GCCAGCCCCCUG-AU----GGGGgCGACACUCCACCAUGAAUCACUCCCCCGUGAGGAACUACUGUCUUCACGCAGAAAGCGUCUAGCCAUGGCGUUAGUAUGAGUGUCGUACAGCCUCCAGGAC
223. KJ470622.1/1-118 GCCAGCCCCUUA-AC----GGGG-SGACACUCCACCAUR-AUCACUCCCCUGUGAGGAACUACUGUCUUCACGCAGAAAGCGUCUAGCCAUGGCGUUAGUAUGAGUGUCGUGCAGCCUCCAGGCC
224. KJ567652.1/1-117 GCCAGCCCCU-A-AU----GGGG-CGACACUCCACCAUA-AUCACUCCCCUGUGAGGAACUACUGUCUUCACGCAGAAAGCGUCUAGCCAUGGCGUUAGUAUGAGUGUCGUGCAGCCUCCAGGAC
225. KY620337.1/6-124 CCUGGCCCCUGA-UG----GGGG-CGACACUCCGCCAUGAAUCACUCCCCUGUGAGGAACUACUGUCUUCACGCAGAAAGCGUCUAGCCAUGGCGUUAGUAUGAGUGUCGUACAGCCUCCAGGCC
226. KJ567649.1/1-117 GCCAGCCCCU-A-AU----GGGG-CGACACUCCACCAUG-AUCACUCCCCUGUGAGGAACUACUGUCUUCACGCAGAAAGCGUCUAGCCAUGGCGUUAGUAUGAGUGUCGUGCAGCCUCCAGGAC
227. KJ567645.1/1-117 GCCAGCCCCU-A-AU----GGGG-CGACACUCCACCAUG-AUCACUCCCCUGUGAGGAACUACUGUCUUCACGCAGAAAGCGUCUAGCCAUGGCGUUAGUAUGAGUGUCGUGCAGCCUCCAGGAC
228. DQ278892.1/1-117 GCCAGCCCCU-A-AU----GGGG-CGACACUCCACCAUG-AUCACUCCCCUGUGAGGAACUACUGUCUUCACGCAGAAAGCGUCUAGCCAUGGCGUUAGUAUGAGUGUCGUGCAGCCUCCAGGAC
229. EF424627.1/1-117 GCCAGCCCCU-A-AU----GGGG-CGACACUCCACCAUG-AUCACUCCCCUGUGAGGAACUACUGUCUUCACGCAGAAAGCGUCUAGCCAUGGCGUUAGUAUGAGUGUCGUGCAGCCUCCAGGAC
230. EF632070.1/1-117 GCCAGCCCCU-A-AU----GGGG-CGACACUCCACCAUG-AUCACUCCCCUGUGAGGAACUACUGUCUUCACGCAGAAAGCGUCUAGCCAUGGCGUUAGUAUGAGUGUCGUGCAGCCUCCAGGAC
231. DQ835770.1/1-117 GCCAGCCCCU-A-AU----GGGG-CGACACUCCACCAUG-AUCACUCCCCUGUGAGGAACUACUGUCUUCACGCAGAAAGCGUCUAGCCAUGGCGUUAGUAUGAGUGUCGUGCAGCCUCCAGGAC
232. DQ835769.1/1-117 GCCAGCCCCU-A-AU----GGGG-CGACACUCCACCAUG-AUCACUCCCCUGUGAGGAACUACUGUCUUCACGCAGAAAGCGUCUAGCCAUGGCGUUAGUAUGAGUGUCGUGCAGCCUCCAGGAC
233. DQ835764.1/1-117 GCCAGCCCCU-A-AU----GGGG-CGACACUCCACCAUG-AUCACUCCCCUGUGAGGAACUACUGUCUUCACGCAGAAAGCGUCUAGCCAUGGCGUUAGUAUGAGUGUCGUGCAGCCUCCAGGAC
234. DQ835762.1/1-117 GCCAGCCCCU-A-AU----GGGG-CGACACUCCACCAUG-AUCACUCCCCUGUGAGGAACUACUGUCUUCACGCAGAAAGCGUCUAGCCAUGGCGUUAGUAUGAGUGUCGUGCAGCCUCCAGGAC
235. DQ835761.1/1-117 GCCAGCCCCU-A-AU----GGGG-CGACACUCCACCAUG-AUCACUCCCCUGUGAGGAACUACUGUCUUCACGCAGAAAGCGUCUAGCCAUGGCGUUAGUAUGAGUGUCGUGCAGCCUCCAGGAC
236. DQ835760.1/1-117 GCCAGCCCCU-A-AU----GGGG-CGACACUCCACCAUG-AUCACUCCCCUGUGAGGAACUACUGUCUUCACGCAGAAAGCGUCUAGCCAUGGCGUUAGUAUGAGUGUCGUGCAGCCUCCAGGAC
237. KM504123.1/1-117 GCCAGCCCCU-A-AU----GGGG-CGACACUCCACCAUG-AUCACUCCCCUGUGAGGAACUACUGUCUUCACGCAGAAAGCGUCUAGCCAUGGCGUUAGUAUGAGUGUCGUGCAGCCUCCAGGAC
238. KM504119.1/1-117 GCCAGCCCCU-A-AU----GGGG-CGACACUCCACCAUG-AUCACUCCCCUGUGAGGAACUACUGUCUUCACGCAGAAAGCGUCUAGCCAUGGCGUUAGUAUGAGUGUCGUGCAGCCUCCAGGAC
239. KM504117.1/1-117 GCCAGCCCCU-A-AU----GGGG-CGACACUCCACCAUG-AUCACUCCCCUGUGAGGAACUACUGUCUUCACGCAGAAAGCGUCUAGCCAUGGCGUUAGUAUGAGUGUCGUGCAGCCUCCAGGAC
240. KM504115.1/1-117 GCCAGCCCCU-A-AU----GGGG-CGACACUCCACCAUG-AUCACUCCCCUGUGAGGAACUACUGUCUUCACGCAGAAAGCGUCUAGCCAUGGCGUUAGUAUGAGUGUCGUGCAGCCUCCAGGAC
241. KM504114.1/1-117 GCCAGCCCCU-A-AU----GGGG-CGACACUCCACCAUG-AUCACUCCCCUGUGAGGAACUACUGUCUUCACGCAGAAAGCGUCUAGCCAUGGCGUUAGUAUGAGUGUCGUGCAGCCUCCAGGAC
242. KM504110.1/1-117 GCCAGCCCCU-A-AU----GGGG-CGACACUCCACCAUG-AUCACUCCCCUGUGAGGAACUACUGUCUUCACGCAGAAAGCGUCUAGCCAUGGCGUUAGUAUGAGUGUCGUGCAGCCUCCAGGAC
243. JX183556.1/1-117 GCCAGCCCCU-A-AU----GGGG-CGACACUCCACCAUG-AUCACUCCCCUGUGAGGAACUACUGUCUUCACGCAGAAAGCGUCUAGCCAUGGCGUUAGUAUGAGUGUCGUGCAGCCUCCAGGAC
244. JX183554.1/1-117 GCCAGCCCCU-A-AU----GGGG-CGACACUCCACCAUG-AUCACUCCCCUGUGAGGAACUACUGUCUUCACGCAGAAAGCGUCUAGCCAUGGCGUUAGUAUGAGUGUCGUGCAGCCUCCAGGAC
245. JX183553.1/1-117 GCCAGCCCCU-A-AU----GGGG-CGACACUCCACCAUG-AUCACUCCCCUGUGAGGAACUACUGUCUUCACGCAGAAAGCGUCUAGCCAUGGCGUUAGUAUGAGUGUCGUGCAGCCUCCAGGAC
246. D84265.2/1-117 GCCAGCCCCU-A-AU----GGGG-CGACACUCCACCAUG-AUCACUCCCCUGUGAGGAACUACUGUCUUCACGCAGAAAGCGUCUAGCCAUGGCGUUAGUAUGAGUGUCGUGCAGCCUCCAGGAC
247. D84264.2/1-117 GCCAGCCCCU-A-AU----GGGG-CGACACUCCACCAUG-AUCACUCCCCUGUGAGGAACUACUGUCUUCACGCAGAAAGCGUCUAGCCAUGGCGUUAGUAUGAGUGUCGUGCAGCCUCCAGGAC
248. D84263.2/1-117 GCCAGCCCCU-A-AU----GGGG-CGACACUCCACCAUG-AUCACUCCCCUGUGAGGAACUACUGUCUUCACGCAGAAAGCGUCUAGCCAUGGCGUUAGUAUGAGUGUCGUGCAGCCUCCAGGAC
249. KM587630.1/1-117 GCCAGCCCCU-A-AU----GGGG-CGACACUCCACCAUG-AUCACUCCCCUGUGAGGAACUACUGUCUUCACGCAGAAAGCGUCUAGCCAUGGCGUUAGUAUGAGUGUCGUGCAGCCUCCAGGAC
250. EU408329.1/1-117 GCCAGCCCCU-A-AU----GGGG-CGACACUCCACCAUG-AUCACUCCCCUGUGAGGAACUACUGUCUUCACGCAGAAAGCGUCUAGCCAUGGCGUUAGUAUGAGUGUCGUGCAGCCUCCAGGAC
251. KU180720.1/1-120 GCCGGCUCCCUG-AU----GGGGgCGACACUCCACCAUGAAUCACUCCCCUGUGAGGAACUACUGUCUUCACGCAGAAAGCGUCUAGCCAUGGCGUUAGUAUGAGUGUCGUGCAGCCUCCAGGAC
252. KY014622.1/1-118 GCCAGCCCCUUA-AU----GGGG-CGACACUCCACCAUG-AUCACUCCCCUGUGAGGAACUAAUGUCUUCACGCAGAAAGCGUCUAGCCAUGGCGUUAGUAUGAGUGUCGUACAGCCUCCAGGAU
253. DQ314805.1/1-117 GCCAGCCCCU-A-AU----GGGG-CGACACUCCACCAUG-AUCACUCCCCUGUGAGGAACUACUGUCUUCACGCAGAAAGCGUCUAGCCAUGGCGUUAGUAUGAGUGUCGUGCAGCCUCCAGGAY
254. KJ678811.1/1-118 GCCAGCCCCUUA-AC----GGGG-SGACACUCCACCAUU-AUCACUCCCCUGUGAGGAACUACUGUCUUCACGCAGAAAGCGUCUAGCCAUGGCGUUAGUAUGAGUGUCGUACAGCCUCCAGGCC
255. JX183552.1/1-117 GCCAGCCCCU-A-AU----GGGG-CGACACUCCACCAUA-AUCACUCCCCUGUGAGGAACUACUGUCUUCACGCAGAAAGCGUCUAGCCAUGGCGUUAGUAUGAGUGUYGUGCAGCCUCCAGGAC
256. KJ678757.1/1-117 GCCAGCCCCU-A-AU----GGGG-CGACACUCCACCAUU-AUCACUCCCCUGUGAGGAACUACUGUCUUCACGCAGAAAGCGUCUAGCCAUGGCGUUAGUAUGAGUGUCGUGCAGCCUCCAGGAC
257. JX183551.1/1-117 GCCAGCCCCU-A-AU----GGGG-CGACACUCCACCAUU-AUCACUCCCCUGUGAGGAACUACUGUCUUCACGCAGAAAGCGUCUAGCCAUGGCGUUAGUAUGAGUGUCGUGCAGCCUCCAGGAC
258. JX183555.1/1-117 GCCAGCCCCU-A-AU----GGGG-CGACACUCCACCAUG-AUCACUCCCCUGUGAGGAACUACUGUCUUCACGCAGAAAGCGUCUAGCCAUGGCGUUAGUAUGAGUGUYGUGCAGCCUCCAGGAC
259. DQ278893.1/4-120 GCCAGCCCCU-A-AU----GGGG-CGACACUCCACCAUA-AUCACUCCCCUGUGAGGAACUACUGUCUUCACGCAGAAAGCGUCUAGCCAUGGCGUUAGUAUGAGUGUCGUGCAGCCUCCAGGAU
260. GU294484.1/1-118 ACCUGCCUCUUA--C----GAGG-CGACACUCCACCAUGGAUCACUCCCCUGUGAGGAACUACUGUCUUCACGCAGAAAGCGUCUAGCCAUGGCGUUAGUAUGAGUGUCGUGCAGCCUCCAGGAC
261. DQ278891.1/3-119 GCCAGCCCCU-A-AU----GGGG-CGACACUCCACCAUG-AUCACUCCCCUGUGAGGAACUACUGUCUUCACGCAGAAAGCGUCUAGCCAUGGCGUUAGUAUGAGUGUCGUGCAGCCUCCAGGAU
262. FJ435090.1/1-117 GCCAGCCCCU-A-AU----GGGG-CGACACUCCACCAUG-AUCACUCCCCUGUGAGGAACUACUGUCUUCACGCAGAAAGCGUCUAGCCAUGGCGUUAGUAUGAGUGUCGUGCAGCCUCCAGGAU
263. EF424626.1/1-117 GCCAGCCCCU-A-AU----GGGG-CGACACUCCACCAUG-AUCACUCCCCUGUGAGGAACUACUGUCUUCACGCAGAAAGCGUCUAGCCAUGGCGUUAGUAUGAGUGUCGUGCAGCCUCCAGGAU
264. EF632069.1/1-117 GCCAGCCCCU-A-AU----GGGG-CGACACUCCACCAUG-AUCACUCCCCUGUGAGGAACUACUGUCUUCACGCAGAAAGCGUCUAGCCAUGGCGUUAGUAUGAGUGUCGUGCAGCCUCCAGGAU
265. DQ835763.1/1-117 GCCAGCCCCU-A-AU----GGGG-CGACACUCCACCAUG-AUCACUCCCCUGUGAGGAACUACUGUCUUCACGCAGAAAGCGUCUAGCCAUGGCGUUAGUAUGAGUGUCGUGCAGCCUCCAGGAU
266. KM504121.1/1-117 GCCAGCCCCU-A-AU----GGGG-CGACACUCCACCAUG-AUCACUCCCCUGUGAGGAACUACUGUCUUCACGCAGAAAGCGUCUAGCCAUGGCGUUAGUAUGAGUGUCGUGCAGCCUCCAGGAU
267. KM504120.1/1-117 GCCAGCCCCU-A-AU----GGGG-CGACACUCCACCAUG-AUCACUCCCCUGUGAGGAACUACUGUCUUCACGCAGAAAGCGUCUAGCCAUGGCGUUAGUAUGAGUGUCGUGCAGCCUCCAGGAU
268. JX183549.1/1-117 GCCAGCCCCU-A-AU----GGGG-CGACACUCCACCAUG-AUCACUCCCCUGUGAGGAACUACUGUCUUCACGCAGAAAGCGUCUAGCCAUGGCGUUAGUAUGAGUGUCGUGCAGCCUCCAGGAU
269. EU798760.1/1-117 GCCAGCCCCU-A-AU----GGGG-CGACACUCCACCAUG-AUCACUCCCCUGUGAGGAACUACUGUCUUCACGCAGAAAGCGUCUAGCCAUGGCGUUAGUAUGAGUGUCGUGCAGCCUCCAGGAU
270. KM504124.1/1-117 GCCAGCCCCU-A-AU----GGGG-CGACACUCCACCAUG-AUCACUCCCCUGUGAGGAACUACUGUCUUCACGCAGAAAGCGUCUAGCCAUGGCGUUAGUAUGAGUGUCGUACAGCCUCCAGGCC
271. KM504118.1/1-117 GCCAGCCCCU-A-AU----GGGG-CGACACUCCACCAUG-AUCACUCCCCUGUGAGGAACUACUGUCUUCACGCAGAAAGCGUCUAGCCAUGGCGUUAGUAUGAGUGUCGUACAGCCUCCAGGCC
272. KM587629.1/1-117 GCCAGCCCCU-A-AC----GGGG-CGACACUCCACCAUG-AUCACUCCCCUGUGAGGAACUACUGUCUUCACGCAGAAAGCGUCUAGCCAUGGCGUUAGUAUGAGUGUCGUGCAGCCUCCAGGAC
273. KM504111.1/1-117 GCCAGCCCCU-A-AU----GGGG-CGACACUCCACCAUG-AUCACUCCCCUGUGAGGAACUACUGUCUUCACGCAGAAAGCGUCUAGCCAUGGCGUUAGUAUGAGUGUCGUGCAGCCUCCAGGCC
274. KM504112.1/1-117 GCCAGCCCCU-A-AU----GGGG-CGACACUCCACCAUR-AUCACUCCCCUGUGAGGAACUACUGUCUUCACGCAGAAAGCGUCUAGCCAUGGCGUUAGUAUGAGUGUUGUGCAGCCUCCAGGAC
275. D63822.1/1-117 GCCAGCCCCUUA--C----GGGG-CGACACUCCACCAUG-AUCACUCCCCUGUGAGGAACUACUGUCUUCACGCAGAAAGCGUCUAGCCAUGGCGUUAGUAUGAGUGUCGUGCAGCCUCCAGGAC
276. EU408328.1/1-117 GCCAGCCCCU-A-AU----GGGG-CGACACUCCGCCAUA-AUCACUCCCCUGUGAGGAACUACUGUCUUCACGCAGAAAGCGUCUAGCCAUGGCGUUAGUAUGAGUGUCGUGCAGCCUCCAGGAC
277. KY620347.1/17-135 GCCCUCCCCUGA-UG----GGGG-CGACACUCCGCCAUGAAUCACUCCCCUGUGAGGAACUACUGUCUUCACGCAGAAAGCGUCUAGCCAUGGCGUUAGUAUGAGUGUCGUACAGCCUCCAGGCC
278. KJ567646.1/1-117 GCCAGCCCCU-A-AU----GGGG-CGACACUCCACCAUG-AUCACUCCCCUGUGAGGAACUACUGUCUUCACGCAGAAAGCGUYUAGCCAUGGCGUUAGUAUGAGUGUCGUGCAGCCUCCAGGAC
279. JF735134.1/1-119 ACCUGCUCUCUA-UG----AGAG-CAACACUCCGCCAUGAAUCACUCCCCUGUGAGGAACUACUGUCUUCACGCAGAAAGCGUCUAGCCAUGGCGUUAGUAUGAGUGUUGUACAGCCUCCAGGMC
280. KJ678807.1/1-117 GCCAGCCCCU-A-AU----GGGG-CGACACUCCACCAUU-AUCACUCCCCUGUGAGGAACUACUGUCUUCACGCAGAAAGCGUCUAGCCAUGGCGUUAGUAUGAGUGUCGUACAGCCUCCAGGCC
281. KJ678802.1/1-117 GCCAGCCCCU-A-AU----GGGG-CGACACUCCACCAUU-AUCACUCCCCUGUGAGGAACUACUGUCUUCACGCAGAAAGCGUCUAGCCAUGGCGUUAGUAUGAGUGUCGUACAGCCUCCAGGCC
282. KJ678789.1/1-117 GCCAGCCCCU-A-AU----GGGG-CGACACUCCACCAUU-AUCACUCCCCUGUGAGGAACUACUGUCUUCACGCAGAAAGCGUCUAGCCAUGGCGUUAGUAUGAGUGUCGUACAGCCUCCAGGCC
283. KJ678764.1/1-117 GCCAGCCCCU-A-AU----GGGG-CGACACUCCACCAUU-AUCACUCCCCUGUGAGGAACUACUGUCUUCACGCAGAAAGCGUCUAGCCAUGGCGUUAGUAUGAGUGUCGUACAGCCUCCAGGCC
284. KJ678760.1/1-117 GCCAGCCCCU-A-AU----GGGG-CGACACUCCACCAUU-AUCACUCCCCUGUGAGGAACUACUGUCUUCACGCAGAAAGCGUCUAGCCAUGGCGUUAGUAUGAGUGUCGUACAGCCUCCAGGCC
285. KJ678759.1/1-117 GCCAGCCCCU-A-AU----GGGG-CGACACUCCACCAUU-AUCACUCCCCUGUGAGGAACUACUGUCUUCACGCAGAAAGCGUCUAGCCAUGGCGUUAGUAUGAGUGUCGUACAGCCUCCAGGCC
286. KJ678758.1/1-117 GCCAGCCCCU-A-AU----GGGG-CGACACUCCACCAUU-AUCACUCCCCUGUGAGGAACUACUGUCUUCACGCAGAAAGCGUCUAGCCAUGGCGUUAGUAUGAGUGUCGUACAGCCUCCAGGCC
287. KJ678756.1/1-117 GCCAGCCCCU-A-AU----GGGG-CGACACUCCACCAUU-AUCACUCCCCUGUGAGGAACUACUGUCUUCACGCAGAAAGCGUCUAGCCAUGGCGUUAGUAUGAGUGUCGUACAGCCUCCAGGCC
288. KM504122.1/1-117 GCCAGCCCCU-A-AU----GGGG-CGACACUCCACCAUG-AUCACUCCCCUGUGAGGAACUACUGUCUUCACGCAGAAAGCGUCUAGCCACGGCGUUAGUAUGAGUGUCGUGCAGCCUCCAGGAC
289. KJ678788.1/1-117 GCCAGCCCCU-A-AU----GGGG-CGACACUCCACCAUU-AUCACUCCCCUGUGAGGAACUACUGUCUUCACGCAGAAAGCGUCUAGCCAUGGCGUUAGUAUGAGUGUCGURCAGCCUCCAGGCC
290. KJ678804.1/1-117 GCCAGCCCCU-A-AU----GGGG-CGACACUCCACCAUU-AUCACUCCCCUGUGAGGAACUACUGUCUUCACGCAGAAAGCGUCUAGCCAUGGCGUUAGUAUGAGUGUCGUGCAGCCUCCAGGCC
291. AF356827.1/1-120 GCCAGCCCCCGA-UU----GGGGgCGACACUCCACCAUAGAUCACUCCCCUGUGAGGAACUACUGUCUUCACGCAGAAAGCGUCUAGCCAUGGCGUUAGUAUGAGUGUCGUGUAGCCUCCGGGAC
292. DQ071885.1/1-120 GCCAGCCCCCGA-UU----GGGGgCGACACUCCACCAUAGAUCACUCCCCUGUGAGGAACUACUGUCUUCACGCAGAAAGCGUCUAGCCAUGGCGUUAGUAUGAGUGUCGUGCAGCCUCCAUGAC
293. EF424629.1/1-117 GCCAGCCCCU-A-AU----GGGG-CGACACUCCACCAUG-AUCACUCCCCUGUGAGGAACUACUGUCUUCACGCAGAAAGCGUCUAGCCAUGGCGUUAGUAUGAGUGUUGUACAGCCUCCAGGCC
294. KJ678761.1/1-117 GCCAGCCCCU-A-AU----GGGG-CGACACUCCACCAUC-AUCACUCCCCUGUGAGGAACUACUGUCUUCACGCAGAAAGCGUCUAGCCAUGGCGUUAGUAUGAGUGUCGUGCAGCCUCCAGGCC
295. KY620330.1/1-118 -CCCGCCCCUAA-AA----GGGG-CGACACUCCGCCAUGAAUCACUCCCCUGUGAGGAACUACUGUCUUCACGCAGAAAGCGUCUAGCCAUGGCGUUAGUAUGAGUGUCGUACAGCCUCCAGGCC
296. KM587619.1/1-118 -CCCGCCCCUAA-AA----GGGG-CGACACUCCGCCAUGAAUCACUCCCCUGUGAGGAACUACUGUCUUCACGCAGAAAGCGUCUAGCCAUGGCGUUAGUAUGAGUGUCGUACAGCCUCCAGGCC
297. DQ314806.1/1-117 GCCAGCCCCU-A-AU----GGGG-CGACACUCCACCAUG-AUCACUCCCCUGUGAGGAGCUACUGUCUUCACGCAGAAAGCGUCUAGCCAUGGCGUUAGUAUGAGUGUCGUGCAGCCUCCAGGAC
298. KJ567650.1/1-117 GCCAGCCCCU-A-AU----GGGG-CGACACUCCACCAUG-AUCACUCCCCUGUGAGGAACUACUGUCUUCACGCAGGAAGCGUCUAGCCAUGGCGUUAGUAUGAGUGUCGUGCAGCCUCCAGGAC
299. KU180731.1/1-119 CCAGCCCCCCAU-UG----GGGG-CGACACUCCACCAUGAAUCACUCCCCUGUGAGGAACUACUGUCUUCACGCAGAAAGCGUCUAGCCAUGGCGUUAGUAUGAGUGUCGUGCAGCCUCCAGGAC
300. KY620842.1/21-138 ACCUGCCUCU-A-AU----GAGG-CGACACUCCACCAUGGAUCACUCCCCUGUGAGGAACUUCUGUCUUCACGCGGAAAGCGCCUAGCCAUGGCGUUAGUACGAGUGUCGUGCAGCCUCCAGGAC
301. KY620336.1/27-145 GCUCUCCCCUGA-UG----GGGG-CGACACUCCGCCAUGAAUCACUCCCCUGUGAGGAACUACUGUCUUCACGCAGAAAGCGUCUAGCCAUGGCGUUAGUAUGAGUGUCGUACAGCCUCCAGGCC
302. KY620833.1/16-133 ACCUGCCUCUUA--U----GAGG-CGACACUCCACCAUGGAUCACUCCCCUGUGAGGAACUUCUGUCUUCACGCGGAAAGCGCCUAGCCAUGGCGUUAGUACGAGUGUCGUGCAGCCUCCAGGAC
303. KY620740.1/6-123 ACCUGCCUCUUA--U----GAGG-CGACACUCCACCAUGGAUCACUCCCCUGUGAGGAACUUCUGUCUUCACGCGGAAAGCGCCUAGCCAUGGCGUUAGUACGAGUGUCGUGCAGCCUCCAGGAC
304. KY620729.1/21-138 ACCUGCCUCUUA--U----GAGG-CGACACUCCACCAUGGAUCACUCCCCUGUGAGGAACUUCUGUCUUCACGCGGAAAGCGCCUAGCCAUGGCGUUAGUACGAGUGUCGUGCAGCCUCCAGGAC
305. KY620720.1/21-138 ACCUGCCUCUUA--U----GAGG-CGACACUCCACCAUGGAUCACUCCCCUGUGAGGAACUUCUGUCUUCACGCGGAAAGCGCCUAGCCAUGGCGUUAGUACGAGUGUCGUGCAGCCUCCAGGAC
306. KY620536.1/25-142 ACCUGCCUCUUA--U----GAGG-CGACACUCCACCAUGGAUCACUCCCCUGUGAGGAACUUCUGUCUUCACGCGGAAAGCGCCUAGCCAUGGCGUUAGUACGAGUGUCGUGCAGCCUCCAGGAC
307. KY620472.1/21-138 ACCUGCCUCUUA--U----GAGG-CGACACUCCACCAUGGAUCACUCCCCUGUGAGGAACUUCUGUCUUCACGCGGAAAGCGCCUAGCCAUGGCGUUAGUACGAGUGUCGUGCAGCCUCCAGGAC
308. KY620349.1/5-124 CCCCGCUCCCUG-AU----GGGGgCGACACUCCGCCAUGAAUCACUCCCCUGUGAGGAACUACUGUCUUCACGCAGAAAGCGUCUAGCCAUGGCGUUAGUAUGAGUGUCGUGCAGCCUCCAGGCC
309. KM504116.1/1-117 GCCAGCCCCU-A-AU----GGGG-CGACACUCCACCAUG-AUCACUCCCCUGUGAGGAACUACUGUCUUCACGCAGAAAGCGUCUAACCAUGGCGUUAGUAUGAGUGUUGUGCAGCCUCCAGGAC
310. JX183557.1/1-117 GCCAGCCCCU-A-AU----GGGG-SGACACUCCACCAUG-AUCACUCCCCUGUGAGGAACUACUGUCUUCACGCAGAAAGCGUCUAGCCAUGGCGUUAGUAUGAGUGUCGUACAGCCUCCAGGAU
311. KU180727.1/1-120 GAAAGCACCCUG-AU---gGGGG-CGACACUCCACCAUGAAUCACUCCCCUGUGAGGAACUACUGUCUUCACGCAGAAAGCGUCUAGCCAUGGCGUUAGUAUGAGUGUCGUGCAGCCUCCAGGAC
312. KY620564.1/13-130 ACCUGCCUCUUA--C----GAGG-CGACACUCCACCAUGAAUCACUCCCCUGUGAGGAACUUCUGUCUUCACGCGGAAAGCGUCUAGCCAUGGCGUUAGUACGAGUGUCGUGCAGCCUCCAGGAC
313. KC248194.1/1-119 GCCAGCCCCCUG-AU----GGGGgCGACAC-CCACCAUAGAUCACUCCCCUGUGAGGAACUACUGUCUUCACGCAGAAAGCGUCUAGCCAUGGCGUUAGUAUGAGUGUCGUGCAGCCUCCAGGAC
314. KY620693.1/23-140 ACCUGCCUCCUA-C-----GAGG-CGACACUCCACCAUGGAUCACUCCCCUGUGAGGAACUUCUGUCUUCACGCGGAAAGCGCCUAGCCAUGGCGUUAGUACGAGUGUCGUGCAGCCUCCAGGAC
315. JF343784.2/1-118 GCCUGCCUCUUA--C----GAGG-CGACACUCCACCAUGGAUCACUCCCCUGUGAGGAACUUCUGUCUUCACGCGGAAAGCGCCUAGCCAUGGCGUUAGUACGAGUGUCGUGCAGCCUCCAGGAC
316. D28917.1/1-118 GCCUGCCUCUUA--C----GAGG-CGACACUCCACCAUGGAUCACUCCCCUGUGAGGAACUUCUGUCUUCACGCGGAAAGCGCCUAGCCAUGGCGUUAGUACGAGUGUCGUGCAGCCUCCAGGAC
317. KY620582.1/22-139 GCCUGCCUCUUA--C----GAGG-CGACACUCCACCAUGGAUCACUCCCCUGUGAGGAACUUCUGUCUUCACGCGGAAAGCGCCUAGCCAUGGCGUUAGUACGAGUGUCGUGCAGCCUCCAGGAC
318. KY620732.1/7-124 ACCUGCCUCCUA-G-----GAGG-CGACACUCCACCAUGGAUCACUCCCCUGUGAGGAACUUCUGUCUUCACGCGGAAAGCGCCUAGCCAUGGCGUUAGUACGAGUGUCGUGCAGCCUCCAGGAC
319. KY620728.1/22-139 ACCUGCCUCUUA--C----GAGG-CGACACUCCACCAUAGAUCACUCCCCUGUGAGGAACUUCUGUCUUCACGCGGAAAGCGUCUAGCCAUGGCGUUAGUACGAGUGUCGUGCAGCCUCCAGGAC
320. KY620859.1/17-134 ACCUGCCUCUUA--C----GAGG-CGACACUCCACCAUGGAUCACUCCCCUGUGAGGAACUUCUGUCUUCACGCGGAAAGCGUCUAGCCAUGGCGUUAGUACGAGUGUCGUGCAGCCUCCAGGAC
321. KY620826.1/17-134 ACCUGCCUCUUA--C----GAGG-CGACACUCCACCAUGGAUCACUCCCCUGUGAGGAACUUCUGUCUUCACGCGGAAAGCGUCUAGCCAUGGCGUUAGUACGAGUGUCGUGCAGCCUCCAGGAC
322. KJ678763.1/1-117 GCCAGCCCCU-A-AU----GGGG-CGACACUCCACCAUU-GUCACUCCCCUGUGAGGAACUACUGUCUUCACGCAGAAAGCGUCUAGCCAUGGCGUUAGUAUGAGUGUCGUACAGCCUCCAGGCC
323. KJ678762.1/1-117 GCCAGCCCCU-A-AU----GGGG-CGACACUCCACCAUU-GUCACUCCCCUGUGAGGAACUACUGUCUUCACGCAGAAAGCGUCUAGCCAUGGCGUUAGUAUGAGUGUCGUACAGCCUCCAGGCC
324. KY620783.1/18-135 ACCUGCCUCUUA--C----GAGG-CGACACUCCACCAUAGAUCACUCCCCUGUGAGGAACUUCUGUCUUCACGCGGAAAGCGCCUAGCCAUGGCGUUAGUACGAGUGUCGUGCAGCCUCCAGGAC
325. JF735137.1/1-119 ACCUGCUCUCUA-UG----AGAG-CAACACUCCACCAUGAACCGCUCCCCUGUGAGGAACUACUGUCUUCACGCAGAAAGCGUCUAGCCAUGGCGUUAGUAUGAGUGUUGUACAGCCUCCAGGAC
326. JF735135.1/1-119 ACCUGCUCUCUA-UG----AGAG-CAACACUCCACCAUGAACCGCUCCCCUGUGAGGAACUACUGUCUUCACGCAGAAAGCGUCUAGCCAUGGCGUUAGUAUGAGUGUUGUACAGCCUCCAGGAC
327. JF735133.1/1-119 ACCUGCUCUCUA-UG----AGAG-CAACACUCCACCAUGAACCGCUCCCCUGUGAGGAACUACUGUCUUCACGCAGAAAGCGUCUAGCCAUGGCGUUAGUAUGAGUGUUGUACAGCCUCCAGGAC
328. JF735128.1/1-119 ACCUGCUCUCUA-UG----AGAG-CAACACUCCACCAUGAACCGCUCCCCUGUGAGGAACUACUGUCUUCACGCAGAAAGCGUCUAGCCAUGGCGUUAGUAUGAGUGUUGUACAGCCUCCAGGAC
329. FJ839870.1/1-119 ACCUGCUCUCUA-UG----AGAG-CAACACUCCACCAUGAACCGCUCCCCUGUGAGGAACUACUGUCUUCACGCAGAAAGCGUCUAGCCAUGGCGUUAGUAUGAGUGUUGUACAGCCUCCAGGAC
330. FJ839869.1/1-119 ACCUGCUCUCUA-UG----AGAG-CAACACUCCACCAUGAACCGCUCCCCUGUGAGGAACUACUGUCUUCACGCAGAAAGCGUCUAGCCAUGGCGUUAGUAUGAGUGUUGUACAGCCUCCAGGAC
331. FJ462441.1/1-119 ACCUGCUCUCUA-UG----AGAG-CAACACUCCACCAUGAACCGCUCCCCUGUGAGGAACUACUGUCUUCACGCAGAAAGCGUCUAGCCAUGGCGUUAGUAUGAGUGUUGUACAGCCUCCAGGAC
332. FJ462440.1/1-119 ACCUGCUCUCUA-UG----AGAG-CAACACUCCACCAUGAACCGCUCCCCUGUGAGGAACUACUGUCUUCACGCAGAAAGCGUCUAGCCAUGGCGUUAGUAUGAGUGUUGUACAGCCUCCAGGAC
333. FJ462437.1/1-119 ACCUGCUCUCUA-UG----AGAG-CAACACUCCACCAUGAACCGCUCCCCUGUGAGGAACUACUGUCUUCACGCAGAAAGCGUCUAGCCAUGGCGUUAGUAUGAGUGUUGUACAGCCUCCAGGAC
334. FJ462436.1/1-119 ACCUGCUCUCUA-UG----AGAG-CAACACUCCACCAUGAACCGCUCCCCUGUGAGGAACUACUGUCUUCACGCAGAAAGCGUCUAGCCAUGGCGUUAGUAUGAGUGUUGUACAGCCUCCAGGAC
335. FJ462435.1/1-119 ACCUGCUCUCUA-UG----AGAG-CAACACUCCACCAUGAACCGCUCCCCUGUGAGGAACUACUGUCUUCACGCAGAAAGCGUCUAGCCAUGGCGUUAGUAUGAGUGUUGUACAGCCUCCAGGAC
336. FJ462431.1/1-119 ACCUGCUCUCUA-UG----AGAG-CAACACUCCACCAUGAACCGCUCCCCUGUGAGGAACUACUGUCUUCACGCAGAAAGCGUCUAGCCAUGGCGUUAGUAUGAGUGUUGUACAGCCUCCAGGAC
337. JF343785.2/1-119 ACCUGCUCUCUA-UG----AGAG-CAACACUCCACCAUGAACCGCUCCCCUGUGAGGAACUACUGUCUUCACGCAGAAAGCGUCUAGCCAUGGCGUUAGUAUGAGUGUUGUACAGCCUCCAGGAC
338. KM587625.1/1-119 ACCUGCUCUCUA-UG----AGAG-CAACACUCCACCAUGAACCGCUCCCCUGUGAGGAACUACUGUCUUCACGCAGAAAGCGUCUAGCCAUGGCGUUAGUAUGAGUGUUGUACAGCCUCCAGGAC
339. KJ567651.1/1-117 GCCAGCCCCU-A-AU----GGGG-SGACAYUCCACCAUG-AUCAYUCCCCUGUGAGGAACUACUGUCUUCACGCAGAAAGCGUCUAGCCAUGGCGUUAGUAUGAGUGUCGUACAGCCUCCAGGAC
340. GQ275355.1/1-118 ACCUGCCUCUUA--C----GAGG-CGACACUCCACCAUGGAUCACUCCCCUGUGAGGAACUUCUGUCUUCACGCGGAAAGCGCCUAGCCAUGGCGUUAGUACGAGUGUCGUGCAGCCUCCAGGAC
341. JQ717260.1/1-118 ACCUGCCUCUUA--C----GAGG-CGACACUCCACCAUGGAUCACUCCCCUGUGAGGAACUUCUGUCUUCACGCGGAAAGCGCCUAGCCAUGGCGUUAGUACGAGUGUCGUGCAGCCUCCAGGAC
342. JQ717257.1/1-118 ACCUGCCUCUUA--C----GAGG-CGACACUCCACCAUGGAUCACUCCCCUGUGAGGAACUUCUGUCUUCACGCGGAAAGCGCCUAGCCAUGGCGUUAGUACGAGUGUCGUGCAGCCUCCAGGAC
343. JQ717255.1/1-118 ACCUGCCUCUUA--C----GAGG-CGACACUCCACCAUGGAUCACUCCCCUGUGAGGAACUUCUGUCUUCACGCGGAAAGCGCCUAGCCAUGGCGUUAGUACGAGUGUCGUGCAGCCUCCAGGAC
344. JQ717254.1/1-118 ACCUGCCUCUUA--C----GAGG-CGACACUCCACCAUGGAUCACUCCCCUGUGAGGAACUUCUGUCUUCACGCGGAAAGCGCCUAGCCAUGGCGUUAGUACGAGUGUCGUGCAGCCUCCAGGAC
345. AB792683.1/1-118 ACCUGCCUCUUA--C----GAGG-CGACACUCCACCAUGGAUCACUCCCCUGUGAGGAACUUCUGUCUUCACGCGGAAAGCGCCUAGCCAUGGCGUUAGUACGAGUGUCGUGCAGCCUCCAGGAC
346. AB691596.1/1-118 ACCUGCCUCUUA--C----GAGG-CGACACUCCACCAUGGAUCACUCCCCUGUGAGGAACUUCUGUCUUCACGCGGAAAGCGCCUAGCCAUGGCGUUAGUACGAGUGUCGUGCAGCCUCCAGGAC
347. AB691595.1/1-118 ACCUGCCUCUUA--C----GAGG-CGACACUCCACCAUGGAUCACUCCCCUGUGAGGAACUUCUGUCUUCACGCGGAAAGCGCCUAGCCAUGGCGUUAGUACGAGUGUCGUGCAGCCUCCAGGAC
348. D17763.1/1-118 ACCUGCCUCUUA--C----GAGG-CGACACUCCACCAUGGAUCACUCCCCUGUGAGGAACUUCUGUCUUCACGCGGAAAGCGCCUAGCCAUGGCGUUAGUACGAGUGUCGUGCAGCCUCCAGGAC
349. KY620844.1/14-131 ACCUGCCUCUUA--C----GAGG-CGACACUCCACCAUGGAUCACUCCCCUGUGAGGAACUUCUGUCUUCACGCGGAAAGCGCCUAGCCAUGGCGUUAGUACGAGUGUCGUGCAGCCUCCAGGAC
350. KY620843.1/14-131 ACCUGCCUCUUA--C----GAGG-CGACACUCCACCAUGGAUCACUCCCCUGUGAGGAACUUCUGUCUUCACGCGGAAAGCGCCUAGCCAUGGCGUUAGUACGAGUGUCGUGCAGCCUCCAGGAC
351. KY620840.1/6-123 ACCUGCCUCUUA--C----GAGG-CGACACUCCACCAUGGAUCACUCCCCUGUGAGGAACUUCUGUCUUCACGCGGAAAGCGCCUAGCCAUGGCGUUAGUACGAGUGUCGUGCAGCCUCCAGGAC
352. KY620827.1/13-130 ACCUGCCUCUUA--C----GAGG-CGACACUCCACCAUGGAUCACUCCCCUGUGAGGAACUUCUGUCUUCACGCGGAAAGCGCCUAGCCAUGGCGUUAGUACGAGUGUCGUGCAGCCUCCAGGAC
353. KY620798.1/12-129 ACCUGCCUCUUA--C----GAGG-CGACACUCCACCAUGGAUCACUCCCCUGUGAGGAACUUCUGUCUUCACGCGGAAAGCGCCUAGCCAUGGCGUUAGUACGAGUGUCGUGCAGCCUCCAGGAC
354. KY620791.1/17-134 ACCUGCCUCUUA--C----GAGG-CGACACUCCACCAUGGAUCACUCCCCUGUGAGGAACUUCUGUCUUCACGCGGAAAGCGCCUAGCCAUGGCGUUAGUACGAGUGUCGUGCAGCCUCCAGGAC
355. KY620780.1/22-139 ACCUGCCUCUUA--C----GAGG-CGACACUCCACCAUGGAUCACUCCCCUGUGAGGAACUUCUGUCUUCACGCGGAAAGCGCCUAGCCAUGGCGUUAGUACGAGUGUCGUGCAGCCUCCAGGAC
356. KY620708.1/10-127 ACCUGCCUCUUA--C----GAGG-CGACACUCCACCAUGGAUCACUCCCCUGUGAGGAACUUCUGUCUUCACGCGGAAAGCGCCUAGCCAUGGCGUUAGUACGAGUGUCGUGCAGCCUCCAGGAC
357. KY620695.1/23-140 ACCUGCCUCUUA--C----GAGG-CGACACUCCACCAUGGAUCACUCCCCUGUGAGGAACUUCUGUCUUCACGCGGAAAGCGCCUAGCCAUGGCGUUAGUACGAGUGUCGUGCAGCCUCCAGGAC
358. KY620667.1/20-137 ACCUGCCUCUUA--C----GAGG-CGACACUCCACCAUGGAUCACUCCCCUGUGAGGAACUUCUGUCUUCACGCGGAAAGCGCCUAGCCAUGGCGUUAGUACGAGUGUCGUGCAGCCUCCAGGAC
359. KY620650.1/14-131 ACCUGCCUCUUA--C----GAGG-CGACACUCCACCAUGGAUCACUCCCCUGUGAGGAACUUCUGUCUUCACGCGGAAAGCGCCUAGCCAUGGCGUUAGUACGAGUGUCGUGCAGCCUCCAGGAC
360. KY620641.1/13-130 ACCUGCCUCUUA--C----GAGG-CGACACUCCACCAUGGAUCACUCCCCUGUGAGGAACUUCUGUCUUCACGCGGAAAGCGCCUAGCCAUGGCGUUAGUACGAGUGUCGUGCAGCCUCCAGGAC
361. KY620633.1/13-130 ACCUGCCUCUUA--C----GAGG-CGACACUCCACCAUGGAUCACUCCCCUGUGAGGAACUUCUGUCUUCACGCGGAAAGCGCCUAGCCAUGGCGUUAGUACGAGUGUCGUGCAGCCUCCAGGAC
362. KY620629.1/11-128 ACCUGCCUCUUA--C----GAGG-CGACACUCCACCAUGGAUCACUCCCCUGUGAGGAACUUCUGUCUUCACGCGGAAAGCGCCUAGCCAUGGCGUUAGUACGAGUGUCGUGCAGCCUCCAGGAC
363. KY620619.1/21-138 ACCUGCCUCUUA--C----GAGG-CGACACUCCACCAUGGAUCACUCCCCUGUGAGGAACUUCUGUCUUCACGCGGAAAGCGCCUAGCCAUGGCGUUAGUACGAGUGUCGUGCAGCCUCCAGGAC
364. KY620617.1/11-128 ACCUGCCUCUUA--C----GAGG-CGACACUCCACCAUGGAUCACUCCCCUGUGAGGAACUUCUGUCUUCACGCGGAAAGCGCCUAGCCAUGGCGUUAGUACGAGUGUCGUGCAGCCUCCAGGAC
365. KY620599.1/10-127 ACCUGCCUCUUA--C----GAGG-CGACACUCCACCAUGGAUCACUCCCCUGUGAGGAACUUCUGUCUUCACGCGGAAAGCGCCUAGCCAUGGCGUUAGUACGAGUGUCGUGCAGCCUCCAGGAC
366. KY620560.1/5-122 ACCUGCCUCUUA--C----GAGG-CGACACUCCACCAUGGAUCACUCCCCUGUGAGGAACUUCUGUCUUCACGCGGAAAGCGCCUAGCCAUGGCGUUAGUACGAGUGUCGUGCAGCCUCCAGGAC
367. KY620558.1/14-131 ACCUGCCUCUUA--C----GAGG-CGACACUCCACCAUGGAUCACUCCCCUGUGAGGAACUUCUGUCUUCACGCGGAAAGCGCCUAGCCAUGGCGUUAGUACGAGUGUCGUGCAGCCUCCAGGAC
368. KY620539.1/26-143 ACCUGCCUCUUA--C----GAGG-CGACACUCCACCAUGGAUCACUCCCCUGUGAGGAACUUCUGUCUUCACGCGGAAAGCGCCUAGCCAUGGCGUUAGUACGAGUGUCGUGCAGCCUCCAGGAC
369. KY620529.1/21-138 ACCUGCCUCUUA--C----GAGG-CGACACUCCACCAUGGAUCACUCCCCUGUGAGGAACUUCUGUCUUCACGCGGAAAGCGCCUAGCCAUGGCGUUAGUACGAGUGUCGUGCAGCCUCCAGGAC
370. KY620517.1/22-139 ACCUGCCUCUUA--C----GAGG-CGACACUCCACCAUGGAUCACUCCCCUGUGAGGAACUUCUGUCUUCACGCGGAAAGCGCCUAGCCAUGGCGUUAGUACGAGUGUCGUGCAGCCUCCAGGAC
371. KY620501.1/12-129 ACCUGCCUCUUA--C----GAGG-CGACACUCCACCAUGGAUCACUCCCCUGUGAGGAACUUCUGUCUUCACGCGGAAAGCGCCUAGCCAUGGCGUUAGUACGAGUGUCGUGCAGCCUCCAGGAC
372. KY620487.1/10-127 ACCUGCCUCUUA--C----GAGG-CGACACUCCACCAUGGAUCACUCCCCUGUGAGGAACUUCUGUCUUCACGCGGAAAGCGCCUAGCCAUGGCGUUAGUACGAGUGUCGUGCAGCCUCCAGGAC
373. KY620475.1/16-133 ACCUGCCUCUUA--C----GAGG-CGACACUCCACCAUGGAUCACUCCCCUGUGAGGAACUUCUGUCUUCACGCGGAAAGCGCCUAGCCAUGGCGUUAGUACGAGUGUCGUGCAGCCUCCAGGAC
374. KY620466.1/21-138 ACCUGCCUCUUA--C----GAGG-CGACACUCCACCAUGGAUCACUCCCCUGUGAGGAACUUCUGUCUUCACGCGGAAAGCGCCUAGCCAUGGCGUUAGUACGAGUGUCGUGCAGCCUCCAGGAC
375. KY620448.1/16-133 ACCUGCCUCUUA--C----GAGG-CGACACUCCACCAUGGAUCACUCCCCUGUGAGGAACUUCUGUCUUCACGCGGAAAGCGCCUAGCCAUGGCGUUAGUACGAGUGUCGUGCAGCCUCCAGGAC
376. KY620427.1/19-136 ACCUGCCUCUUA--C----GAGG-CGACACUCCACCAUGGAUCACUCCCCUGUGAGGAACUUCUGUCUUCACGCGGAAAGCGCCUAGCCAUGGCGUUAGUACGAGUGUCGUGCAGCCUCCAGGAC
377. KY620406.1/19-136 ACCUGCCUCUUA--C----GAGG-CGACACUCCACCAUGGAUCACUCCCCUGUGAGGAACUUCUGUCUUCACGCGGAAAGCGCCUAGCCAUGGCGUUAGUACGAGUGUCGUGCAGCCUCCAGGAC
378. KY620403.1/17-134 ACCUGCCUCUUA--C----GAGG-CGACACUCCACCAUGGAUCACUCCCCUGUGAGGAACUUCUGUCUUCACGCGGAAAGCGCCUAGCCAUGGCGUUAGUACGAGUGUCGUGCAGCCUCCAGGAC
379. KY620384.1/22-139 ACCUGCCUCUUA--C----GAGG-CGACACUCCACCAUGGAUCACUCCCCUGUGAGGAACUUCUGUCUUCACGCGGAAAGCGCCUAGCCAUGGCGUUAGUACGAGUGUCGUGCAGCCUCCAGGAC
380. KY620381.1/15-132 ACCUGCCUCUUA--C----GAGG-CGACACUCCACCAUGGAUCACUCCCCUGUGAGGAACUUCUGUCUUCACGCGGAAAGCGCCUAGCCAUGGCGUUAGUACGAGUGUCGUGCAGCCUCCAGGAC
381. KY620375.1/14-131 ACCUGCCUCUUA--C----GAGG-CGACACUCCACCAUGGAUCACUCCCCUGUGAGGAACUUCUGUCUUCACGCGGAAAGCGCCUAGCCAUGGCGUUAGUACGAGUGUCGUGCAGCCUCCAGGAC
382. KY620371.1/14-131 ACCUGCCUCUUA--C----GAGG-CGACACUCCACCAUGGAUCACUCCCCUGUGAGGAACUUCUGUCUUCACGCGGAAAGCGCCUAGCCAUGGCGUUAGUACGAGUGUCGUGCAGCCUCCAGGAC
383. KY620366.1/15-132 ACCUGCCUCUUA--C----GAGG-CGACACUCCACCAUGGAUCACUCCCCUGUGAGGAACUUCUGUCUUCACGCGGAAAGCGCCUAGCCAUGGCGUUAGUACGAGUGUCGUGCAGCCUCCAGGAC
384. KY620864.1/21-138 ACCUGCCUCUUA--C----GAGG-CGACACUCCACCAUGGAUCACUCCCCUGUGAGGAACUUCUGUCUUCACGCGGAAAGCGCCUAGCCAUGGCGUUAGUACGAGUGUCGUGCAGCCUCCAGGAC
385. KY620853.1/21-138 ACCUGCCUCUUA--C----GAGG-CGACACUCCACCAUGGAUCACUCCCCUGUGAGGAACUUCUGUCUUCACGCGGAAAGCGCCUAGCCAUGGCGUUAGUACGAGUGUCGUGCAGCCUCCAGGAC
386. KY620845.1/12-129 ACCUGCCUCUUA--C----GAGG-CGACACUCCACCAUGGAUCACUCCCCUGUGAGGAACUUCUGUCUUCACGCGGAAAGCGCCUAGCCAUGGCGUUAGUACGAGUGUCGUGCAGCCUCCAGGAC
387. KY620837.1/22-139 ACCUGCCUCUUA--C----GAGG-CGACACUCCACCAUGGAUCACUCCCCUGUGAGGAACUUCUGUCUUCACGCGGAAAGCGCCUAGCCAUGGCGUUAGUACGAGUGUCGUGCAGCCUCCAGGAC
388. KY620835.1/20-137 ACCUGCCUCUUA--C----GAGG-CGACACUCCACCAUGGAUCACUCCCCUGUGAGGAACUUCUGUCUUCACGCGGAAAGCGCCUAGCCAUGGCGUUAGUACGAGUGUCGUGCAGCCUCCAGGAC
389. KY620824.1/21-138 ACCUGCCUCUUA--C----GAGG-CGACACUCCACCAUGGAUCACUCCCCUGUGAGGAACUUCUGUCUUCACGCGGAAAGCGCCUAGCCAUGGCGUUAGUACGAGUGUCGUGCAGCCUCCAGGAC
390. KY620811.1/20-137 ACCUGCCUCUUA--C----GAGG-CGACACUCCACCAUGGAUCACUCCCCUGUGAGGAACUUCUGUCUUCACGCGGAAAGCGCCUAGCCAUGGCGUUAGUACGAGUGUCGUGCAGCCUCCAGGAC
391. KY620801.1/19-136 ACCUGCCUCUUA--C----GAGG-CGACACUCCACCAUGGAUCACUCCCCUGUGAGGAACUUCUGUCUUCACGCGGAAAGCGCCUAGCCAUGGCGUUAGUACGAGUGUCGUGCAGCCUCCAGGAC
392. KY620797.1/23-140 ACCUGCCUCUUA--C----GAGG-CGACACUCCACCAUGGAUCACUCCCCUGUGAGGAACUUCUGUCUUCACGCGGAAAGCGCCUAGCCAUGGCGUUAGUACGAGUGUCGUGCAGCCUCCAGGAC
393. KY620792.1/18-135 ACCUGCCUCUUA--C----GAGG-CGACACUCCACCAUGGAUCACUCCCCUGUGAGGAACUUCUGUCUUCACGCGGAAAGCGCCUAGCCAUGGCGUUAGUACGAGUGUCGUGCAGCCUCCAGGAC
394. KY620790.1/22-139 ACCUGCCUCUUA--C----GAGG-CGACACUCCACCAUGGAUCACUCCCCUGUGAGGAACUUCUGUCUUCACGCGGAAAGCGCCUAGCCAUGGCGUUAGUACGAGUGUCGUGCAGCCUCCAGGAC
395. KY620786.1/18-135 ACCUGCCUCUUA--C----GAGG-CGACACUCCACCAUGGAUCACUCCCCUGUGAGGAACUUCUGUCUUCACGCGGAAAGCGCCUAGCCAUGGCGUUAGUACGAGUGUCGUGCAGCCUCCAGGAC
396. KY620770.1/21-138 ACCUGCCUCUUA--C----GAGG-CGACACUCCACCAUGGAUCACUCCCCUGUGAGGAACUUCUGUCUUCACGCGGAAAGCGCCUAGCCAUGGCGUUAGUACGAGUGUCGUGCAGCCUCCAGGAC
397. KY620769.1/16-133 ACCUGCCUCUUA--C----GAGG-CGACACUCCACCAUGGAUCACUCCCCUGUGAGGAACUUCUGUCUUCACGCGGAAAGCGCCUAGCCAUGGCGUUAGUACGAGUGUCGUGCAGCCUCCAGGAC
398. KY620765.1/14-131 ACCUGCCUCUUA--C----GAGG-CGACACUCCACCAUGGAUCACUCCCCUGUGAGGAACUUCUGUCUUCACGCGGAAAGCGCCUAGCCAUGGCGUUAGUACGAGUGUCGUGCAGCCUCCAGGAC
399. KY620764.1/23-140 ACCUGCCUCUUA--C----GAGG-CGACACUCCACCAUGGAUCACUCCCCUGUGAGGAACUUCUGUCUUCACGCGGAAAGCGCCUAGCCAUGGCGUUAGUACGAGUGUCGUGCAGCCUCCAGGAC
400. KY620763.1/21-138 ACCUGCCUCUUA--C----GAGG-CGACACUCCACCAUGGAUCACUCCCCUGUGAGGAACUUCUGUCUUCACGCGGAAAGCGCCUAGCCAUGGCGUUAGUACGAGUGUCGUGCAGCCUCCAGGAC
401. KY620759.1/12-129 ACCUGCCUCUUA--C----GAGG-CGACACUCCACCAUGGAUCACUCCCCUGUGAGGAACUUCUGUCUUCACGCGGAAAGCGCCUAGCCAUGGCGUUAGUACGAGUGUCGUGCAGCCUCCAGGAC
402. KY620754.1/22-139 ACCUGCCUCUUA--C----GAGG-CGACACUCCACCAUGGAUCACUCCCCUGUGAGGAACUUCUGUCUUCACGCGGAAAGCGCCUAGCCAUGGCGUUAGUACGAGUGUCGUGCAGCCUCCAGGAC
403. KY620749.1/20-137 ACCUGCCUCUUA--C----GAGG-CGACACUCCACCAUGGAUCACUCCCCUGUGAGGAACUUCUGUCUUCACGCGGAAAGCGCCUAGCCAUGGCGUUAGUACGAGUGUCGUGCAGCCUCCAGGAC
404. KY620747.1/21-138 ACCUGCCUCUUA--C----GAGG-CGACACUCCACCAUGGAUCACUCCCCUGUGAGGAACUUCUGUCUUCACGCGGAAAGCGCCUAGCCAUGGCGUUAGUACGAGUGUCGUGCAGCCUCCAGGAC
405. KY620746.1/22-139 ACCUGCCUCUUA--C----GAGG-CGACACUCCACCAUGGAUCACUCCCCUGUGAGGAACUUCUGUCUUCACGCGGAAAGCGCCUAGCCAUGGCGUUAGUACGAGUGUCGUGCAGCCUCCAGGAC
406. KY620742.1/18-135 ACCUGCCUCUUA--C----GAGG-CGACACUCCACCAUGGAUCACUCCCCUGUGAGGAACUUCUGUCUUCACGCGGAAAGCGCCUAGCCAUGGCGUUAGUACGAGUGUCGUGCAGCCUCCAGGAC
407. KY620736.1/8-125 ACCUGCCUCUUA--C----GAGG-CGACACUCCACCAUGGAUCACUCCCCUGUGAGGAACUUCUGUCUUCACGCGGAAAGCGCCUAGCCAUGGCGUUAGUACGAGUGUCGUGCAGCCUCCAGGAC
408. KY620734.1/18-135 ACCUGCCUCUUA--C----GAGG-CGACACUCCACCAUGGAUCACUCCCCUGUGAGGAACUUCUGUCUUCACGCGGAAAGCGCCUAGCCAUGGCGUUAGUACGAGUGUCGUGCAGCCUCCAGGAC
409. KY620722.1/17-134 ACCUGCCUCUUA--C----GAGG-CGACACUCCACCAUGGAUCACUCCCCUGUGAGGAACUUCUGUCUUCACGCGGAAAGCGCCUAGCCAUGGCGUUAGUACGAGUGUCGUGCAGCCUCCAGGAC
410. KY620717.1/22-139 ACCUGCCUCUUA--C----GAGG-CGACACUCCACCAUGGAUCACUCCCCUGUGAGGAACUUCUGUCUUCACGCGGAAAGCGCCUAGCCAUGGCGUUAGUACGAGUGUCGUGCAGCCUCCAGGAC
411. KY620710.1/22-139 ACCUGCCUCUUA--C----GAGG-CGACACUCCACCAUGGAUCACUCCCCUGUGAGGAACUUCUGUCUUCACGCGGAAAGCGCCUAGCCAUGGCGUUAGUACGAGUGUCGUGCAGCCUCCAGGAC
412. KY620709.1/17-134 ACCUGCCUCUUA--C----GAGG-CGACACUCCACCAUGGAUCACUCCCCUGUGAGGAACUUCUGUCUUCACGCGGAAAGCGCCUAGCCAUGGCGUUAGUACGAGUGUCGUGCAGCCUCCAGGAC
413. KY620707.1/27-144 ACCUGCCUCUUA--C----GAGG-CGACACUCCACCAUGGAUCACUCCCCUGUGAGGAACUUCUGUCUUCACGCGGAAAGCGCCUAGCCAUGGCGUUAGUACGAGUGUCGUGCAGCCUCCAGGAC
414. KY620706.1/18-135 ACCUGCCUCUUA--C----GAGG-CGACACUCCACCAUGGAUCACUCCCCUGUGAGGAACUUCUGUCUUCACGCGGAAAGCGCCUAGCCAUGGCGUUAGUACGAGUGUCGUGCAGCCUCCAGGAC
415. KY620702.1/6-123 ACCUGCCUCUUA--C----GAGG-CGACACUCCACCAUGGAUCACUCCCCUGUGAGGAACUUCUGUCUUCACGCGGAAAGCGCCUAGCCAUGGCGUUAGUACGAGUGUCGUGCAGCCUCCAGGAC
416. KY620701.1/17-134 ACCUGCCUCUUA--C----GAGG-CGACACUCCACCAUGGAUCACUCCCCUGUGAGGAACUUCUGUCUUCACGCGGAAAGCGCCUAGCCAUGGCGUUAGUACGAGUGUCGUGCAGCCUCCAGGAC
417. KY620700.1/7-124 ACCUGCCUCUUA--C----GAGG-CGACACUCCACCAUGGAUCACUCCCCUGUGAGGAACUUCUGUCUUCACGCGGAAAGCGCCUAGCCAUGGCGUUAGUACGAGUGUCGUGCAGCCUCCAGGAC
418. KY620698.1/22-139 ACCUGCCUCUUA--C----GAGG-CGACACUCCACCAUGGAUCACUCCCCUGUGAGGAACUUCUGUCUUCACGCGGAAAGCGCCUAGCCAUGGCGUUAGUACGAGUGUCGUGCAGCCUCCAGGAC
419. KY620697.1/6-123 ACCUGCCUCUUA--C----GAGG-CGACACUCCACCAUGGAUCACUCCCCUGUGAGGAACUUCUGUCUUCACGCGGAAAGCGCCUAGCCAUGGCGUUAGUACGAGUGUCGUGCAGCCUCCAGGAC
420. KY620694.1/21-138 ACCUGCCUCUUA--C----GAGG-CGACACUCCACCAUGGAUCACUCCCCUGUGAGGAACUUCUGUCUUCACGCGGAAAGCGCCUAGCCAUGGCGUUAGUACGAGUGUCGUGCAGCCUCCAGGAC
421. KY620685.1/18-135 ACCUGCCUCUUA--C----GAGG-CGACACUCCACCAUGGAUCACUCCCCUGUGAGGAACUUCUGUCUUCACGCGGAAAGCGCCUAGCCAUGGCGUUAGUACGAGUGUCGUGCAGCCUCCAGGAC
422. KY620681.1/21-138 ACCUGCCUCUUA--C----GAGG-CGACACUCCACCAUGGAUCACUCCCCUGUGAGGAACUUCUGUCUUCACGCGGAAAGCGCCUAGCCAUGGCGUUAGUACGAGUGUCGUGCAGCCUCCAGGAC
423. KY620680.1/23-140 ACCUGCCUCUUA--C----GAGG-CGACACUCCACCAUGGAUCACUCCCCUGUGAGGAACUUCUGUCUUCACGCGGAAAGCGCCUAGCCAUGGCGUUAGUACGAGUGUCGUGCAGCCUCCAGGAC
424. KY620678.1/21-138 ACCUGCCUCUUA--C----GAGG-CGACACUCCACCAUGGAUCACUCCCCUGUGAGGAACUUCUGUCUUCACGCGGAAAGCGCCUAGCCAUGGCGUUAGUACGAGUGUCGUGCAGCCUCCAGGAC
425. KY620674.1/22-139 ACCUGCCUCUUA--C----GAGG-CGACACUCCACCAUGGAUCACUCCCCUGUGAGGAACUUCUGUCUUCACGCGGAAAGCGCCUAGCCAUGGCGUUAGUACGAGUGUCGUGCAGCCUCCAGGAC
426. KY620671.1/25-142 ACCUGCCUCUUA--C----GAGG-CGACACUCCACCAUGGAUCACUCCCCUGUGAGGAACUUCUGUCUUCACGCGGAAAGCGCCUAGCCAUGGCGUUAGUACGAGUGUCGUGCAGCCUCCAGGAC
427. KY620670.1/21-138 ACCUGCCUCUUA--C----GAGG-CGACACUCCACCAUGGAUCACUCCCCUGUGAGGAACUUCUGUCUUCACGCGGAAAGCGCCUAGCCAUGGCGUUAGUACGAGUGUCGUGCAGCCUCCAGGAC
428. KY620666.1/22-139 ACCUGCCUCUUA--C----GAGG-CGACACUCCACCAUGGAUCACUCCCCUGUGAGGAACUUCUGUCUUCACGCGGAAAGCGCCUAGCCAUGGCGUUAGUACGAGUGUCGUGCAGCCUCCAGGAC
429. KY620665.1/20-137 ACCUGCCUCUUA--C----GAGG-CGACACUCCACCAUGGAUCACUCCCCUGUGAGGAACUUCUGUCUUCACGCGGAAAGCGCCUAGCCAUGGCGUUAGUACGAGUGUCGUGCAGCCUCCAGGAC
430. KY620663.1/21-138 ACCUGCCUCUUA--C----GAGG-CGACACUCCACCAUGGAUCACUCCCCUGUGAGGAACUUCUGUCUUCACGCGGAAAGCGCCUAGCCAUGGCGUUAGUACGAGUGUCGUGCAGCCUCCAGGAC
431. KY620662.1/20-137 ACCUGCCUCUUA--C----GAGG-CGACACUCCACCAUGGAUCACUCCCCUGUGAGGAACUUCUGUCUUCACGCGGAAAGCGCCUAGCCAUGGCGUUAGUACGAGUGUCGUGCAGCCUCCAGGAC
432. KY620649.1/20-137 ACCUGCCUCUUA--C----GAGG-CGACACUCCACCAUGGAUCACUCCCCUGUGAGGAACUUCUGUCUUCACGCGGAAAGCGCCUAGCCAUGGCGUUAGUACGAGUGUCGUGCAGCCUCCAGGAC
433. KY620646.1/23-140 ACCUGCCUCUUA--C----GAGG-CGACACUCCACCAUGGAUCACUCCCCUGUGAGGAACUUCUGUCUUCACGCGGAAAGCGCCUAGCCAUGGCGUUAGUACGAGUGUCGUGCAGCCUCCAGGAC
434. KY620637.1/20-137 ACCUGCCUCUUA--C----GAGG-CGACACUCCACCAUGGAUCACUCCCCUGUGAGGAACUUCUGUCUUCACGCGGAAAGCGCCUAGCCAUGGCGUUAGUACGAGUGUCGUGCAGCCUCCAGGAC
435. KY620632.1/21-138 ACCUGCCUCUUA--C----GAGG-CGACACUCCACCAUGGAUCACUCCCCUGUGAGGAACUUCUGUCUUCACGCGGAAAGCGCCUAGCCAUGGCGUUAGUACGAGUGUCGUGCAGCCUCCAGGAC
436. KY620631.1/21-138 ACCUGCCUCUUA--C----GAGG-CGACACUCCACCAUGGAUCACUCCCCUGUGAGGAACUUCUGUCUUCACGCGGAAAGCGCCUAGCCAUGGCGUUAGUACGAGUGUCGUGCAGCCUCCAGGAC
437. KY620628.1/21-138 ACCUGCCUCUUA--C----GAGG-CGACACUCCACCAUGGAUCACUCCCCUGUGAGGAACUUCUGUCUUCACGCGGAAAGCGCCUAGCCAUGGCGUUAGUACGAGUGUCGUGCAGCCUCCAGGAC
438. KY620625.1/23-140 ACCUGCCUCUUA--C----GAGG-CGACACUCCACCAUGGAUCACUCCCCUGUGAGGAACUUCUGUCUUCACGCGGAAAGCGCCUAGCCAUGGCGUUAGUACGAGUGUCGUGCAGCCUCCAGGAC
439. KY620615.1/25-142 ACCUGCCUCUUA--C----GAGG-CGACACUCCACCAUGGAUCACUCCCCUGUGAGGAACUUCUGUCUUCACGCGGAAAGCGCCUAGCCAUGGCGUUAGUACGAGUGUCGUGCAGCCUCCAGGAC
440. KY620613.1/19-136 ACCUGCCUCUUA--C----GAGG-CGACACUCCACCAUGGAUCACUCCCCUGUGAGGAACUUCUGUCUUCACGCGGAAAGCGCCUAGCCAUGGCGUUAGUACGAGUGUCGUGCAGCCUCCAGGAC
441. KY620609.1/20-137 ACCUGCCUCUUA--C----GAGG-CGACACUCCACCAUGGAUCACUCCCCUGUGAGGAACUUCUGUCUUCACGCGGAAAGCGCCUAGCCAUGGCGUUAGUACGAGUGUCGUGCAGCCUCCAGGAC
442. KY620607.1/14-131 ACCUGCCUCUUA--C----GAGG-CGACACUCCACCAUGGAUCACUCCCCUGUGAGGAACUUCUGUCUUCACGCGGAAAGCGCCUAGCCAUGGCGUUAGUACGAGUGUCGUGCAGCCUCCAGGAC
443. KY620601.1/22-139 ACCUGCCUCUUA--C----GAGG-CGACACUCCACCAUGGAUCACUCCCCUGUGAGGAACUUCUGUCUUCACGCGGAAAGCGCCUAGCCAUGGCGUUAGUACGAGUGUCGUGCAGCCUCCAGGAC
444. KY620600.1/24-141 ACCUGCCUCUUA--C----GAGG-CGACACUCCACCAUGGAUCACUCCCCUGUGAGGAACUUCUGUCUUCACGCGGAAAGCGCCUAGCCAUGGCGUUAGUACGAGUGUCGUGCAGCCUCCAGGAC
445. KY620598.1/14-131 ACCUGCCUCUUA--C----GAGG-CGACACUCCACCAUGGAUCACUCCCCUGUGAGGAACUUCUGUCUUCACGCGGAAAGCGCCUAGCCAUGGCGUUAGUACGAGUGUCGUGCAGCCUCCAGGAC
446. KY620597.1/20-137 ACCUGCCUCUUA--C----GAGG-CGACACUCCACCAUGGAUCACUCCCCUGUGAGGAACUUCUGUCUUCACGCGGAAAGCGCCUAGCCAUGGCGUUAGUACGAGUGUCGUGCAGCCUCCAGGAC
447. KY620586.1/16-133 ACCUGCCUCUUA--C----GAGG-CGACACUCCACCAUGGAUCACUCCCCUGUGAGGAACUUCUGUCUUCACGCGGAAAGCGCCUAGCCAUGGCGUUAGUACGAGUGUCGUGCAGCCUCCAGGAC
448. KY620585.1/21-138 ACCUGCCUCUUA--C----GAGG-CGACACUCCACCAUGGAUCACUCCCCUGUGAGGAACUUCUGUCUUCACGCGGAAAGCGCCUAGCCAUGGCGUUAGUACGAGUGUCGUGCAGCCUCCAGGAC
449. KY620583.1/22-139 ACCUGCCUCUUA--C----GAGG-CGACACUCCACCAUGGAUCACUCCCCUGUGAGGAACUUCUGUCUUCACGCGGAAAGCGCCUAGCCAUGGCGUUAGUACGAGUGUCGUGCAGCCUCCAGGAC
450. KY620577.1/22-139 ACCUGCCUCUUA--C----GAGG-CGACACUCCACCAUGGAUCACUCCCCUGUGAGGAACUUCUGUCUUCACGCGGAAAGCGCCUAGCCAUGGCGUUAGUACGAGUGUCGUGCAGCCUCCAGGAC
451. KY620574.1/21-138 ACCUGCCUCUUA--C----GAGG-CGACACUCCACCAUGGAUCACUCCCCUGUGAGGAACUUCUGUCUUCACGCGGAAAGCGCCUAGCCAUGGCGUUAGUACGAGUGUCGUGCAGCCUCCAGGAC
452. KY620568.1/21-138 ACCUGCCUCUUA--C----GAGG-CGACACUCCACCAUGGAUCACUCCCCUGUGAGGAACUUCUGUCUUCACGCGGAAAGCGCCUAGCCAUGGCGUUAGUACGAGUGUCGUGCAGCCUCCAGGAC
453. KY620566.1/22-139 ACCUGCCUCUUA--C----GAGG-CGACACUCCACCAUGGAUCACUCCCCUGUGAGGAACUUCUGUCUUCACGCGGAAAGCGCCUAGCCAUGGCGUUAGUACGAGUGUCGUGCAGCCUCCAGGAC
454. KY620565.1/22-139 ACCUGCCUCUUA--C----GAGG-CGACACUCCACCAUGGAUCACUCCCCUGUGAGGAACUUCUGUCUUCACGCGGAAAGCGCCUAGCCAUGGCGUUAGUACGAGUGUCGUGCAGCCUCCAGGAC
455. KY620554.1/21-138 ACCUGCCUCUUA--C----GAGG-CGACACUCCACCAUGGAUCACUCCCCUGUGAGGAACUUCUGUCUUCACGCGGAAAGCGCCUAGCCAUGGCGUUAGUACGAGUGUCGUGCAGCCUCCAGGAC
456. KY620553.1/23-140 ACCUGCCUCUUA--C----GAGG-CGACACUCCACCAUGGAUCACUCCCCUGUGAGGAACUUCUGUCUUCACGCGGAAAGCGCCUAGCCAUGGCGUUAGUACGAGUGUCGUGCAGCCUCCAGGAC
457. KY620552.1/15-132 ACCUGCCUCUUA--C----GAGG-CGACACUCCACCAUGGAUCACUCCCCUGUGAGGAACUUCUGUCUUCACGCGGAAAGCGCCUAGCCAUGGCGUUAGUACGAGUGUCGUGCAGCCUCCAGGAC
458. KY620551.1/22-139 ACCUGCCUCUUA--C----GAGG-CGACACUCCACCAUGGAUCACUCCCCUGUGAGGAACUUCUGUCUUCACGCGGAAAGCGCCUAGCCAUGGCGUUAGUACGAGUGUCGUGCAGCCUCCAGGAC
459. KY620550.1/4-121 ACCUGCCUCUUA--C----GAGG-CGACACUCCACCAUGGAUCACUCCCCUGUGAGGAACUUCUGUCUUCACGCGGAAAGCGCCUAGCCAUGGCGUUAGUACGAGUGUCGUGCAGCCUCCAGGAC
460. KY620543.1/14-131 ACCUGCCUCUUA--C----GAGG-CGACACUCCACCAUGGAUCACUCCCCUGUGAGGAACUUCUGUCUUCACGCGGAAAGCGCCUAGCCAUGGCGUUAGUACGAGUGUCGUGCAGCCUCCAGGAC
461. KY620538.1/22-139 ACCUGCCUCUUA--C----GAGG-CGACACUCCACCAUGGAUCACUCCCCUGUGAGGAACUUCUGUCUUCACGCGGAAAGCGCCUAGCCAUGGCGUUAGUACGAGUGUCGUGCAGCCUCCAGGAC
462. KY620537.1/20-137 ACCUGCCUCUUA--C----GAGG-CGACACUCCACCAUGGAUCACUCCCCUGUGAGGAACUUCUGUCUUCACGCGGAAAGCGCCUAGCCAUGGCGUUAGUACGAGUGUCGUGCAGCCUCCAGGAC
463. KY620533.1/22-139 ACCUGCCUCUUA--C----GAGG-CGACACUCCACCAUGGAUCACUCCCCUGUGAGGAACUUCUGUCUUCACGCGGAAAGCGCCUAGCCAUGGCGUUAGUACGAGUGUCGUGCAGCCUCCAGGAC
464. KY620527.1/20-137 ACCUGCCUCUUA--C----GAGG-CGACACUCCACCAUGGAUCACUCCCCUGUGAGGAACUUCUGUCUUCACGCGGAAAGCGCCUAGCCAUGGCGUUAGUACGAGUGUCGUGCAGCCUCCAGGAC
465. KY620526.1/23-140 ACCUGCCUCUUA--C----GAGG-CGACACUCCACCAUGGAUCACUCCCCUGUGAGGAACUUCUGUCUUCACGCGGAAAGCGCCUAGCCAUGGCGUUAGUACGAGUGUCGUGCAGCCUCCAGGAC
466. KY620519.1/21-138 ACCUGCCUCUUA--C----GAGG-CGACACUCCACCAUGGAUCACUCCCCUGUGAGGAACUUCUGUCUUCACGCGGAAAGCGCCUAGCCAUGGCGUUAGUACGAGUGUCGUGCAGCCUCCAGGAC
467. KY620514.1/21-138 ACCUGCCUCUUA--C----GAGG-CGACACUCCACCAUGGAUCACUCCCCUGUGAGGAACUUCUGUCUUCACGCGGAAAGCGCCUAGCCAUGGCGUUAGUACGAGUGUCGUGCAGCCUCCAGGAC
468. KY620512.1/18-135 ACCUGCCUCUUA--C----GAGG-CGACACUCCACCAUGGAUCACUCCCCUGUGAGGAACUUCUGUCUUCACGCGGAAAGCGCCUAGCCAUGGCGUUAGUACGAGUGUCGUGCAGCCUCCAGGAC
469. KY620511.1/6-123 ACCUGCCUCUUA--C----GAGG-CGACACUCCACCAUGGAUCACUCCCCUGUGAGGAACUUCUGUCUUCACGCGGAAAGCGCCUAGCCAUGGCGUUAGUACGAGUGUCGUGCAGCCUCCAGGAC
470. KY620510.1/21-138 ACCUGCCUCUUA--C----GAGG-CGACACUCCACCAUGGAUCACUCCCCUGUGAGGAACUUCUGUCUUCACGCGGAAAGCGCCUAGCCAUGGCGUUAGUACGAGUGUCGUGCAGCCUCCAGGAC
471. KY620508.1/6-123 ACCUGCCUCUUA--C----GAGG-CGACACUCCACCAUGGAUCACUCCCCUGUGAGGAACUUCUGUCUUCACGCGGAAAGCGCCUAGCCAUGGCGUUAGUACGAGUGUCGUGCAGCCUCCAGGAC
472. KY620505.1/12-129 ACCUGCCUCUUA--C----GAGG-CGACACUCCACCAUGGAUCACUCCCCUGUGAGGAACUUCUGUCUUCACGCGGAAAGCGCCUAGCCAUGGCGUUAGUACGAGUGUCGUGCAGCCUCCAGGAC
473. KY620503.1/22-139 ACCUGCCUCUUA--C----GAGG-CGACACUCCACCAUGGAUCACUCCCCUGUGAGGAACUUCUGUCUUCACGCGGAAAGCGCCUAGCCAUGGCGUUAGUACGAGUGUCGUGCAGCCUCCAGGAC
474. KY620493.1/18-135 ACCUGCCUCUUA--C----GAGG-CGACACUCCACCAUGGAUCACUCCCCUGUGAGGAACUUCUGUCUUCACGCGGAAAGCGCCUAGCCAUGGCGUUAGUACGAGUGUCGUGCAGCCUCCAGGAC
475. KY620490.1/15-132 ACCUGCCUCUUA--C----GAGG-CGACACUCCACCAUGGAUCACUCCCCUGUGAGGAACUUCUGUCUUCACGCGGAAAGCGCCUAGCCAUGGCGUUAGUACGAGUGUCGUGCAGCCUCCAGGAC
476. KY620488.1/22-139 ACCUGCCUCUUA--C----GAGG-CGACACUCCACCAUGGAUCACUCCCCUGUGAGGAACUUCUGUCUUCACGCGGAAAGCGCCUAGCCAUGGCGUUAGUACGAGUGUCGUGCAGCCUCCAGGAC
477. KY620486.1/21-138 ACCUGCCUCUUA--C----GAGG-CGACACUCCACCAUGGAUCACUCCCCUGUGAGGAACUUCUGUCUUCACGCGGAAAGCGCCUAGCCAUGGCGUUAGUACGAGUGUCGUGCAGCCUCCAGGAC
478. KY620484.1/10-127 ACCUGCCUCUUA--C----GAGG-CGACACUCCACCAUGGAUCACUCCCCUGUGAGGAACUUCUGUCUUCACGCGGAAAGCGCCUAGCCAUGGCGUUAGUACGAGUGUCGUGCAGCCUCCAGGAC
479. KY620482.1/26-143 ACCUGCCUCUUA--C----GAGG-CGACACUCCACCAUGGAUCACUCCCCUGUGAGGAACUUCUGUCUUCACGCGGAAAGCGCCUAGCCAUGGCGUUAGUACGAGUGUCGUGCAGCCUCCAGGAC
480. KY620476.1/22-139 ACCUGCCUCUUA--C----GAGG-CGACACUCCACCAUGGAUCACUCCCCUGUGAGGAACUUCUGUCUUCACGCGGAAAGCGCCUAGCCAUGGCGUUAGUACGAGUGUCGUGCAGCCUCCAGGAC
481. KY620474.1/21-138 ACCUGCCUCUUA--C----GAGG-CGACACUCCACCAUGGAUCACUCCCCUGUGAGGAACUUCUGUCUUCACGCGGAAAGCGCCUAGCCAUGGCGUUAGUACGAGUGUCGUGCAGCCUCCAGGAC
482. KY620465.1/12-129 ACCUGCCUCUUA--C----GAGG-CGACACUCCACCAUGGAUCACUCCCCUGUGAGGAACUUCUGUCUUCACGCGGAAAGCGCCUAGCCAUGGCGUUAGUACGAGUGUCGUGCAGCCUCCAGGAC
483. KY620461.1/20-137 ACCUGCCUCUUA--C----GAGG-CGACACUCCACCAUGGAUCACUCCCCUGUGAGGAACUUCUGUCUUCACGCGGAAAGCGCCUAGCCAUGGCGUUAGUACGAGUGUCGUGCAGCCUCCAGGAC
484. KY620457.1/20-137 ACCUGCCUCUUA--C----GAGG-CGACACUCCACCAUGGAUCACUCCCCUGUGAGGAACUUCUGUCUUCACGCGGAAAGCGCCUAGCCAUGGCGUUAGUACGAGUGUCGUGCAGCCUCCAGGAC
485. KY620455.1/21-138 ACCUGCCUCUUA--C----GAGG-CGACACUCCACCAUGGAUCACUCCCCUGUGAGGAACUUCUGUCUUCACGCGGAAAGCGCCUAGCCAUGGCGUUAGUACGAGUGUCGUGCAGCCUCCAGGAC
486. KY620453.1/20-137 ACCUGCCUCUUA--C----GAGG-CGACACUCCACCAUGGAUCACUCCCCUGUGAGGAACUUCUGUCUUCACGCGGAAAGCGCCUAGCCAUGGCGUUAGUACGAGUGUCGUGCAGCCUCCAGGAC
487. KY620451.1/21-138 ACCUGCCUCUUA--C----GAGG-CGACACUCCACCAUGGAUCACUCCCCUGUGAGGAACUUCUGUCUUCACGCGGAAAGCGCCUAGCCAUGGCGUUAGUACGAGUGUCGUGCAGCCUCCAGGAC
488. KY620442.1/23-140 ACCUGCCUCUUA--C----GAGG-CGACACUCCACCAUGGAUCACUCCCCUGUGAGGAACUUCUGUCUUCACGCGGAAAGCGCCUAGCCAUGGCGUUAGUACGAGUGUCGUGCAGCCUCCAGGAC
489. KY620441.1/22-139 ACCUGCCUCUUA--C----GAGG-CGACACUCCACCAUGGAUCACUCCCCUGUGAGGAACUUCUGUCUUCACGCGGAAAGCGCCUAGCCAUGGCGUUAGUACGAGUGUCGUGCAGCCUCCAGGAC
490. KY620429.1/20-137 ACCUGCCUCUUA--C----GAGG-CGACACUCCACCAUGGAUCACUCCCCUGUGAGGAACUUCUGUCUUCACGCGGAAAGCGCCUAGCCAUGGCGUUAGUACGAGUGUCGUGCAGCCUCCAGGAC
491. KY620426.1/7-124 ACCUGCCUCUUA--C----GAGG-CGACACUCCACCAUGGAUCACUCCCCUGUGAGGAACUUCUGUCUUCACGCGGAAAGCGCCUAGCCAUGGCGUUAGUACGAGUGUCGUGCAGCCUCCAGGAC
492. KY620424.1/21-138 ACCUGCCUCUUA--C----GAGG-CGACACUCCACCAUGGAUCACUCCCCUGUGAGGAACUUCUGUCUUCACGCGGAAAGCGCCUAGCCAUGGCGUUAGUACGAGUGUCGUGCAGCCUCCAGGAC
493. KY620414.1/22-139 ACCUGCCUCUUA--C----GAGG-CGACACUCCACCAUGGAUCACUCCCCUGUGAGGAACUUCUGUCUUCACGCGGAAAGCGCCUAGCCAUGGCGUUAGUACGAGUGUCGUGCAGCCUCCAGGAC
494. KY620392.1/22-139 ACCUGCCUCUUA--C----GAGG-CGACACUCCACCAUGGAUCACUCCCCUGUGAGGAACUUCUGUCUUCACGCGGAAAGCGCCUAGCCAUGGCGUUAGUACGAGUGUCGUGCAGCCUCCAGGAC
495. KY620389.1/22-139 ACCUGCCUCUUA--C----GAGG-CGACACUCCACCAUGGAUCACUCCCCUGUGAGGAACUUCUGUCUUCACGCGGAAAGCGCCUAGCCAUGGCGUUAGUACGAGUGUCGUGCAGCCUCCAGGAC
496. KY620385.1/22-139 ACCUGCCUCUUA--C----GAGG-CGACACUCCACCAUGGAUCACUCCCCUGUGAGGAACUUCUGUCUUCACGCGGAAAGCGCCUAGCCAUGGCGUUAGUACGAGUGUCGUGCAGCCUCCAGGAC
497. KY620380.1/22-139 ACCUGCCUCUUA--C----GAGG-CGACACUCCACCAUGGAUCACUCCCCUGUGAGGAACUUCUGUCUUCACGCGGAAAGCGCCUAGCCAUGGCGUUAGUACGAGUGUCGUGCAGCCUCCAGGAC
498. KY620379.1/13-130 ACCUGCCUCUUA--C----GAGG-CGACACUCCACCAUGGAUCACUCCCCUGUGAGGAACUUCUGUCUUCACGCGGAAAGCGCCUAGCCAUGGCGUUAGUACGAGUGUCGUGCAGCCUCCAGGAC
499. KY620378.1/23-140 ACCUGCCUCUUA--C----GAGG-CGACACUCCACCAUGGAUCACUCCCCUGUGAGGAACUUCUGUCUUCACGCGGAAAGCGCCUAGCCAUGGCGUUAGUACGAGUGUCGUGCAGCCUCCAGGAC
500. KY620377.1/21-138 ACCUGCCUCUUA--C----GAGG-CGACACUCCACCAUGGAUCACUCCCCUGUGAGGAACUUCUGUCUUCACGCGGAAAGCGCCUAGCCAUGGCGUUAGUACGAGUGUCGUGCAGCCUCCAGGAC
501. KY620376.1/16-133 ACCUGCCUCUUA--C----GAGG-CGACACUCCACCAUGGAUCACUCCCCUGUGAGGAACUUCUGUCUUCACGCGGAAAGCGCCUAGCCAUGGCGUUAGUACGAGUGUCGUGCAGCCUCCAGGAC
502. KY620374.1/9-126 ACCUGCCUCUUA--C----GAGG-CGACACUCCACCAUGGAUCACUCCCCUGUGAGGAACUUCUGUCUUCACGCGGAAAGCGCCUAGCCAUGGCGUUAGUACGAGUGUCGUGCAGCCUCCAGGAC
503. KY620372.1/7-124 ACCUGCCUCUUA--C----GAGG-CGACACUCCACCAUGGAUCACUCCCCUGUGAGGAACUUCUGUCUUCACGCGGAAAGCGCCUAGCCAUGGCGUUAGUACGAGUGUCGUGCAGCCUCCAGGAC
504. KY620367.1/17-134 ACCUGCCUCUUA--C----GAGG-CGACACUCCACCAUGGAUCACUCCCCUGUGAGGAACUUCUGUCUUCACGCGGAAAGCGCCUAGCCAUGGCGUUAGUACGAGUGUCGUGCAGCCUCCAGGAC
505. KY620362.1/20-137 ACCUGCCUCUUA--C----GAGG-CGACACUCCACCAUGGAUCACUCCCCUGUGAGGAACUUCUGUCUUCACGCGGAAAGCGCCUAGCCAUGGCGUUAGUACGAGUGUCGUGCAGCCUCCAGGAC
506. KY283130.1/1-119 ACCCGCUCUCUA-UG----AGAG-CAACACUCCACCAUGAACCGCUCCCCUGUGAGGAACUACUGUCUUCACGCAGAAAGCGUCUAGCCAUGGCGUUAGUAUGAGUGUUGUACAGCCUCCAGGAC
507. KY620432.1/21-138 ACCUGCCUCUUA--U----GAGG-CGACACUCCACCAUGGAUCACUCCCCUGUGAGGAACUUCUGUCUUCACGCGGAAAGCGCCUAGCCAUGGCGUUAGUACGAGUGUCGUGCAGCCUCCAGGCC
508. JF735136.1/1-119 ACCUGCUCUCUA-UG----AGAG-CAACACUCCACCAUGAACCGCUCCCCUGUGAGGAACUACUGUCUUCACGCAGAAAGCGUCUAGCCAUGGCGUUAGUAUGAGUGUUGUGCAGCCUCCAGGAC
509. JF735132.1/1-119 ACCUGCUCUCUA-UG----AGAG-CAACACUCCACCAUGAACCGCUCCCCUGUGAGGAACUACUGUCUUCACGCAGAAAGCGUCUAGCCAUGGCGUUAGUAUGAGUGUUGUGCAGCCUCCAGGAC
510. JF735131.1/1-119 ACCUGCUCUCUA-UG----AGAG-CAACACUCCACCAUGAACCGCUCCCCUGUGAGGAACUACUGUCUUCACGCAGAAAGCGUCUAGCCAUGGCGUUAGUAUGAGUGUUGUGCAGCCUCCAGGAC
511. JF735130.1/1-119 ACCUGCUCUCUA-UG----AGAG-CAACACUCCACCAUGAACCGCUCCCCUGUGAGGAACUACUGUCUUCACGCAGAAAGCGUCUAGCCAUGGCGUUAGUAUGAGUGUUGUGCAGCCUCCAGGAC
512. JF735129.1/1-119 ACCUGCUCUCUA-UG----AGAG-CAACACUCCACCAUGAACCGCUCCCCUGUGAGGAACUACUGUCUUCACGCAGAAAGCGUCUAGCCAUGGCGUUAGUAUGAGUGUUGUGCAGCCUCCAGGAC
513. JF735127.1/1-119 ACCUGCUCUCUA-UG----AGAG-CAACACUCCACCAUGAACCGCUCCCCUGUGAGGAACUACUGUCUUCACGCAGAAAGCGUCUAGCCAUGGCGUUAGUAUGAGUGUUGUGCAGCCUCCAGGAC
514. FJ462438.1/1-119 ACCUGCUCUCUA-UG----AGAG-CAACACUCCACCAUGAACCGCUCCCCUGUGAGGAACUACUGUCUUCACGCAGAAAGCGUCUAGCCAUGGCGUUAGUAUGAGUGUUGUGCAGCCUCCAGGAC
515. FJ462434.1/1-119 ACCUGCUCUCUA-UG----AGAG-CAACACUCCACCAUGAACCGCUCCCCUGUGAGGAACUACUGUCUUCACGCAGAAAGCGUCUAGCCAUGGCGUUAGUAUGAGUGUUGUGCAGCCUCCAGGAC
516. FJ462432.1/1-119 ACCUGCUCUCUA-UG----AGAG-CAACACUCCACCAUGAACCGCUCCCCUGUGAGGAACUACUGUCUUCACGCAGAAAGCGUCUAGCCAUGGCGUUAGUAUGAGUGUUGUGCAGCCUCCAGGAC
517. KU180717.1/1-119 CCAUCUCCCUAA-UG----GGGG-CGACACUCCACCAUGAAUCACUCCCCUGUGAGGAACUACUGUCUUCACGCAGAAAGCGUCUAGCCAUGGCGUUAGUAUGAGUGUCGUGCAGCCUCCAGGAC
518. KJ678765.1/1-117 GCCAGCCCCU-A-AU----GGGG-CGACACUCCACCAUU-AUCACUCCCCUGUGAGGAACUACUGUCUUCACGCGGAAAGCGUCUAGCCAUGGCGUUAGUAUGAGUGUCGUACAGCCUCCAGGCC
519. KM349851.1/1-120 GCCCGCCCCCUG-AU----GGGGgCGACACUCCGCCAUGAAUCACUCCCCUGUGAGGAACUACUAUCUUCACGCAGAAAGCGUCUAGCCAUGGCGUUAGUAUGAGUGUCGUACAGCCUCCAGGCC
520. JQ717259.1/3-120 UCCUGCCUCUUA--C----GAGG-CGACACUCCACCAUGAAUCACUCCCCUGUGAGGAACUUCUGUCUUCACGCGGAAAGCGUCUAGCCAUGGCGUUAGUACGAGUGUCGUGCAGCCUCCAGGAC
521. KY620320.1/1-117 --CCGCCCCUAA-AA----GGGG-CGACACUCCGCCAUGAACCACUCCCCUGUGAGGAACUACUGUCUUCACGCAGAAAGCGUCUAGCCAUGGCGUUAGUAUGAGUGUCGUACAGCCUCCAGGCC
522. FJ462433.1/1-119 ACCUGCUCUCUA-UG----AGAG-CAACACUCCACCAUGAACCGCUCCCCUGUGAGGAACUACUGUCUUCACGCAGAAAGCGUCUAGCCAUGGCGCUAGUAUGAGUGUUGUGCAGCCUCCAGGAC
523. FJ462439.1/1-119 ACCUGCUCUCUA-UG----AGAG-CAACACUCCACCAUGAACCGCUCCCCUGUGAGGAACUACUGUCUUCACGCAGAAAGCGUCUAGCCAUGGCGUUAGUAUGAGUGUUGURCAGCCUCCAGGAY
524. JQ717258.1/3-120 UCCUGCCUCUUA--C----GAGG-CGACACUCCACCAUGGAUCACUCCCCUGUGAGGAACUUCUGUCUUCACGCGGAAAGCGCCUAGCCAUGGCGUUAGUACGAGUGUCGUGCAGCCUCCAGGAC
525. JQ717256.1/3-120 UCCUGCCUCUUA--C----GAGG-CGACACUCCACCAUGGAUCACUCCCCUGUGAGGAACUUCUGUCUUCACGCGGAAAGCGCCUAGCCAUGGCGUUAGUACGAGUGUCGUGCAGCCUCCAGGAC
526. KY620322.1/22-140 ACCUGCUCUUUU-AG----GGGG-CGACACUCCACCAUGGAUCACUCCCCUGUGAGGAACUUCUGUCUUCACGCGGAAAGCGCCUAGCCAUGGCGUUAGUACGAGUGUCGUGCAGCCUCCAGGAC
527. KJ470618.1/1-118 ACCUGCCUCUU--UC----GAGG-CGACACUCCACCAUGGAUCACUCCCCUGUGAGGAACUUCUGUCUUCACGCGGAAAGCGCCUAGCCAUGGCGUUAGUACGAGUGUCGUGCAGCCUCCAGGAC
528. KJ470615.1/1-118 ACCUGCCUCUU--UC----GAGG-CGACACUCCACCAUGGAUCACUCCCCUGUGAGGAACUUCUGUCUUCACGCGGAAAGCGCCUAGCCAUGGCGUUAGUACGAGUGUCGUGCAGCCUCCAGGAC
529. KJ470614.1/1-118 ACCUGCCUCUU--UC----GAGG-CGACACUCCACCAUGGAUCACUCCCCUGUGAGGAACUUCUGUCUUCACGCGGAAAGCGCCUAGCCAUGGCGUUAGUACGAGUGUCGUGCAGCCUCCAGGAC
530. KJ470613.1/1-118 ACCUGCCUCUU--UC----GAGG-CGACACUCCACCAUGGAUCACUCCCCUGUGAGGAACUUCUGUCUUCACGCGGAAAGCGCCUAGCCAUGGCGUUAGUACGAGUGUCGUGCAGCCUCCAGGAC
531. KJ470612.1/1-118 ACCUGCCUCUU--UC----GAGG-CGACACUCCACCAUGGAUCACUCCCCUGUGAGGAACUUCUGUCUUCACGCGGAAAGCGCCUAGCCAUGGCGUUAGUACGAGUGUCGUGCAGCCUCCAGGAC
532. KY620877.1/21-138 ACCUGCCUCUU--UC----GAGG-CGACACUCCACCAUGGAUCACUCCCCUGUGAGGAACUUCUGUCUUCACGCGGAAAGCGCCUAGCCAUGGCGUUAGUACGAGUGUCGUGCAGCCUCCAGGAC
533. AB795432.2/1-119 ACCUGCUCUCUA-UG----AGAG-CAACACUCCACCAUGAACCGCUCCCCUGUGAGGAACUACUGUCUUCACGCAGAAAGCGUCUAGCCAUGGCGUUAGUAUGAGUGUUGUACAGCCUCCAGGAU
534. KY620821.1/28-145 ACCUGCCUCUUA--C----GAGG-CGACACUCCACCAUGAAUCACUCCCCUGUGAGGAACUUCUGUCUUCACGCGGAAAGCGUCUAGCCAUGGCGUUAGUACGAGUGUCGUGCAGCCUCCAGGCC
535. KY620714.1/23-139 ACCUGCCUCCUG-------GAGG-CGACACUCCACCAUGGAUCACUCCCCUGUGAGGAACUUCUGUCUUCACGCGGAAAGCGCCUAGCCAUGGCGUUAGUACGAGUGUCGUGCAGCCUCCAGGAC
536. JF735121.1/1-118 ACCUGCCUCUU--UC----GAGG-CGACACUCCACCAUGAAUCACUCCCCUGUGAGGAACUACUGUCUUCACGCGGAAAGCGUCUAGCCAUGGCGUUAGUACGAGUGUCGAACAGCCUCCAGGCC
537. KY620589.1/26-143 ACCUGCCUCUUA--C----GAGG-CGACACUCCACCAUGGAUCACUCCCCUGUGAGGAACUUCUGUCUUCACGCGGAAAGCGCCUAGCCAUGGCGUUAGUACGAGUGUCGUACAGCCUCCAGGCC
538. KU180726.1/1-118 -CCACCCCCUGA-UG----GGGG-CGACACUCCACCAUGAAUCACUCCCCUGUGAGGAACUACUGUCUUCACGCAGAAAGCGUCUAGCCAUGGCGUUAGUAUGAGUGUCGUACAGCCUCCAGGAC
539. KY620393.1/22-139 ACCUGCCUCUUA--C----GAGG-CGACACUCCACCAUGGAUCACUCCCCUGUGAGGAACUUCUGUCUUCACGCGGAAAGCGUCUAGCCAUGGCGUUAGUACGAGUGUCGUGCAGCCUCCAGGCC
540. KY620520.1/21-138 CCCUGCCUCUUA--C----GAGG-CGACACUCCACCAUGGAUCACUCCCCUGUGAGGAACUUCUGUCUUCACGCGGAAAGCGCCUAGCCAUGGCGUUAGUACGAGUGUCGUGCAGCCUCCAGGAC
541. KY620500.1/15-132 CCCUGCCUCUUA--C----GAGG-CGACACUCCACCAUGGAUCACUCCCCUGUGAGGAACUUCUGUCUUCACGCGGAAAGCGCCUAGCCAUGGCGUUAGUACGAGUGUCGUGCAGCCUCCAGGAC
542. KY620477.1/22-139 CCCUGCCUCUUA--C----GAGG-CGACACUCCACCAUGGAUCACUCCCCUGUGAGGAACUUCUGUCUUCACGCGGAAAGCGCCUAGCCAUGGCGUUAGUACGAGUGUCGUGCAGCCUCCAGGAC
543. JX183550.1/1-117 GCCAGCCCCU-A-AU----GGGG-CGACACUCCACCAUA-AUCACUCCCCUGUGAGGAACUACUGUCUUCACGCAGAAAGCGUCUAGCCAUGGCGUUAGUAUGAGUGUCGUGCAACCUCCAGGAC
544. KY620481.1/14-131 ACCUGCCUCUUA--C----GAGG-CGACACUCCACCAUAGAUCACUCCCCUGUGAGGAACUUCUGUCUUCACGCGGAAAGCGCCUAGCCAUGGCGUUAGUACGAGUGUCGUGCAGCCUCCAGGCC
545. FJ407092.1/1-118 ACCUGCCUCUUA--C----GAGG-CGACACUCCACCAUGGAUCACUCCCCUGUGAGGAACUUCUGUCUUCACGCGGAAAGCGCCUAGCCAUGGCGUUAGUACGAGUGUCGUGCAGCCUCCAGGCC
546. KY620832.1/14-131 ACCUGCCUCUUA--C----GAGG-CGACACUCCACCAUGGAUCACUCCCCUGUGAGGAACUUCUGUCUUCACGCGGAAAGCGCCUAGCCAUGGCGUUAGUACGAGUGUCGUGCAGCCUCCAGGCC
547. KY620829.1/20-137 ACCUGCCUCUUA--C----GAGG-CGACACUCCACCAUGGAUCACUCCCCUGUGAGGAACUUCUGUCUUCACGCGGAAAGCGCCUAGCCAUGGCGUUAGUACGAGUGUCGUGCAGCCUCCAGGCC
548. KY620775.1/15-132 ACCUGCCUCUUA--C----GAGG-CGACACUCCACCAUGGAUCACUCCCCUGUGAGGAACUUCUGUCUUCACGCGGAAAGCGCCUAGCCAUGGCGUUAGUACGAGUGUCGUGCAGCCUCCAGGCC
549. KY620721.1/21-138 ACCUGCCUCUUA--C----GAGG-CGACACUCCACCAUGGAUCACUCCCCUGUGAGGAACUUCUGUCUUCACGCGGAAAGCGCCUAGCCAUGGCGUUAGUACGAGUGUCGUGCAGCCUCCAGGCC
550. KY620687.1/7-124 ACCUGCCUCUUA--C----GAGG-CGACACUCCACCAUGGAUCACUCCCCUGUGAGGAACUUCUGUCUUCACGCGGAAAGCGCCUAGCCAUGGCGUUAGUACGAGUGUCGUGCAGCCUCCAGGCC
551. KY620545.1/16-133 ACCUGCCUCUUA--C----GAGG-CGACACUCCACCAUGGAUCACUCCCCUGUGAGGAACUUCUGUCUUCACGCGGAAAGCGCCUAGCCAUGGCGUUAGUACGAGUGUCGUGCAGCCUCCAGGCC
552. KY620518.1/14-131 ACCUGCCUCUUA--C----GAGG-CGACACUCCACCAUGGAUCACUCCCCUGUGAGGAACUUCUGUCUUCACGCGGAAAGCGCCUAGCCAUGGCGUUAGUACGAGUGUCGUGCAGCCUCCAGGCC
553. KY620398.1/11-128 ACCUGCCUCUUA--C----GAGG-CGACACUCCACCAUGGAUCACUCCCCUGUGAGGAACUUCUGUCUUCACGCGGAAAGCGCCUAGCCAUGGCGUUAGUACGAGUGUCGUGCAGCCUCCAGGCC
554. KY620363.1/15-132 ACCUGCCUCUUA--C----GAGG-CGACACUCCACCAUGGAUCACUCCCCUGUGAGGAACUUCUGUCUUCACGCGGAAAGCGCCUAGCCAUGGCGUUAGUACGAGUGUCGUGCAGCCUCCAGGCC
555. AF046866.1/1-118 ACCUGCCUCUUA--C----GAGG-CGACACUCCACCAUGGAUCACUCCCCUGUGAGGAACUUCUGUCUUCACGCGGAAAGCGCCUAGCCAUGGCGUUAGUACGAGUGUCGUGCAGCCUCCAGGCC
556. KY620875.1/20-137 ACCUGCCUCUUA--C----GAGG-CGACACUCCACCAUGGAUCACUCCCCUGUGAGGAACUUCUGUCUUCACGCGGAAAGCGCCUAGCCAUGGCGUUAGUACGAGUGUCGUGCAGCCUCCAGGCC
557. KY620782.1/20-137 ACCUGCCUCUUA--C----GAGG-CGACACUCCACCAUGGAUCACUCCCCUGUGAGGAACUUCUGUCUUCACGCGGAAAGCGCCUAGCCAUGGCGUUAGUACGAGUGUCGUGCAGCCUCCAGGCC
558. KY620779.1/16-133 ACCUGCCUCUUA--C----GAGG-CGACACUCCACCAUGGAUCACUCCCCUGUGAGGAACUUCUGUCUUCACGCGGAAAGCGCCUAGCCAUGGCGUUAGUACGAGUGUCGUGCAGCCUCCAGGCC
559. KY620768.1/21-138 ACCUGCCUCUUA--C----GAGG-CGACACUCCACCAUGGAUCACUCCCCUGUGAGGAACUUCUGUCUUCACGCGGAAAGCGCCUAGCCAUGGCGUUAGUACGAGUGUCGUGCAGCCUCCAGGCC
560. KY620767.1/21-138 ACCUGCCUCUUA--C----GAGG-CGACACUCCACCAUGGAUCACUCCCCUGUGAGGAACUUCUGUCUUCACGCGGAAAGCGCCUAGCCAUGGCGUUAGUACGAGUGUCGUGCAGCCUCCAGGCC
561. KY620748.1/16-133 ACCUGCCUCUUA--C----GAGG-CGACACUCCACCAUGGAUCACUCCCCUGUGAGGAACUUCUGUCUUCACGCGGAAAGCGCCUAGCCAUGGCGUUAGUACGAGUGUCGUGCAGCCUCCAGGCC
562. KY620741.1/20-137 ACCUGCCUCUUA--C----GAGG-CGACACUCCACCAUGGAUCACUCCCCUGUGAGGAACUUCUGUCUUCACGCGGAAAGCGCCUAGCCAUGGCGUUAGUACGAGUGUCGUGCAGCCUCCAGGCC
563. KY620730.1/20-137 ACCUGCCUCUUA--C----GAGG-CGACACUCCACCAUGGAUCACUCCCCUGUGAGGAACUUCUGUCUUCACGCGGAAAGCGCCUAGCCAUGGCGUUAGUACGAGUGUCGUGCAGCCUCCAGGCC
564. KY620691.1/23-140 ACCUGCCUCUUA--C----GAGG-CGACACUCCACCAUGGAUCACUCCCCUGUGAGGAACUUCUGUCUUCACGCGGAAAGCGCCUAGCCAUGGCGUUAGUACGAGUGUCGUGCAGCCUCCAGGCC
565. KY620690.1/22-139 ACCUGCCUCUUA--C----GAGG-CGACACUCCACCAUGGAUCACUCCCCUGUGAGGAACUUCUGUCUUCACGCGGAAAGCGCCUAGCCAUGGCGUUAGUACGAGUGUCGUGCAGCCUCCAGGCC
566. KY620686.1/11-128 ACCUGCCUCUUA--C----GAGG-CGACACUCCACCAUGGAUCACUCCCCUGUGAGGAACUUCUGUCUUCACGCGGAAAGCGCCUAGCCAUGGCGUUAGUACGAGUGUCGUGCAGCCUCCAGGCC
567. KY620684.1/12-129 ACCUGCCUCUUA--C----GAGG-CGACACUCCACCAUGGAUCACUCCCCUGUGAGGAACUUCUGUCUUCACGCGGAAAGCGCCUAGCCAUGGCGUUAGUACGAGUGUCGUGCAGCCUCCAGGCC
568. KY620682.1/17-134 ACCUGCCUCUUA--C----GAGG-CGACACUCCACCAUGGAUCACUCCCCUGUGAGGAACUUCUGUCUUCACGCGGAAAGCGCCUAGCCAUGGCGUUAGUACGAGUGUCGUGCAGCCUCCAGGCC
569. KY620656.1/21-138 ACCUGCCUCUUA--C----GAGG-CGACACUCCACCAUGGAUCACUCCCCUGUGAGGAACUUCUGUCUUCACGCGGAAAGCGCCUAGCCAUGGCGUUAGUACGAGUGUCGUGCAGCCUCCAGGCC
570. KY620653.1/21-138 ACCUGCCUCUUA--C----GAGG-CGACACUCCACCAUGGAUCACUCCCCUGUGAGGAACUUCUGUCUUCACGCGGAAAGCGCCUAGCCAUGGCGUUAGUACGAGUGUCGUGCAGCCUCCAGGCC
571. KY620642.1/13-130 ACCUGCCUCUUA--C----GAGG-CGACACUCCACCAUGGAUCACUCCCCUGUGAGGAACUUCUGUCUUCACGCGGAAAGCGCCUAGCCAUGGCGUUAGUACGAGUGUCGUGCAGCCUCCAGGCC
572. KY620611.1/17-134 ACCUGCCUCUUA--C----GAGG-CGACACUCCACCAUGGAUCACUCCCCUGUGAGGAACUUCUGUCUUCACGCGGAAAGCGCCUAGCCAUGGCGUUAGUACGAGUGUCGUGCAGCCUCCAGGCC
573. KY620606.1/24-141 ACCUGCCUCUUA--C----GAGG-CGACACUCCACCAUGGAUCACUCCCCUGUGAGGAACUUCUGUCUUCACGCGGAAAGCGCCUAGCCAUGGCGUUAGUACGAGUGUCGUGCAGCCUCCAGGCC
574. KY620521.1/20-137 ACCUGCCUCUUA--C----GAGG-CGACACUCCACCAUGGAUCACUCCCCUGUGAGGAACUUCUGUCUUCACGCGGAAAGCGCCUAGCCAUGGCGUUAGUACGAGUGUCGUGCAGCCUCCAGGCC
575. KY620516.1/21-138 ACCUGCCUCUUA--C----GAGG-CGACACUCCACCAUGGAUCACUCCCCUGUGAGGAACUUCUGUCUUCACGCGGAAAGCGCCUAGCCAUGGCGUUAGUACGAGUGUCGUGCAGCCUCCAGGCC
576. KY620515.1/28-145 ACCUGCCUCUUA--C----GAGG-CGACACUCCACCAUGGAUCACUCCCCUGUGAGGAACUUCUGUCUUCACGCGGAAAGCGCCUAGCCAUGGCGUUAGUACGAGUGUCGUGCAGCCUCCAGGCC
577. KY620470.1/22-139 ACCUGCCUCUUA--C----GAGG-CGACACUCCACCAUGGAUCACUCCCCUGUGAGGAACUUCUGUCUUCACGCGGAAAGCGCCUAGCCAUGGCGUUAGUACGAGUGUCGUGCAGCCUCCAGGCC
578. KY620463.1/9-126 ACCUGCCUCUUA--C----GAGG-CGACACUCCACCAUGGAUCACUCCCCUGUGAGGAACUUCUGUCUUCACGCGGAAAGCGCCUAGCCAUGGCGUUAGUACGAGUGUCGUGCAGCCUCCAGGCC
579. KY620462.1/21-138 ACCUGCCUCUUA--C----GAGG-CGACACUCCACCAUGGAUCACUCCCCUGUGAGGAACUUCUGUCUUCACGCGGAAAGCGCCUAGCCAUGGCGUUAGUACGAGUGUCGUGCAGCCUCCAGGCC
580. KY620445.1/85-202 ACCUGCCUCUUA--C----GAGG-CGACACUCCACCAUGGAUCACUCCCCUGUGAGGAACUUCUGUCUUCACGCGGAAAGCGCCUAGCCAUGGCGUUAGUACGAGUGUCGUGCAGCCUCCAGGCC
581. KY620443.1/11-128 ACCUGCCUCUUA--C----GAGG-CGACACUCCACCAUGGAUCACUCCCCUGUGAGGAACUUCUGUCUUCACGCGGAAAGCGCCUAGCCAUGGCGUUAGUACGAGUGUCGUGCAGCCUCCAGGCC
582. KY620428.1/21-138 ACCUGCCUCUUA--C----GAGG-CGACACUCCACCAUGGAUCACUCCCCUGUGAGGAACUUCUGUCUUCACGCGGAAAGCGCCUAGCCAUGGCGUUAGUACGAGUGUCGUGCAGCCUCCAGGCC
583. KY620388.1/21-138 ACCUGCCUCUUA--C----GAGG-CGACACUCCACCAUGGAUCACUCCCCUGUGAGGAACUUCUGUCUUCACGCGGAAAGCGCCUAGCCAUGGCGUUAGUACGAGUGUCGUGCAGCCUCCAGGCC
584. KY620383.1/25-142 ACCUGCCUCUUA--C----GAGG-CGACACUCCACCAUGGAUCACUCCCCUGUGAGGAACUUCUGUCUUCACGCGGAAAGCGCCUAGCCAUGGCGUUAGUACGAGUGUCGUGCAGCCUCCAGGCC
585. DQ835768.1/1-117 GCCAGCCCCU-A-AU----GGGG-CGACACUCCACCAUG-AUCACUCCCCUGUGAGGAACUAAUGUCUUCACGCAGAAAGCGUCUAGCCAUGGCGUUAGUAUGAGUGUCGUACAGCCUCCAGGAU
586. DQ835766.1/1-117 GCCAGCCCCU-A-AU----GGGG-CGACACUCCACCAUG-AUCACUCCCCUGUGAGGAACUAAUGUCUUCACGCAGAAAGCGUCUAGCCAUGGCGUUAGUAUGAGUGUCGUACAGCCUCCAGGAU
587. KY620672.1/27-144 ACCUGCCUCUUA--C----GAGG-CGACACUCCACCAUGGAUCACUCCCCUGUGAGGAACUUCUGUCUUCACGCGGAAAGCGCCUAGCCAUGGCGUUAGUACGAGUGUUGUGCAGCCUCCAGGAC
588. JF735126.1/1-118 ACCUGCCUCUU--UC----GAGG-CGACACUCCACCAUGGAUCACUCCCCUGUGAGGAACUACUGUCUUCACGCGGAAAGCGUCUAGCCAUGGCGUUAGUACGAGUGUCGAACAGCCUCCAGGCC
589. JF735138.1/1-119 ACCUGCUCUCUA-UG----AGAG-CAACACUCCACCAUGAACCGCUCCCCUGUGAGGAACUACUGUCUUCACGCAGAAAGCGUCUAGCCAUGGCGUUAGUAUGAGUGUUGUGCAGCCUCCAGGCC
590. KJ470617.1/1-118 ACCUGCCUCCUU--C----GAGG-CGACACUCCACCAUGAAUCACUCCCCUGUGAGGAACUUCUGUCUUCACGCGGAAAGCGUCUAGCCAUGGCGUUAGUACGAGUGUCGUGCAGCCUCCAGGCC
591. DQ835767.1/1-117 GCCAGCCCCU-A-AU----GGGG-CGACACUCCACCAUG-AUCACUCCCCUGUGAGGAACUAAUGUCUUCACGCAGAAAGCGUCUAGCCAUGGCGUUAGUAUGAGUGUCGUGCAGCCUCCAGGAU
592. DQ835765.1/1-117 GCCAGCCCCU-A-AU----GGGG-CGACACUCCACCAUG-AUCACUCCCCUGUGAGGAACUAAUGUCUUCACGCAGAAAGCGUCUAGCCAUGGCGUUAGUAUGAGUGUCGUGCAGCCUCCAGGAU
593. KY620863.1/16-133 ACCUGCCUCUUC--C----GAGG-CGACACUCCACCAUGGAUCACUCCCCUGUGAGGAACUUCUGUCUUCACGCGGAAAGCGCCUAGCCAUGGCGUUAGUACGAGUGUCGUGCAGCCUCCAGGAC
594. KJ678786.1/1-118 GCCAGCCCGUUA-CA----GGGG-CGACACUCCACCAUC-AUCACUCCCCUGUGAGGAACUACUGUCUUCACGCAGAAAGCGUCUAGCCAUGGCGUUAGUAUGAGUGUCGUACAGCCUCCAGGCC
595. JN588558.1/1-118 ACCUGCCUCUUA--C----GAGG-CGACACUCCACCAUGGAUCACUCCCCUGUGAGGAACUUCUGUCUUCACGCGGAAAGCGCCUAGCCAUGGCGUUAGUACGAGUGUCGUGCAGCCUCCAGGGC
596. D63821.1/1-118 ACCUGCCUCUU--UC----GAGG-CGACACUCCACCAUGAAUCACUCCCCUGUGAGGAACUUCUGUCUUCACGCGGAAAGCGUCUAGCCAUGGCGUUAGUACGAGUGUCGUGCAGCCUCCAGGCC
597. KJ470616.1/1-118 ACCUGCCUCUU--UC----GAGG-CGACACUCCACCAUGAAUCACUCCCCUGUGAGGAACUUCUGUCUUCACGCGGAAAGCGUCUAGCCAUGGCGUUAGUACGAGUGUCGUGCAGCCUCCAGGCC
598. D49374.1/1-118 ACCUGCCUCUU--UC----GAGG-CGACACUCCACCAUGAAUCACUCCCCUGUGAGGAACUUCUGUCUUCACGCGGAAAGCGUCUAGCCAUGGCGUUAGUACGAGUGUCGUGCAGCCUCCAGGCC
599. KY620869.1/18-135 ACCUGCCUCUU--UC----GAGG-CGACACUCCACCAUGAAUCACUCCCCUGUGAGGAACUUCUGUCUUCACGCGGAAAGCGUCUAGCCAUGGCGUUAGUACGAGUGUCGUGCAGCCUCCAGGCC
600. KU180719.1/1-113 ------CCCUGA-UG----GGGG-CGACACUCCACCAUGAAUCACUCCCCUGUGAGGAACUACUGUCUUCACGCAGAAAGCGUCUAGCCAUGGCGUUAGUAUGAGUGUCGUGCAGCCUCCAGGAC
601. KY620673.1/15-132 ACCUGCCUCUUA--C----GAGG-CGACACUCCACCAUGGAUCACUCCCCUGUGAGGAACUUCUGCCCUCACGCGGAAAGCGCCUAGCCAUGGCGUUAGUACGAGUGUCGUGCAGCCUCCAGGAC
602. KY620335.1/1-118 --CCGCCCCCUG-AU----GGGGgCGACACUCCGCCAUGAAUCACUCCCCUGUGAGGAACUACUGUCUUCACGCAGAAAGCGUCUAGCCAUGGCGUUAGUAUGAGUGUCGUACAGCCUCCAGGCC
603. NC_030791.1/1-118 GCCAGCCCCUUA--C----GGGG-CGACACUCCGCCAUGAAUCACUCCCCUGCGAGGAACCACUGUCCUCACGCAGAAAGCGUCUAGCCAUGACGUUAGUAUGAGUGUCGUACAGCCUCCAGGAC
604. EF108306.2/1-118 GCCAGCCCCUUA--C----GGGG-CGACACUCCGCCAUGAAUCACUCCCCUGCGAGGAACCACUGUCCUCACGCAGAAAGCGUCUAGCCAUGACGUUAGUAUGAGUGUCGUACAGCCUCCAGGAC
605. KY620342.1/1-115 ----GCCCCUGG-UG----GGGG-CGACACUCCGCCAUGAAUCACUCCCCUGUGAGGAACUACUGUCUUCACGCAGAAAGCGUCUAGCCAUGGCGUUAGUAUGAGUGUCGUGCAGCCUCCAGGUC
606. KY620373.1/15-132 ACCUGCCUCUUA--U----GAGG-CAACACUCCACCAUGGAUCACUCCCCUGUGAGGAACUUCUGUCUUCACGCGGAAAGCGCCUAGCCAUGGCGUUAGUACGAGUGUUGUGCAGCCUCCAGGAC
607. JF735123.1/1-118 ACCUGCCUCUU--UC----GAGG-CGACACUCCACCAUAGAUCACUCCCCUGUGAGGAACUUCUGUCUUCACGCGGAAAGCGUCUAGCCAUGGCGUUAGUACGAGUGUCGUGCAGCCUCCAGGCC
608. KY620879.1/23-140 ACCUGCCUCUU--UC----GAGG-CGACACUCCACCAUGGAUCACUCCCCUGUGAGGAACUUCUGUCUUCACGCGGAAAGCGUCUAGCCAUGGCGUUAGUACGAGUGUCGUGCAGCCUCCAGGCC
609. JF735125.1/1-118 ACCUGCCUCUU--UC----GAGG-CGACACUCCACCAUGGAUCACUCCCCUGUGAGGAACUUCUGUCUUCACGCGGAAAGCGCCUAGCCAUGGCGUUAGUACGAGUGUCGUGCAGCCUCCAGGCC
610. KM587623.1/1-118 ACCUGCCUCUU--UC----GAGG-CGACACUCCACCAUGGAUCACUCCCCUGUGAGGAACUUCUGUCUUCACGCGGAAAGCGCCUAGCCAUGGCGUUAGUACGAGUGUCGUGCAGCCUCCAGGCC
611. KY620359.1/9-128 GGCGGCCUCGAG-GU----GGGGgCGACACUCCGCCAUGAAUCACUCCCCUGUGAGGAACUACUGUCUUCACGCAGAAAGCGUCUAGCCAUGGCGUUAGUAUGAGUGUCGUACAGCCUCCAGGCC
612. JX183558.1/1-117 GCCAGCCCCU-A-AU----GGGG-SGACACUCCACCAUG-AUCACUCCCCUGUGAGGAACUACUGUCUUCACGCGGAAAGCGUCUAGCCAUGGCGUUAGUACGAGUGUCGAACAGCCUCCAGGCC
613. KY620575.1/1-119 CCUGCCCCCUGA-UG----GGGG-CGACACUCCGCCAUGAAUCACUCCCCUGUGAGGAACUUCUGUCUUCACGCGGAAAGCGCCUAGCCAUGGCGUUAGUACGAGUGUCGUGCAGCCUCCAGGAC
614. KY620645.1/19-136 ACCUGCCUCUUC--C----GGGG-CGACACUCCACCAUGGAUCACUCCCCUGUGAGGAACUUCUGUCUUCACGCGGAAAGCGCCUAGCCAUGGCGUUAGUACGAGUGUCGUGCAGCCUCCAGGAC
615. KY620592.1/13-130 ACCUGCCUCUUA--C----GAGG-CAACACUCCACCAUGGAUCACUCCCCUGUGAGGAACUUCUGUCUUCACGCGGAAAGCGCCUAGCCAUGGCGUUAGUACGAGUGUUGUGCAGCCUCCAGGAC
616. KY620344.1/11-129 ACGAUCUCCUGA-UG----GGGG-CGACACUCCGCCAUGAAUCACUCCCCUGUGAGGAACUACUGUCUUCACGCAGAAAGCGUCUAGCCAUGGCGUUAGUAUGAGUGUCGUACAGCCUCCAGGCC
617. KY620509.1/21-141 ACCUGCCUCUGG-A-ggc-GAGG-CGACACUCCACCAUGGAUCACUCCCCUGUGAGGAACUUCUGUCUUCACGCGGAAAGCGCCUAGCCAUGGCGUUAGUACGAGUGUCGUGCAGCCUCCAGGAC
618. KU180710.1/1-113 ------CCCUGA-UG----GGGG-CAACACUCCACCAUGAAUCACUCCCCUGUGAGGAACUACUGUCUUCACGCAGAAAGCGUCUAGCCAUGGCGUUAGUAUGAGUGUCGUGCAGCCUCCAGGAC
619. KJ470619.1/1-118 ACCUGCCUCUU--UC----GAGG-CGACACUSCACCAUGGAUCACUCCCCUGUGAGGAACUUCUGUCUUCACGCGGAAAGCGCCUAGCCAUGGCGUUAGUACGAGUGUCGUGCAGCCUCCAGGCC
620. EF424625.1/1-117 GCCAGCCCCU-A-AU----GGGG-CGACACUCCACCAUG-AUCACUCCCCUGUGAGGAACUAUUGUCUUCACGCAGAAAGCGUCUAGCCAUGGCGUUAGUAUGAGCGUUGUACAACCUCCAGGAC
621. JF735122.1/1-118 ACCUGCCUCUU--UC----GAGG-CGACACUCCACCAUGAACCACUCCCCUGUGAGGAACUUCUGUCUUCACGCGGAAAGCGUCUAGCCAUGGCGUUAGUACGAGUGUCGUGCAGCCUCCAGGCC
622. KY620593.1/18-135 ACCUGCCUCUUA--C----GAGG-CGACACUCCACCAUAGAUCACUCCCCUGUGAGGAACUUCUGUCUUCACGCGGAAAGCGCCUAGCCAUGGUGUUAGUACGAGUGUCGUACAGCCUCCAGGAC
623. KY620880.1/6-124 CGAUCUCCCUGA-UG----GGGG-CGACACUCCGCCAUGAAUCACUCCCCUGUGAGGAACUACUGUCUUCACGCAGAAAGCGUCUAGCCAUGGCGUUAGUAUGAGUGUCGUACAGCCUCCAGGCC
624. KY620345.1/5-123 CGAUCUCCCUGA-UG----GGGG-CGACACUCCGCCAUGAAUCACUCCCCUGUGAGGAACUACUGUCUUCACGCAGAAAGCGUCUAGCCAUGGCGUUAGUAUGAGUGUCGUACAGCCUCCAGGCC
625. KY620357.1/10-128 CGCUCUCCCGGG-AG----GGGG-CGACACUCCGCCAUGAAUCACUCCCCUGUGAGGAACUACUGUCUUCACGCAGAAAGCGUCUAGCCAUGGCGUUAGUAUGAGUGUCGUACAGCCUCCAGGCC
626. KY620787.1/20-137 ACCUGCCUUUUG-C-----GAGG-CGACACUCCACCAUGGAUCACUCCCCUGUGAGGAACUUCUGUCUUCACGCGGAAAGCGCCUAGCCAUGGCGUUAGUACGAGUGUCGUGCAGCCUCCAGGAC
627. KY620415.1/23-140 ACCUGCCUCUUA--C----GAGG-CGACACUCCACCAUGGAUCACUCCCCUGUGAGGAACUUCUGUCUUCACGCGGAAAGCGCCUAGCCAUGACGUUAGUACGAGUGUCGUGCAGCCUCCAGGAC
628. KY620355.1/6-124 GGGGGGGCCUUA-UG----GGGG-CGACACUCCACCAUGAAUCACUCCCCUGUGAGGAACUACUGUCUUCACGCAGAAAGCGUCUAGCCAUGGCGUUAGUAUGAGUGUCGUACAGCCUCCAGGCC
629. KY620788.1/8-125 ACCUGCCUCUUA--C----GAGA-CGACACUCCACCAUGGAUCACUCCCCUGUGAGGAACUUCUGUCUUCACGCGGAAAGCGCCUAGCCAUGGCGUUAGUACGAGUGUCGUGCAGCCUCCAGGAC
630. KU180722.1/1-117 -CCUGCCUCUUA--C----GAGG-CGACACUCCACCAUGGAUCACUCCCCUGUGAGGAACUACUGUCUUCACGCAGAAAGCGUCUAGCCAUGGCGUUAGUAUGAGUGUCGUACAGCCUCCAGGCC
631. KY620360.1/2-118 GA--UCCCCUGA-UG----GGGG-CGACACUCCGCCAUGAAUCACUCCCCUGUGAGGAACUACUGUCUUCACGCAGAAAGCGUCUAGCCAUGGCGUUAGUAUGAGUGUCGUACAGCCUCCAGGCC
632. KY620855.1/4-119 GGCUGCCUU----AU----GAGG-CGACACUCCACCAUGGAUCACUCCCCUGUGAGGAACUUCUGUCUUCACGCGGAAAGCGCCUAGCCAUGGCGUUAGUACGAGUGUCGUGCAGCCUCCAGGAC
633. KY620587.1/4-119 GGCUGCCUU----AU----GAGG-CGACACUCCACCAUGGAUCACUCCCCUGUGAGGAACUUCUGUCUUCACGCGGAAAGCGCCUAGCCAUGGCGUUAGUACGAGUGUCGUGCAGCCUCCAGGAC
634. JF735124.1/1-118 ACCUGCCUCUU--UC----GAGG-CGACACUCCACCAUGGAUCACUCCCCUGUGAGGAACUUCUGUCUUCACGCGGAAAGCGUCUAGCCAUGGCGUUAGUACGAGCGUCGUGCAGCCUCCAGGAC
635. KY620716.1/24-139 GGAGGCCUCA----C----GAGG-CGACACUCCACCAUGGAUCACUCCCCUGUGAGGAACUUCUGUCUUCACGCGGAAAGCGCCUAGCCAUGGCGUUAGUACGAGUGUCGUGCAGCCUCCAGGAC
636. KY620643.1/20-137 ACCUGGCUCUC--CC----GAGG-CGACACUCCACCAUGGAUCACUCCCCUGUGAGGAACUUCUGUCUUCACGCGGAAAGCGCCUAGCCAUGGCGUUAGUACGAGUGUCGUGCAGCCUCCAGGAC
637. JF343789.1/43-161 AAGUUUCUCUGA-AC----GUGU-AGACACUCCGCCAUGAAUCACUCCCCUGUGAGGAACUACUGUCUUCACGCAGAAAGCGUCUAGCCAUGGCGUUAGUAUGAGUGUCGUACAGCCUCCAGGCC
638. KY620513.1/14-134 UCCUGUCUCUGG-A-ggc-GAGG-CGACACUCCACCAUGGAUCACUCCCCUGUGAGGAACUUCUGUCUUCACGCGGAAAGCGCCUAGCCAUGGCGUUAGUACGAGUGUCGUGCAGCCUCCAGGAC
639. KY620535.1/16-129 GGCAACCUC----------GAGG-CGACACUCCACCAUGGAUCACUCCCCUGUGAGGAACUUCUGUCUUCACGCGGAAAGCGCCUAGCCAUGGCGUUAGUACGAGUGUCGUGCAGCCUCCAGGAC
640. JF343791.1/2-120 CUGAGGUCCUUU-AA----GGAC-CGACACUCCGCCAUGAAUCACUCCCCUGUGAGGAACUACUGUCUUCACGCAGAAAGCGUCUAGCCAUGGCGUUAGUAUGAGUGUCGUACAGCCUCCAGGCC
641. KY620528.1/1-117 -CCUGCCUCUUA--C----GAGG-CGACACUCCACCAUGGAUCACUCCCCUGUGAGGAACUUCUGUCUUCACGCGGAAAGCGCCUAGCCAUGGCGUUAGUACGAGUGUCGUGCAGCCUCCAGGAC
642. KY620489.1/22-139 ACCUGCCGUCCA-C-----GAGG-CGACACUCCACCAUGGAUCACUCCCCUGUGAGGAACUUCUGUCUUCACGCGGAAAGCGCCUAGCCAUGGCGUUAGUACGAGUGUCGUGCAGCCUCCAGGAC
643. KY620713.1/1-116 --CUGCCUCUUA--C----GAGG-CGACACUCCACCAUGGAUCACUCCCCUGUGAGGAACUUCUGUCUUCACGCGGAAAGCGCCUAGCCAUGGCGUUAGUACGAGUGUCGUGCAGCCUCCAGGAC
644. KY620723.1/11-129 GGGGGCCUGCUU-AC----GAGG-CGACACUCCACCAUGGAUCACUCCCCUGUGAGGAACUUCUGUCUUCACGCGGAAAGCGCCUAGCCAUGGCGUUAGUACGAGUGUCGUGCAGCCUCCAGGCC
645. DQ278894.1/4-121 GCCAGCCCCUUA-AC----GGGG-CGACACUCCACCAUA-AUCACUCCCCCUUGAGGAACUAAUGUCUUCACGCAGAAAGCGUCUAGCCAUGGCGUUAGUAUGAGUGUCGUACAGCCUCCAGGAU
646. KY620356.1/6-124 CCGGGGGCCUGA-UG----GGGG-CGACACUCCGCCAUGAAUCACUCCCCUGUGAGGAACUACUGUCUUCACGCAGAAAGCGUCUAGCCAUGGCGUUAGUAUGAGUGUCGUACAGCCUCCAGGCC
647. KU180715.1/1-119 ACCUGCCCCUGA-UG----AGAG-CAACACUCCACCAUGAACCGCUCCCCUGUGAGGAACUACUGUCUUCACGCAGAAAGCGUCUAGCCAUGGCGUUAGUAUGAGUGUUGUACAGCCUCCAGGAC
648. KY620851.1/20-137 ACCUGACCCUUA-U-----GAGG-CGACACUCCACCAUGGAUCACUCCCCUGUGAGGAACUUCUGUCUUCACGCGGAAAGCGCCUAGCCAUGGCGUUAGUACGAGUGUCGUGCAGCCUCCAGGCC
649. KY620627.1/13-126 ACCUGCCU-------g-g--AGG-CGACACUCCACCAUGGAUCACUCCCCUGUGAGGAACUUCUGUCUUCACGCGGAAAGCGCCUAGCCAUGGCGUUAGUACGAGUGUCGUGCAGCCUCCAGGAC
650. L02836.1/1-109 ----------AU-UG----GGGG-CGACACUCCACCAUAGAUCACUCCCCUGUGAGGAACUACUGUCUUCACGCAGAAAGCGUCUAGCCAUGGCGUUAGUAUGAGUGUCGUGCAGCCUCCAGGAC
651. KY620331.1/18-137 GGGGGGCCUGGA-GG----AGGGgCGACACUCCGCCAUGAAUCACUCCCCUGUGAGGAACUACUGUCUUCACGCAGAAAGCGUCUAGCCAUGGCGUUAGUAUGAGUGUCGUACAGCCUCCAGGCC
652. KY620719.1/8-121 GGGGGCCUC----------GAGG-CGACACUCCACCAUGGAUCACUCCCCUGUGAGGAACUUCUGUCUUCACGCGGAAAGCGCCUAGCCAUGGCGUUAGUACGAGUGUCGUGCAGCCUCCAGGCC
653. KY620727.1/4-122 UCCGACCUGGAG-GC----UAGG-CGACACUCCACCAUGGAUCACUCCCCUGUGAGGAACUUCUGUCUUCACGCGGAAAGCGCCUAGCCAUGGCGUUAGUACGAGUGUCGUGCAGCCUCCAGGAC
654. KY620314.1/1-118 -CCGAUCUCUAA-AA----GGGG-CGACACUCCGCCAUGAAUCACUCCCCUGUGAGGAACUACUGUCUUCACGCAGAAAGCGUCUAGCCAUGGCGUUAGUAUGAGUGUCGUACAGCCUCCAGGCC
655. KY620675.1/67-185 GGGGGCCUGGAG-GC----UAGG-CGACACUCCACCAUAGAUCACUCCCCUGUGAGGAACUUCUGUCUUCACGCGGAAAGCGUCUAGCCAUGGCGUUAGUACGAGUGUCGUGCAGCCUCCAGGCC
656. KY620737.1/1-117 -CCUGCCUCUUA--C----GAGG-CGACACUCCACCACGGAUCACUCCCCUGUGAGGAACUUCUGUCUUCACGCGGAAAGCGCCUAGCCAUGGCGUUAGUACGAGUGUCGUGCAGCCUCCAGGAC
657. KY620354.1/11-127 ACUAACGCCAUG-------GGGG-CGACACUCCGCCAUGAAUCACUCCCCUGUGAGGAACUACUGUCUUCACGCAGAAAGCGUCUAGCCAUGGCGUUAGUAUGAGUGUCGUACAGCCUCCAGGCC
658. KY620618.1/17-130 GCCGAUCUC----------GAGG-CGACACUCCACCAUGGAUCACUCCCCUGUGAGGAACUUCUGUCUUCACGCGGAAAGCGCCUAGCCAUGGCGUUAGUACGAGUGUCGUGCAGCCUCCAGGAC
659. KY620555.1/6-124 GGGGGCCUGGAG-GC----UGGG-CGACACUCCACCAUGGAUCACUCCCCUGUGAGGAACUUCUGUCUUCACGCGGAAAGCGCCUAGCCAUGGCGUUAGUACGAGUGUCGUGCAGCCUCCAGGCC
660. KY620542.1/6-124 UGCUGGACCCCA-GU----GACG-CGACACUCCACCAUGGAUCACUCCCCUGUGAGGAACUUCUGUCUUCACGCGGAAAGCGCCUAGCCAUGGCGUUAGUACGAGUGUCGUGCAGCCUCCAGGAC
661. KY620874.1/9-126 GGGAUCCUCUC--UC----GAGG-CGACACUCCACCAUGAAUCACUCCCCUGUGAGGAACUUCUGUCUUCACGCGGAAAGCGUCUAGCCAUGGCGUUAGUACGAGUGUCGUGCAGCCUCCAGGCC
662. KY620507.1/4-117 UCCGAUCUC----------GAGG-CGACACUCCACCAUGGAUCACUCCCCUGUGAGGAACUUCUGUCUUCACGCGGAAAGCGCCUAGCCAUGGCGUUAGUACGAGUGUCGUGCAGCCUCCAGGAC
663. KY620397.1/4-117 UCCGAUCUC----------GAGG-CGACACUCCACCAUGGAUCACUCCCCUGUGAGGAACUUCUGUCUUCACGCGGAAAGCGCCUAGCCAUGGCGUUAGUACGAGUGUCGUGCAGCCUCCAGGAC
664. KY620862.1/2-117 GCCC--CUCUUA--C----GAGG-CGACACUCCACCAUGGAUCACUCCCCUGUGAGGAACUUCUGUCUUCACGCGGAAAGCGCCUAGCCAUGGCGUUAGUACGAGUGUCGUGCAGCCUCCAGGAC
665. KY620433.1/9-123 UCCUGCUUG--------c-GAGG-CGACACUCCACCAUGGAUCACUCCCCUGUGAGGAACUUCUGUCUUCACGCGGAAAGCGCCUAGCCAUGGCGUUAGUACGAGUGUCGUGCAGCCUCCAGGAC
666. KY620705.1/21-140 GGGAGUCCUGGA---ggc-UGGG-CGACACUCCACCAUGGAUCACUCCCCUGUGAGGAACUUCUGUCUUCACGCGGAAAGCGCCUAGCCAUGGCGUUAGUACGAGUGUCGUGCAGCCUCCAGGAC
667. KY620644.1/2-116 UGCCGCUUA-----C----GAGG-CGACACUCCACCAUGGAUCACUCCCCUGUGAGGAACUUCUGUCUUCACGCGGAAAGCGCCUAGCCAUGGCGUUAGUACGAGUGUCGUGCAGCCUCCAGGAC
668. KY620750.1/31-149 GGAGUCCUGGAG-GC----UAGG-CGACACUCCACCAUGAAUCACUCCCCUGUGAGGAACUUCUGUCUUCACGCGGAAAGCGUCUAGCCAUGGCGUUAGUACGAGUGUCGUGCAGCCUCCAGGAC
669. KY620870.1/165-278 UAACGCCAC----------GAGG-CGACACUCCACCAUGAAUCACUCCCCUGUGAGGAACUUCUGUCUUCACGCGGAAAGCGUCUAGCCAUGGCGUUAGUACGAGUGUCGUGCAGCCUCCAGGCC
670. KY620860.1/24-141 AUCUGAUCCUUA--C----GAGG-CGACACUCCACCAUGGAUCACUCCCCUGUGAGGAACUUCUGUCUUCACGCGGAAAGCGCCUAGCCAUGGCGUUAGUACGAGUGUCGUGCAGCCUCCAGGCC
671. KY620316.1/7-125 GGGGGCCUGGAG-GC----UGGA-CGACACUCCGCCAUGAAUCACUCCCCUGUGAGGAACUACUGUCUUCACGCAGAAAGCGUCUAGCCAUGGCGUUAGUAUGAGUGUCGUACAGCCUCCAGGCC
672. KY620400.1/3-116 UCCGAUCUC----------GAGG-CGACACUCCACCAUGGAUCACUCCCCUGUGAGGAACUUCUGUCUUCACGCGGAAAGCGCCUAGCCAUGGCGUUAGUACGAGUGUCGUGCAGCCUCCAGGCC
673. KU180713.1/1-118 -CCUGAUCUCUA-UG----AGAG-CAACACUCCACCAUGAACCGCUCCCCUGUGAGGAACUACUGUCUUCACGCAGAAAGCGUCUAGCCAUGGCGUUAGUAUGAGUGUUGUACAGCCUCCAGGAC
674. KY620579.1/4-121 GGCUGCACGACA--C----GAGG-CGACACUCCACCAUGGAUCACUCCCCUGUGAGGAACUUCUGUCUUCACGCGGAAAGCGCCUAGCCAUGGCGUUAGUACGAGUGUCGUGCAGCCUCCAGGAC
675. JF343792.1/35-154 GUCUAGCCAUGGcGU----UAGC-CGACACUCCGCCAUGAAUCACUCCCCUGUGAGGAACUACUGUCUUCACGCAGAAAGCGUCUAGCCAUGGCGUUAGUAUGAGUGUCGUACAGCCUCCAGGCC
676. KY620872.1/13-127 GCCUGGGGGC---------CCCC-CGACACUCCACCAUGAACCACUCCCCUGUGAGGAACUUCUGUCUUCACGCGGAAAGCGUCUAGCCAUGGCGUUAGUACGAGUGUCGUGCAGCCUCCAGGCC
677. KY620456.1/29-145 UAACGCCAUGGC-U------AGG-CGACACUCCACCAUGGAUCACUCCCCUGUGAGGAACUUCUGUCUUCACGCGGAAAGCGCCUAGCCAUGGCGUUAGUACGAGUGUCGUGCAGCCUCCAGGAC
678. KY620756.1/24-137 GGGGUCCUC----------GAGG-CGACACUCCACCAUGGAUCACUCCCCUGUGAGGAACUUCUGUCUUCACGCGGAAAGCGCCUAGCCAUGGCGUUAGUACGAGUGUCGUGCAGCCUCCAGGAC
679. KY620622.1/2-121 GGGGGUCCUGGA---ggc-UGGG-CGACACUCCACCAUGGAUCACUCCCCUGUGAGGAACUUCUGUCUUCACGCGGAAAGCGCCUAGCCAUGGCGUUAGUACGAGUGUCGUGCAGCCUCCAGGAC
680. KY620854.1/5-121 UUCCGAUCU--A-AU----GAGG-CGACACUCCACCAUGGAUCACUCCCCUGUGAGGAACUUCUGUCUUCACGCGGAAAGCGCCUAGCCAUGGCGUUAGUACGAGUGUCGUGCAGCCUCCAGGAC
681. KY620781.1/23-136 GGGGGCCAC----------GAGG-CGACACUCCACCAUGGAUCACUCCCCUGUGAGGAACUUCUGUCUUCACGCGGAAAGCGCCUAGCCAUGGCGUUAGUACGAGUGUCGUGCAGCCUCCAGGCC
682. KY620766.1/9-122 GGGGGCCAC----------GAGG-CGACACUCCACCAUGGAUCACUCCCCUGUGAGGAACUUCUGUCUUCACGCGGAAAGCGCCUAGCCAUGGCGUUAGUACGAGUGUCGUGCAGCCUCCAGGCC
683. KY620361.1/47-162 UUUUGUCGUU-------g-GGGG-CGACACUCCGCCAUGAAUCACUCCCCUGUGAGGAACUACUGUCUUCACGCAGAAAGCGUCUAGCCAUGGCGUUAGUAUGAGUGUCGUACAGCCUCCAGGCC
684. KY620416.1/44-159 GGGUGGUCCU---------GGAGgCGACACUCCACCAUGGAUCACUCCCCUGUGAGGAACUUCUGUCUUCACGCGGAAAGCGCCUAGCCAUGGCGUUAGUACGAGUGUCGUGCAGCCUCCAGGAC
685. KY620353.1/14-128 GCCUGGAGGCUG-UA---------CGACACUCCGCCAUGAAUCACUCCCCUGUGAGGAACUACUGUCUUCACGCAGAAAGCGUCUAGCCAUGGCGUUAGUAUGAGUGUCGUACAGCCUCCAGGCC
686. KY620340.1/33-147 GCCUGGAGGCUG-UA---------CGACACUCCGCCAUGAAUCACUCCCCUGUGAGGAACUACUGUCUUCACGCAGAAAGCGUCUAGCCAUGGCGUUAGUAUGAGUGUCGUACAGCCUCCAGGCC
687. KY620339.1/12-126 GCCUGGAGGCUG-UA---------CGACACUCCGCCAUGAAUCACUCCCCUGUGAGGAACUACUGUCUUCACGCAGAAAGCGUCUAGCCAUGGCGUUAGUAUGAGUGUCGUACAGCCUCCAGGCC
688. KY620327.1/8-122 GCCUGGAGGCUG-UA---------CGACACUCCGCCAUGAAUCACUCCCCUGUGAGGAACUACUGUCUUCACGCAGAAAGCGUCUAGCCAUGGCGUUAGUAUGAGUGUCGUACAGCCUCCAGGCC
689. KY620323.1/7-121 GCCUGGAGGCUG-UA---------CGACACUCCGCCAUGAAUCACUCCCCUGUGAGGAACUACUGUCUUCACGCAGAAAGCGUCUAGCCAUGGCGUUAGUAUGAGUGUCGUACAGCCUCCAGGCC
690. KY620317.1/4-118 GCCUGGAGGCUG-UA---------CGACACUCCGCCAUGAAUCACUCCCCUGUGAGGAACUACUGUCUUCACGCAGAAAGCGUCUAGCCAUGGCGUUAGUAUGAGUGUCGUACAGCCUCCAGGCC
691. KY620810.1/1-116 UCCGAUCUU----AC----GAGG-CGACACUCCACCAUGGAUCACUCCCCUGUGAGGAACUUCUGUCUUCACGCGGAAAGCGCCUAGCCAUGGCGUUAGUACGAGUGUCGUGCAGCCUCCAGGAC
692. KY620776.1/54-173 GGGGGGGCCCUG-GA----GGCUaCGACACUCCACCAUGGAUCACUCCCCUGUGAGGAACUUCUGUCUUCACGCGGAAAGCGCCUAGCCAUGGCGUUAGUACGAGUGUCGUGCAGCCUCCAGGAC
693. KY620343.1/39-146 GGCUGU----------------A-CGACACUCCGCCAUGAAUCACUCCCCUGUGAGGAACUACUGUCUUCACGCAGAAAGCGUCUAGCCAUGGCGUUAGUAUGAGUGUCGUACAGCCUCCAGGCC
694. KY620808.1/41-155 GGGAGUCC--UG-GA------GG-CGACACUCCACCAUAGAUCACUCCCCUGUGAGGAACUUCUGUCUUCACGCGGAAAGCGCCUAGCCAUGGCGUUAGUACGAGUGUCGUGCAGCCUCCAGGAC
695. KY620334.1/139-253 GCCUGGAGGCUG-UA---------CGACACUCCACCAUGAACCACUCCCCUGUGAGGAACUACUGUCUUCACGCAGAAAGCGUCUAGCCAUGGCGUUAGUAUGAGUGUCGUACAGCCUCCAGGCC
696. KY620733.1/3-121 UGGAGGCUGCAC-GA----CACU-CGACACUCCACCAUGGAUCACUCCCCUGUGAGGAACUUCUGUCUUCACGCGGAAAGCGCCUAGCCAUGGCGUUAGUACGAGUGUCGUGCAGCCUCCAGGAC
697. KY620483.1/1-108 -----------C-UC----GAGG-CGACACUCCACCAUGGAUCACUCCCCUGUGAGGAACUUCUGUCUUCACGCGGAAAGCGCCUAGCCAUGGCGUUAGUACGAGUGUCGUGCAGCCUCCAGGAC
698. KY620857.1/158-265 GGCUGC----------------G-CGACACUCCACCAUGGAUCACUCCCCUGUGAGGAACUUCUGUCUUCACGCGGAAAGCGCCUAGCCAUGGCGUUAGUACGAGUGUCGUGCAGCCUCCAGGAC
699. KY620850.1/15-122 GGCUGC----------------G-CGACACUCCACCAUGGAUCACUCCCCUGUGAGGAACUUCUGUCUUCACGCGGAAAGCGCCUAGCCAUGGCGUUAGUACGAGUGUCGUGCAGCCUCCAGGAC
700. KY620718.1/15-122 GGCUGC----------------G-CGACACUCCACCAUGGAUCACUCCCCUGUGAGGAACUUCUGUCUUCACGCGGAAAGCGCCUAGCCAUGGCGUUAGUACGAGUGUCGUGCAGCCUCCAGGAC
701. KY620712.1/45-152 GGCUGC----------------G-CGACACUCCACCAUGGAUCACUCCCCUGUGAGGAACUUCUGUCUUCACGCGGAAAGCGCCUAGCCAUGGCGUUAGUACGAGUGUCGUGCAGCCUCCAGGAC
702. KY620594.1/21-128 GGCUGC----------------G-CGACACUCCACCAUGGAUCACUCCCCUGUGAGGAACUUCUGUCUUCACGCGGAAAGCGCCUAGCCAUGGCGUUAGUACGAGUGUCGUGCAGCCUCCAGGAC
703. KY620524.1/14-121 GGCUGC----------------G-CGACACUCCACCAUGGAUCACUCCCCUGUGAGGAACUUCUGUCUUCACGCGGAAAGCGCCUAGCCAUGGCGUUAGUACGAGUGUCGUGCAGCCUCCAGGAC
704. KY620434.1/15-122 GGCUGC----------------G-CGACACUCCACCAUGGAUCACUCCCCUGUGAGGAACUUCUGUCUUCACGCGGAAAGCGCCUAGCCAUGGCGUUAGUACGAGUGUCGUGCAGCCUCCAGGAC
705. KY620412.1/44-151 GGCUGC----------------G-CGACACUCCACCAUGGAUCACUCCCCUGUGAGGAACUUCUGUCUUCACGCGGAAAGCGCCUAGCCAUGGCGUUAGUACGAGUGUCGUGCAGCCUCCAGGAC
706. KY620390.1/16-123 GGCUGC----------------G-CGACACUCCACCAUGGAUCACUCCCCUGUGAGGAACUUCUGUCUUCACGCGGAAAGCGCCUAGCCAUGGCGUUAGUACGAGUGUCGUGCAGCCUCCAGGAC
707. KY620454.1/7-122 GGGGGGUCCU---------GGAGgCGACACUCCACCAUGGAUCACUCCCCUGUGAGGAACUUCUGUCUUCACGCGGAAAGCGCCUAGCCAUGGCGUUAGUACGAGUGUCGUGCAGCCUCCAGGAC
708. KY620624.1/4-119 GGGGGGUCCU---------GGAGgCGACACUCCACCAUGGAUCACUCCCCUGUGAGGAACUUCUGUCUUCACGCGGAAAGCGCCUAGCCAUGGCGUUAGUACGAGUGUCGUGCAGCCUCCAGGAC
709. KY620614.1/5-120 GGGGGGUCCU---------GGAGgCGACACUCCACCAUGGAUCACUCCCCUGUGAGGAACUUCUGUCUUCACGCGGAAAGCGCCUAGCCAUGGCGUUAGUACGAGUGUCGUGCAGCCUCCAGGAC
710. KY620590.1/2-117 GGGGGGUCCU---------GGAGgCGACACUCCACCAUGGAUCACUCCCCUGUGAGGAACUUCUGUCUUCACGCGGAAAGCGCCUAGCCAUGGCGUUAGUACGAGUGUCGUGCAGCCUCCAGGAC
711. KY620405.1/7-122 GGGGGGUCCU---------GGAGgCGACACUCCACCAUGGAUCACUCCCCUGUGAGGAACUUCUGUCUUCACGCGGAAAGCGCCUAGCCAUGGCGUUAGUACGAGUGUCGUGCAGCCUCCAGGAC
712. KY620813.1/9-126 ACAACACUCUUA--C----GAGG-CGACACUCCACCAUGGAUCACUCCCCUGUGAGGAACUUCUGUCUUCACGCGGAAAGCGCCUAGCCAUGGCGUUAGUACGAGUGUUGUGCAGCCUCCAGGCC
713. KY620315.1/7-114 GGCUGU----------------G-CGACACUCCGCCAUGAAUCACUCCCCUGUGAGGAACUACUGUCUUCACGCAGAAAGCGUCUAGCCAUGGCGUUAGUAUGAGUGUCGUACAGCCUCCAGGCC
714. KY620735.1/1-117 GGGGGU--CCUU-AC----GAGG-CGACACUCCACCACGGAUCACUCCCCUGUGAGGAACUUCUGUCUUCACGCGGAAAGCGCCUAGCCAUGGCGUUAGUACGAGUGUCGUGCAGCCUCCAGGAC
715. KY620506.1/1-116 --CGAUCUCUUA--C----GAGG-CGACACUCCACCAUGGAUCACUCCCCUGUGAGGAACUUCUGUCUUCACGCGGAAAGCGCCUAGCCAUGGCGUUAGUACGAGUGUCGUGCAGCCUCCAGGAC
716. KY620578.1/1-116 UCCGAUCUU----AC----GAGG-CGACACUCCACCAUGGAUCACUCCCCUGUGAGGAACUUCUGUCUUCACGCGGAAAGCGCCUAGCCAUGGCGUUAGUACGAGUGUCGUGCAGCCUCCAGGCC
717. KY620744.1/173-291 GGGGUCCUGGAG-GC----GAGG-CGACACUCCACCAUGAAUCACUCCCCUGUGAGGAACUUCUGUCUUCACGCGGAAAGCGUCUAGCCAUGGCGUUAGUACGAGUGUCGUGCAGCCUCCAGGAC
718. KY620616.1/2-117 UUCCGAUCUA---CG----A-GG-CGACACUCCACCAUGGAUCACUCCCCUGUGAGGAACUUCUGUCUUCACGCGGAAAGCGCCUAGCCAUGGCGUUAGUACGAGUGUCGUGCAGCCUCCAGGAC
719. KY620410.1/10-128 GGGGUCCUGGAG-GC----GAGG-CGACACUCCACCAUGGAUCACUCCCCUGUGAGGAACUUCUGUCUUCACGCGGAAAGCGUCUAGCCAUGGCGUUAGUACGAGUGUCGUGCAGCCUCCAGGAC
720. KY620365.1/36-147 ACAUGGCU------------AGG-CGACACUCCACCAUGGAUCACUCCCCUGUGAGGAACUUCUGUCUUCACGCGGAAAGCGCCUAGCCAUGGCGUUAGUACGAGUGUCGUGCAGCCUCCAGGCC
721. KY620324.1/139-253 GCCUGGAGGCUG-UA---------CGACACUCCGCCAUGAACCACUCCCCUGUGAGGAACUACUGUCUUCACGCAGAAAGCGUCUAGCCAUGGCGUUAGUAUGAGUGUCGUACAGCCUCCAGGCC
722. KY620605.1/5-112 GGCUGC----------------G-CGACACUCCACCAUGGAUCACUCCCCUGUGAGGAACUUCUGUCUUCACGCGGAAAGCGCCUAGCCAUGGCGUUAGUACGAGUGUCGUGCAGCCUCCAGGCC
723. KY620420.1/35-142 GGCUGC----------------G-CGACACUCCACCAUGGAUCACUCCCCUGUGAGGAACUUCUGUCUUCACGCGGAAAGCGCCUAGCCAUGGCGUUAGUACGAGUGUCGUGCAGCCUCCAGGCC
724. KY620396.1/14-121 GGCUGC----------------G-CGACACUCCACCAUGGAUCACUCCCCUGUGAGGAACUUCUGUCUUCACGCGGAAAGCGCCUAGCCAUGGCGUUAGUACGAGUGUCGUGCAGCCUCCAGGCC
725. KY620352.1/102-216 GGGGGUC--CA------c-GAGG-CGACACUCCACCAUGAAUCACUCCCCUGUGAGGAACUUCUGUCUUCACGCAGAAAGCGUCUAGCCAUGGCGUUAGUAUGAGUGUCGUACAGCCUCCAGGCC
726. KY620635.1/122-241 GGGGGAGUCCUG-GA----GGCUaCGACACUCCACCAUGGAUCACUCCCCUGUGAGGAACUUCUGUCUUCACGCGGAAAGCGCCUAGCCAUGGCGUUAGUACGAGUGUCGUGCAGCCUCCAGGAC
727. KY620541.1/125-244 GGGGGAGUCCUG-GA----GGCUaCGACACUCCACCAUGGAUCACUCCCCUGUGAGGAACUUCUGUCUUCACGCGGAAAGCGCCUAGCCAUGGCGUUAGUACGAGUGUCGUGCAGCCUCCAGGAC
728. KY620814.1/7-122 GGGGGGUCCU---------GGAGgCAACACUCCACCAUGGAUCACUCCCCUGUGAGGAACUUCUGUCUUCACGCGGAAAGCGCCUAGCCAUGGCGUUAGUACGAGUGUCGUGCAGCCUCCAGGAC
729. KY620744.1/10-124 UCCUGGAGGCUG-CA---------CGACACUCCACCAUGAAUCACUCCCCUGUGAGGAACUUCUGUCUUCACGCGGAAAGCGUCUAGCCAUGGCGUUAGUACGAGUGUCGUGCAGCCUCCAGGAC
730. KY620794.1/3-117 AUCUGGAGGCUG-CA---------CGACACUCCACCAUGGAUCACUCCCCUGUGAGGAACUUCUGUCUUCACGCGGAAAGCGCCUAGCCAUGGCGUUAGUACGAGUGUCGUGCAGCCUCCAGGAC
731. KY620752.1/5-119 AUCUGGAGGCUG-CA---------CGACACUCCACCAUGGAUCACUCCCCUGUGAGGAACUUCUGUCUUCACGCGGAAAGCGCCUAGCCAUGGCGUUAGUACGAGUGUCGUGCAGCCUCCAGGAC
732. JF343793.1/51-162 GGACGUGA------------UCA-UGACACUCCGCCAUGAAUCACUCCCCUGUGAGGAACUACUGUCUUCACGCAGAAAGCGUCUAGCCAUGGCGUUAGUAUGAGUGUCGUACAGCCUCCAGGCC
733. KY620771.1/3-117 UCCUGGAGGCUG-CA---------CGACACUCCACCAUAGAUCACUCCCCUGUGAGGAACUUCUGUCUUCACGCGGAAAGCGUCUAGCCAUGGCGUUAGUACGAGUGUCGUGCAGCCUCCAGGAC
734. KY620830.1/70-184 UCCUGGAGGCUG-CA---------CGACACUCCACCAUGGAUCACUCCCCUGUGAGGAACUUCUGUCUUCACGCGGAAAGCGUCUAGCCAUGGCGUUAGUACGAGUGUCGUGCAGCCUCCAGGAC
735. KY620333.1/32-150 GGCCCUGCGCGG-UA-----GCAaGUAUACUCCGCCAUGAAUCACUCCCCUGUGAGGAACUACUGUCUUCACGCAGAAAGCGUCUAGCCAUGGCGUUAGUAUGAGUGUCGUACAGCCUCCAGGCC
736. KY620591.1/9-123 UCCUGGAGGCUG-CA---------CGACACUCCACCAUAGAUCACUCCCCUGUGAGGAACUUCUGUCUUCACGCGGAAAGCGCCUAGCCAUGGCGUUAGUACGAGUGUCGUGCAGCCUCCAGGAC
737. KY620502.1/24-138 GGCUAGACG--C-UU------UC-CGACACUCCACCAUGGAUCACUCCCCUGUGAGGAACUUCUGUCUUCACGCGGAAAGCGCCUAGCCAUGGCGUUAGUACGAGUGUCGUGCAGCCUCCAGGAC
738. KY620817.1/4-118 UCCUGGAGGCUG-CA---------CGACACUCCACCAUGGAUCACUCCCCUGUGAGGAACUUCUGUCUUCACGCGGAAAGCGCCUAGCCAUGGCGUUAGUACGAGUGUCGUGCAGCCUCCAGGAC
739. KY620762.1/17-131 UCCUGGAGGCUG-CA---------CGACACUCCACCAUGGAUCACUCCCCUGUGAGGAACUUCUGUCUUCACGCGGAAAGCGCCUAGCCAUGGCGUUAGUACGAGUGUCGUGCAGCCUCCAGGAC
740. KY620413.1/22-136 UCCUGGAGGCUG-CA---------CGACACUCCACCAUGGAUCACUCCCCUGUGAGGAACUUCUGUCUUCACGCGGAAAGCGCCUAGCCAUGGCGUUAGUACGAGUGUCGUGCAGCCUCCAGGAC
741. KY620404.1/20-134 UCCUGGAGGCUG-CA---------CGACACUCCACCAUGGAUCACUCCCCUGUGAGGAACUUCUGUCUUCACGCGGAAAGCGCCUAGCCAUGGCGUUAGUACGAGUGUCGUGCAGCCUCCAGGAC
742. KY620364.1/19-133 UCCUGGAGGCUG-CA---------CGACACUCCACCAUGGAUCACUCCCCUGUGAGGAACUUCUGUCUUCACGCGGAAAGCGCCUAGCCAUGGCGUUAGUACGAGUGUCGUGCAGCCUCCAGGAC
743. KY620861.1/159-273 UCCUGGAGGCUG-CA---------CGACACUCCACCAUGGAUCACUCCCCUGUGAGGAACUUCUGUCUUCACGCGGAAAGCGCCUAGCCAUGGCGUUAGUACGAGUGUCGUGCAGCCUCCAGGAC
744. KY620823.1/72-186 UCCUGGAGGCUG-CA---------CGACACUCCACCAUGGAUCACUCCCCUGUGAGGAACUUCUGUCUUCACGCGGAAAGCGCCUAGCCAUGGCGUUAGUACGAGUGUCGUGCAGCCUCCAGGAC
745. KY620819.1/7-121 UCCUGGAGGCUG-CA---------CGACACUCCACCAUGGAUCACUCCCCUGUGAGGAACUUCUGUCUUCACGCGGAAAGCGCCUAGCCAUGGCGUUAGUACGAGUGUCGUGCAGCCUCCAGGAC
746. KY620812.1/8-122 UCCUGGAGGCUG-CA---------CGACACUCCACCAUGGAUCACUCCCCUGUGAGGAACUUCUGUCUUCACGCGGAAAGCGCCUAGCCAUGGCGUUAGUACGAGUGUCGUGCAGCCUCCAGGAC
747. KY620807.1/29-143 UCCUGGAGGCUG-CA---------CGACACUCCACCAUGGAUCACUCCCCUGUGAGGAACUUCUGUCUUCACGCGGAAAGCGCCUAGCCAUGGCGUUAGUACGAGUGUCGUGCAGCCUCCAGGAC
748. KY620804.1/232-346 UCCUGGAGGCUG-CA---------CGACACUCCACCAUGGAUCACUCCCCUGUGAGGAACUUCUGUCUUCACGCGGAAAGCGCCUAGCCAUGGCGUUAGUACGAGUGUCGUGCAGCCUCCAGGAC
749. KY620777.1/7-121 UCCUGGAGGCUG-CA---------CGACACUCCACCAUGGAUCACUCCCCUGUGAGGAACUUCUGUCUUCACGCGGAAAGCGCCUAGCCAUGGCGUUAGUACGAGUGUCGUGCAGCCUCCAGGAC
750. KY620772.1/50-164 UCCUGGAGGCUG-CA---------CGACACUCCACCAUGGAUCACUCCCCUGUGAGGAACUUCUGUCUUCACGCGGAAAGCGCCUAGCCAUGGCGUUAGUACGAGUGUCGUGCAGCCUCCAGGAC
