# Supplementary material for: microRNA-122 amplifies hepatitis C virus translation by shaping the structure of the internal ribosomal entry site
Source: Nat Commun. 2018 Jul 4;9:2613. doi: 10.1038/s41467-018-05053-3 (PMC6031695; doi:10.1038/s41467-018-05053-3)
Supplement: Supplementary file 2 — Supplementary Information [file 41467_2018_5053_MOESM2_ESM.pdf]

Supplementary Information

Supplementary Figures

microRNA-122 amplifies hepatitis C virus translation by shaping the structure of the internal ribosomal entry site

Schult et al.

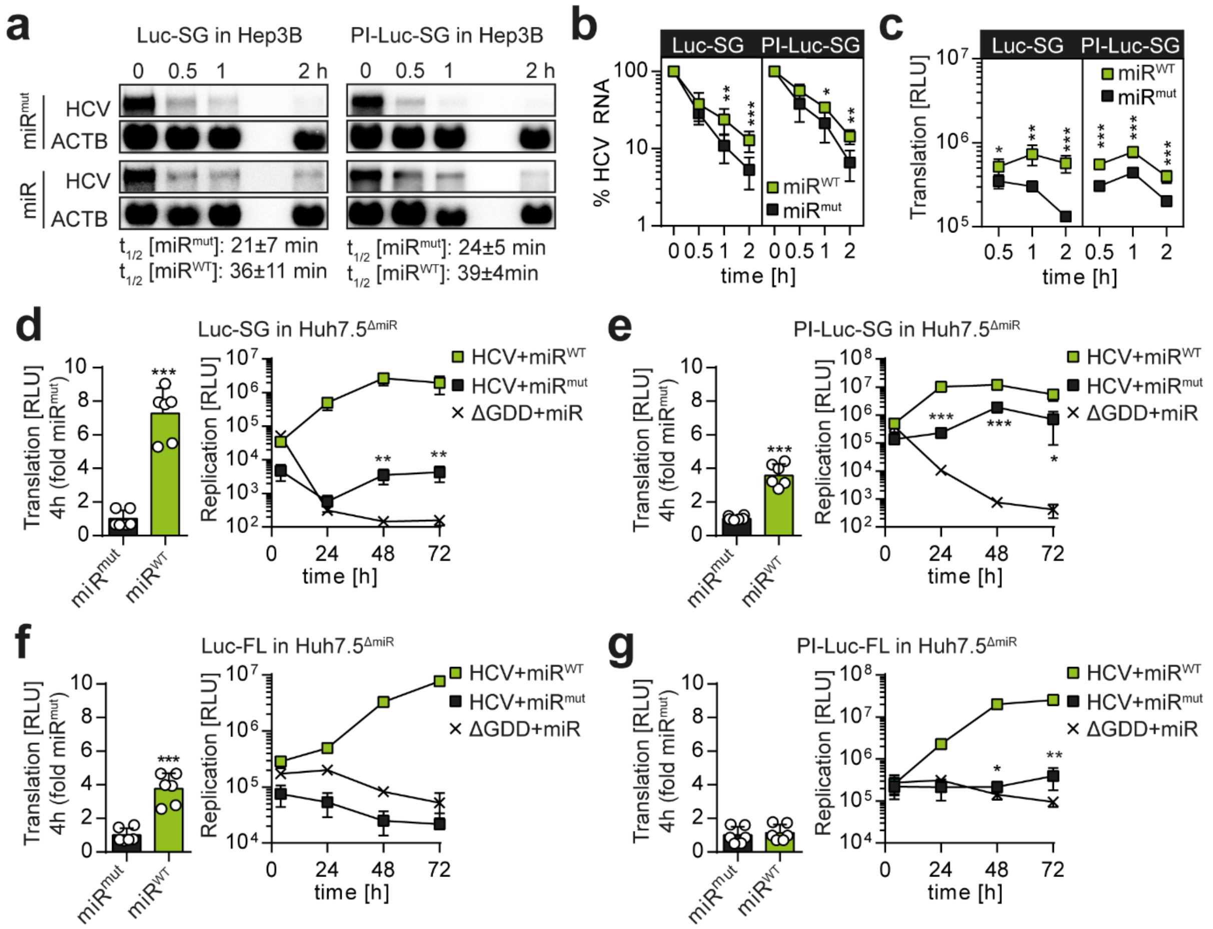

Supplementary Figure 1 – Influence of RNA stability and different cellular backgrounds. (a) Representative autoradiography images of short-term northern blot analysis of reporter replicons used in Fig 1. **(b)** Quantification of RNA stability based on northern blots. All values are normalized to 0 h from the respective experiment. Half-life times are given below for each condition. **(c)** Luciferase assay at the time points corresponding to the northern blots as a measure of translation efficiency. **(d-g)** Validation of experiments shown in Fig 1e-h using Huh7.5$^{\Delta miR}$ instead of Hep3B cells. Mean values (±SD), n=3, in technical duplicates. *RLU*: relative light units; *ΔGDD*: replication deficient mutant. For translation, statistical significance was determined for miR^{WT} against miR^{mut}, for replication miR^{mut} was tested against ΔGDD. *: P<=0.05, **: P<=0.01, ***: P<0.001.

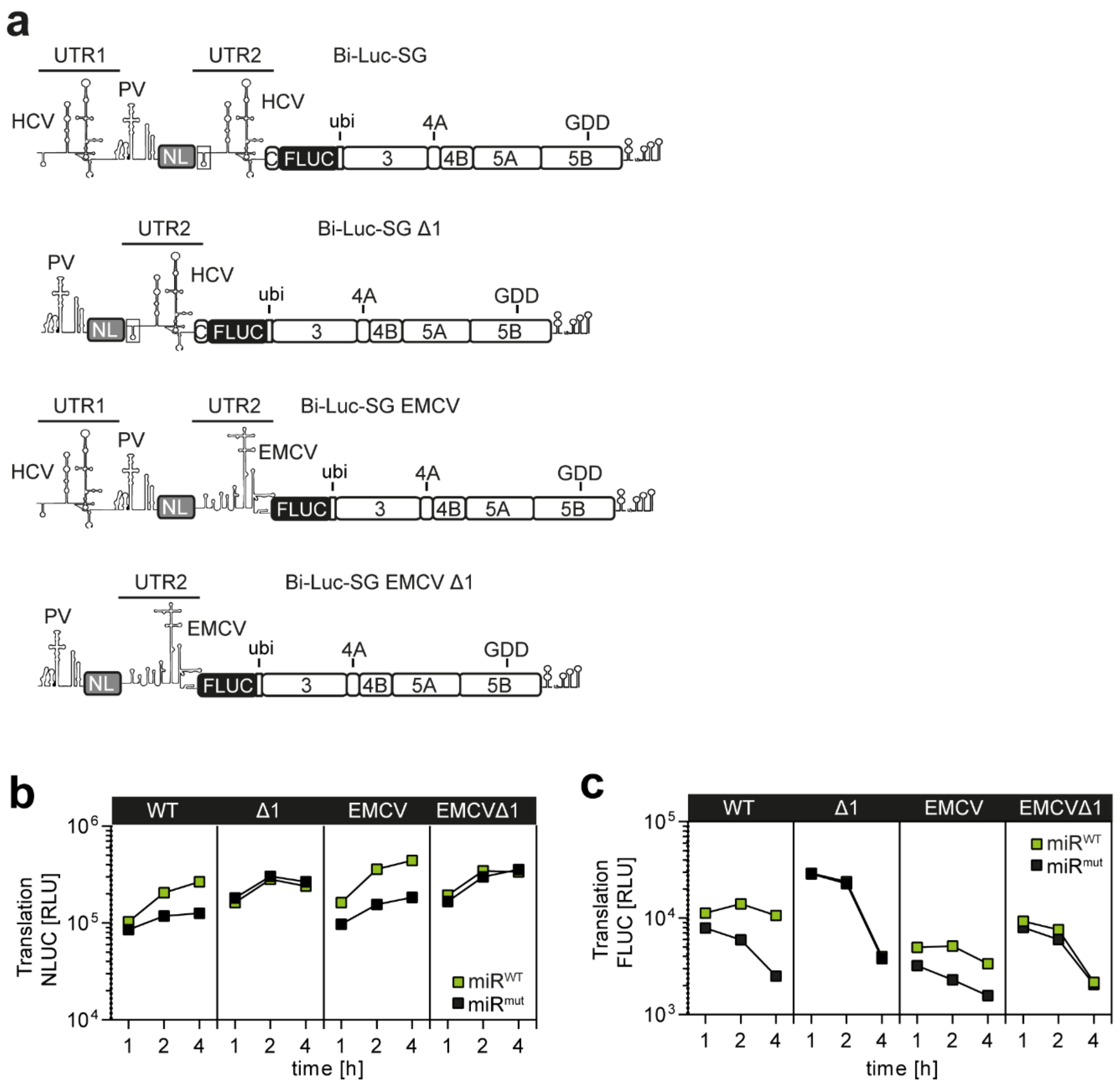

Supplementary Figure 2 - Assessment of potential crosstalk of IRESs in BI-Luc-SG (a) Illustration BI-Luc-SG variants used to study effects of several IRES elements in cis. The previously used Bi-Luc-SG was modified by removing the first HCV UTR to generate Bi-Luc-SG Δ1. Also, another Bicistronic replicon was created replacing UTR2 by the EMCV IRES (Bi-Luc-SG EMCV). This construct was also modified by removing the 5' HCV UTR1 to generate Bi-Luc-SG EMCV Δ1. **(b, c)** Nano (NLUC) and Firefly luciferase (FLUC) signal from the BI-Luc-SG variants, respectively, after transfection of the indicated constructs in Hep3B cells, in presence of miR^{WT} or miR^{mut}. Note that in (c) the data points of Δ1 with miR^{mut} obscure the miR^{WT} data. The lack of stimulation of FLUC activity for this construct by miR^{wt} indicates a poor responsiveness of UTR2 to miR-122 in this configuration. All variants were used in the replication deficient ΔGDD context. Mean values (±SD), n=2, in technical duplicates. *RLU*: relative light units.

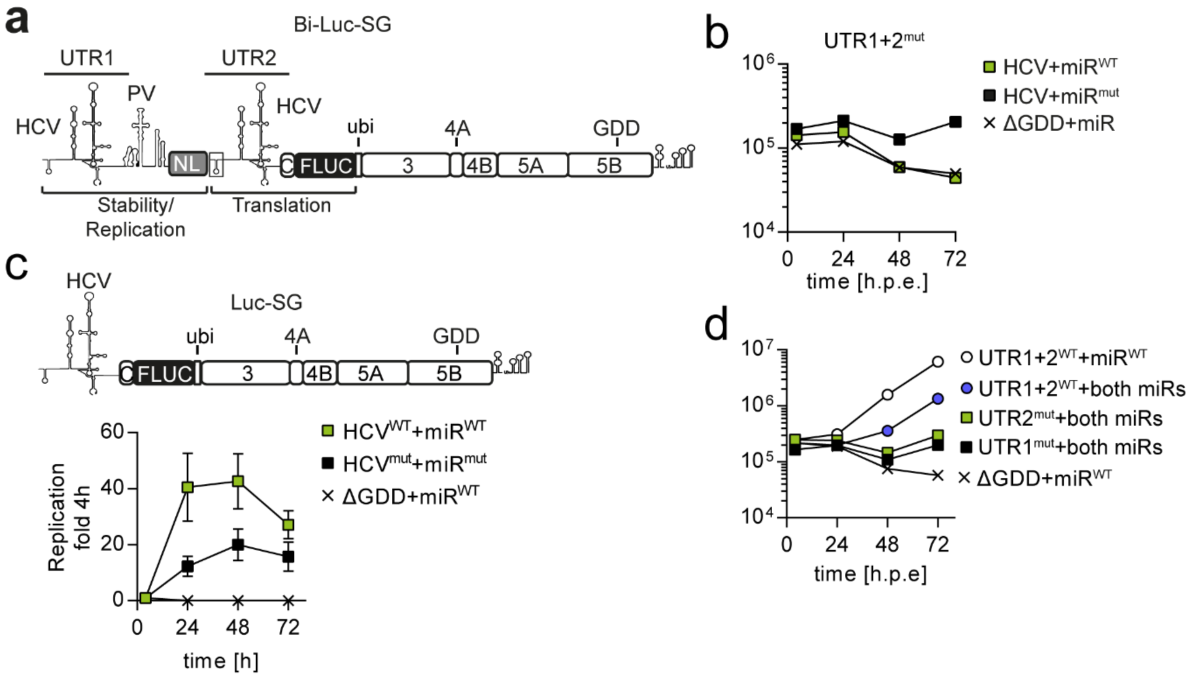

Supplementary Figure 3 – Incomplete rescue of replicons by miR^{mut}. (a) Illustration of the bicistronic replicon used for the rescue experiment. **(b)** Luciferase assay of a BI-Luc-SG replicon with both HCV UTRs mutated, either supplemented with miR^{WT} or miR^{mut}. The Nano luciferase signal was used to monitor translation. n=2, in technical duplicates. Note that replication could be rescued by miRmut only, but with a strongly reduced efficiency compared to UTR1+2^{WT} (Fig. 2e) **(c)** Illustration of the used Luc-SG replicon and comparison of the stimulation of wild type Luc-SG (HCV^{WT}) by miR^{WT} and a mutated version HCV^{mut} by miR^{mut}. Data were normalized to the respective 4h values. Note that the y-axis is represented in linear scale here to clarify the moderate differences. n=2, in technical duplicates. Note that rescue of HCV^{mut} by miR^{mut} is less efficient than HCV^{WT} by miR^{WT}. **(d)** Luciferase assay of BI-Luc-SG variants, supplementing both, miR^{WT} and miR^{mut} simultaneously. Wild type BI-Luc-SG and either one or both miRs was used as control. n=1, in technical duplicate. ΔGDD was used as negative control in all experiments. Mean values (±SD), in technical duplicates. *RLU*: relative light units; *ΔGDD*: replication deficient mutant.

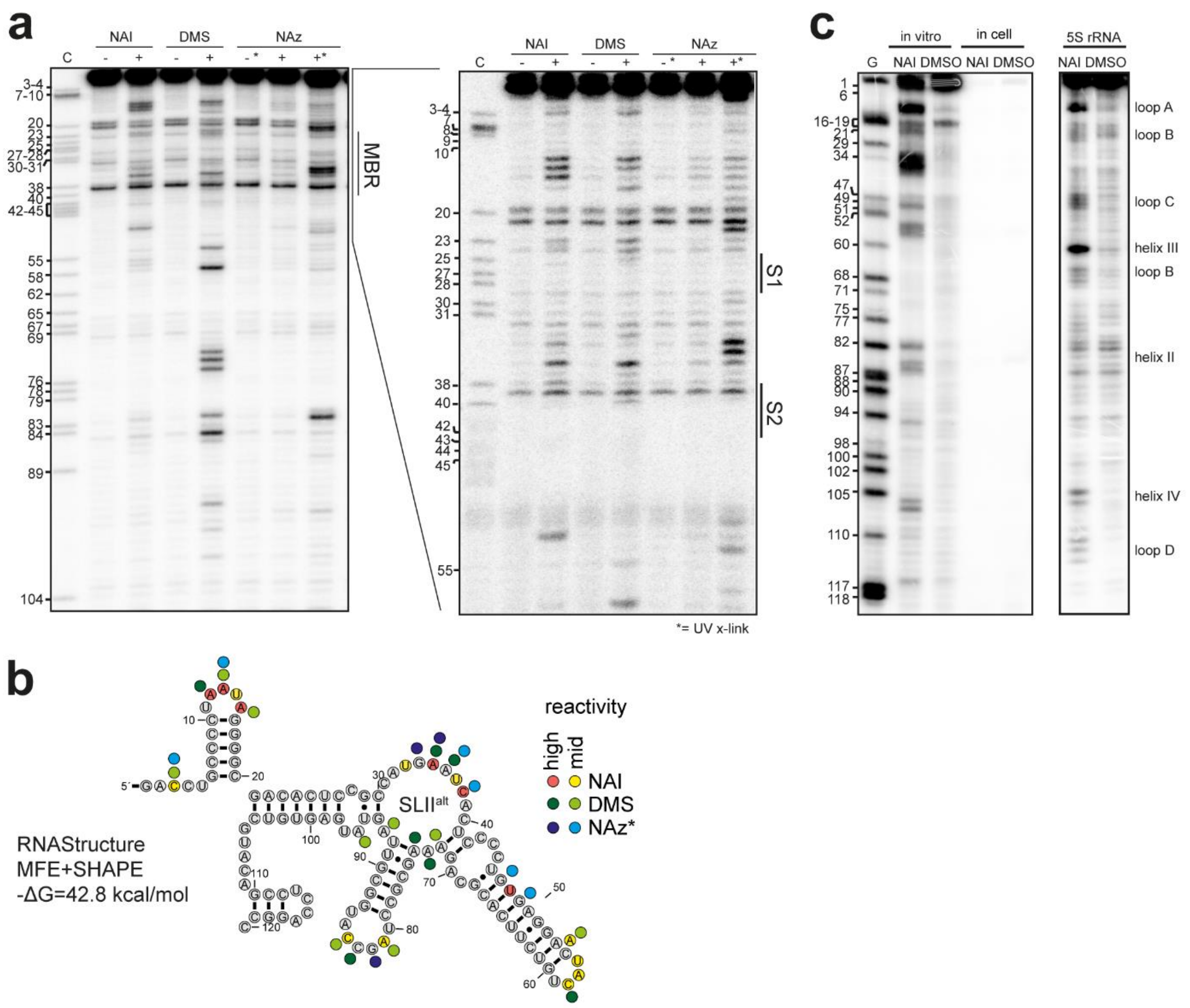

Supplementary Figure 4 - Further biochemical characterization of the HCV 5' UTR structure in the full length HCV genome. (a) Biochemical probing of HCV RNA structure using different labeling reagents. The autoradiography images show experiments with primers for the full 120nt (left), and a more detailed view of the MBR. The asterisk indicates UV crosslinking **(b)** Assignment of the measured reactivities of all used reagents to the hypothesized structure of the HCV 5' UTR. DMS exclusively modifies A and C, while NAz recognizes purines. In both cases only reactivities mapping to the respective types of nucleotides were used for analysis. **(c)** Trial of intracellular labeling of HCV RNA with NAI, 30min after transfection. In the autoradiography image, the *in vitro* data is shown as comparison. As a technical control, the same labeled total RNA was used to perform structural analysis of 5S rRNA.

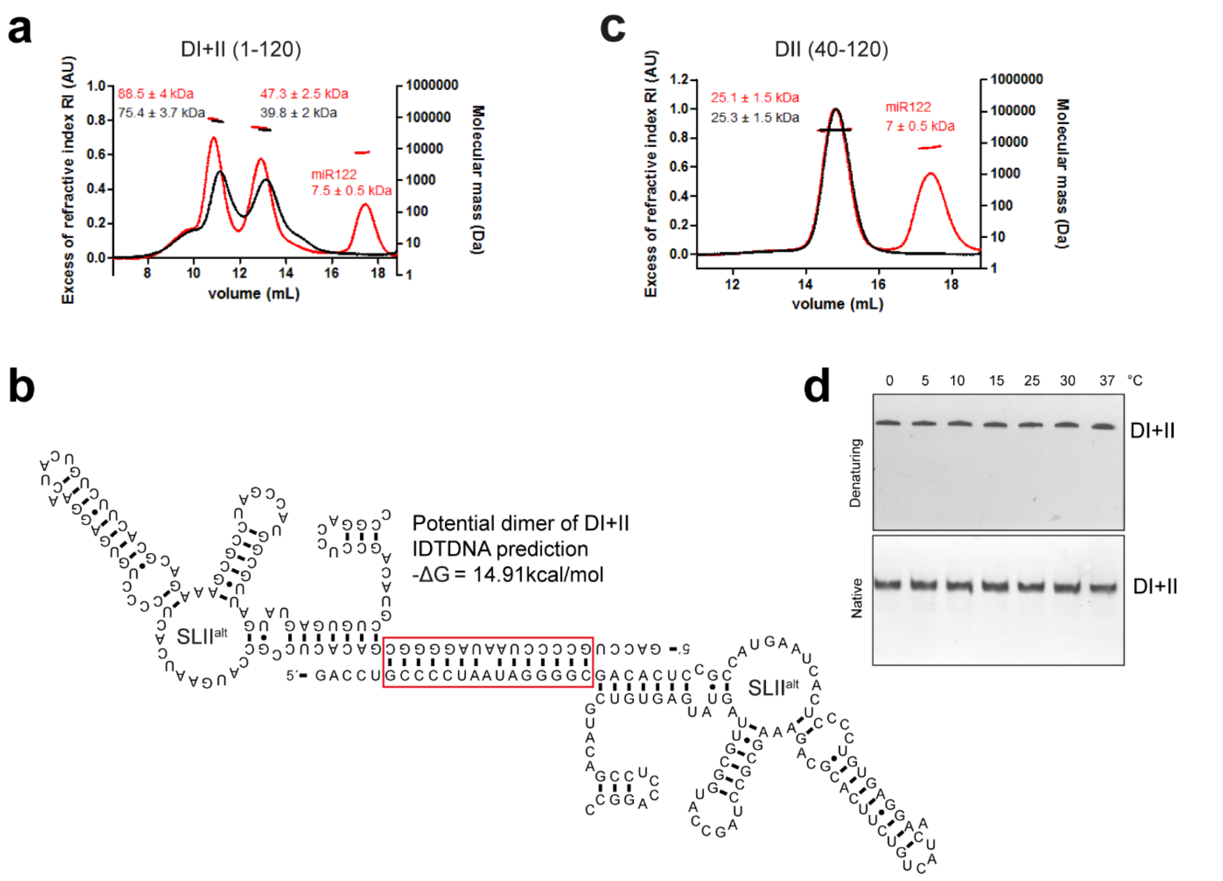

Supplementary Figure 5 - Molecular mass and stoichiometry determination by SEC-MALLS-RI analysis of different 5'UTR constructs and miR-122. (a) SEC-MALLS-RI analysis of the DI+II fragment in absence (black) or presence (red) of miR-122. The chromatogram shows the elution profile monitored by excess refractive index (left ordinate axis). The lines above the elution peaks show the molecular mass (right ordinate axis) derived from MALLS and refractometry measurements. The theoretical masses calculated for calculated for the DI+II monomer is 36.51 KDa, and 73.02 kDa for a dimer. The theoretical mass of the free miR-122 is 7.05 kDa. **(b)** Depiction of a potential dimer of DI+II and calculation of binding energy. The red box indicates the predicted dimerization site. **(c)** Experiment as in (a) for the DII fragment. The theoretical mass is 24.34 kDa. **(d)** Native gel analysis of DI+II at different temperatures to determine the presence of oligomers and to assess potential separation of conformers. A denaturing control was performed to confirm integrity of the used RNA.

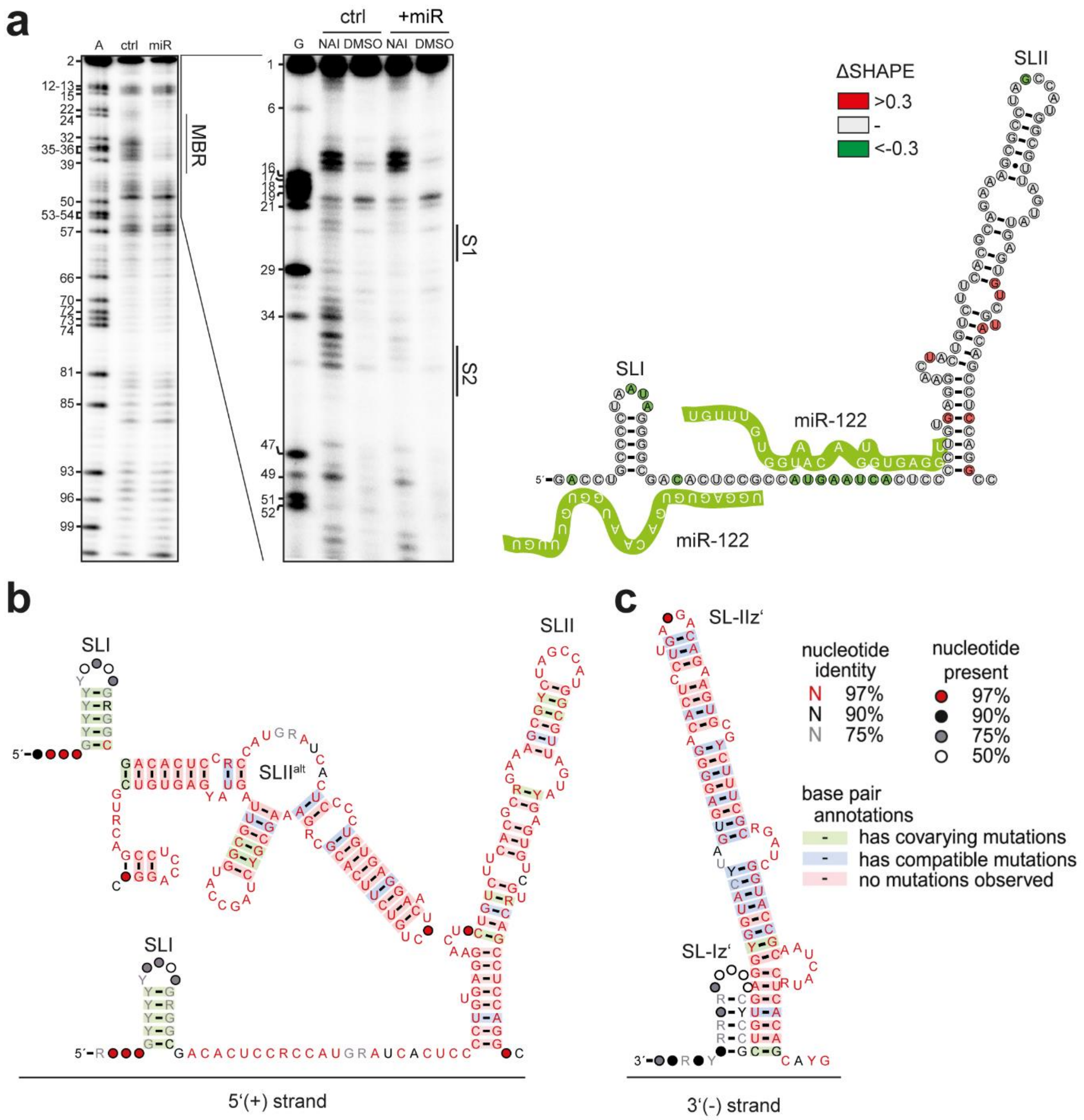

Supplementary Figure 6 – Biochemical and *in silico* investigation of *in vitro* miR-122 binding and phylogeny analysis. (a) Autoradiography image of *in vitro* SHAPE in absence or presence of single stranded miR-122-5p of the full 120 nt and a detailed view of the MBR (left) and bimolecular folding model with two molecules of miR-122 bound to the MBR visualized with R2R (right). Note that the enlarged region of the autoradiograph is taken from an independent experiment. The normalized reactivities of and miR-supplemented HCV RNAs were subtracted from those of the native RNA. All differences above 0.3 were considered significant and color coded in the structure as indicated. **(b)** Phylogeny analysis of SLII^{alt} and SLII among HCV genotypes. 750 sequences of HCV were aligned, and their structural conservation was tested using the Infernal software and visualized with R2R. **(c)** Conservation of the 3' negative strand structure (SL-IIz'), using the complementary sequences. *Covarying mutations*: Exchanges of base pairing un both sides, e.g. C:G to G:C. *Compatible mutations*: e.g. A:U to G:U. *Nucleotide identity*: Conservation of the nucleotide among genotypes. *Nucleotide present*: Measurement of gaps in the alignment.

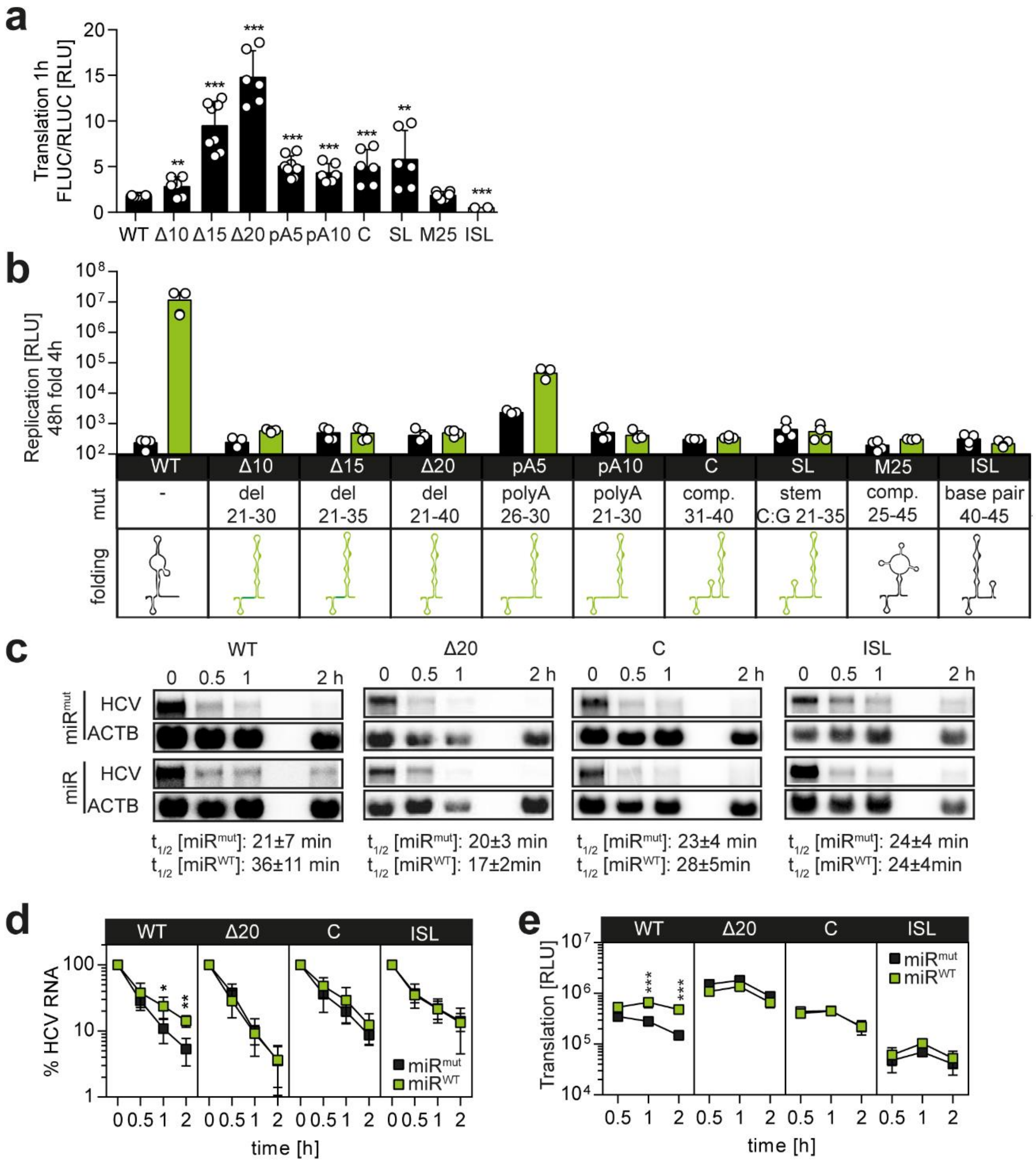

Supplementary Figure 7 – Characterization of DI mutants in the Luc-SG replicon. (a) Bar graph representation of the 1h values from Fig 4c, comparing the initial translation of the DI mutants to wild type in presence of miR^{mut}. **(b)** Replication of Luc-SG replicon harboring the DI mutants 48h after electroporation, compared to wild-type in presence of miR^{mut} or miR^{WT}. Mean values (±SD) n=2, in technical duplicates. **(c)** Representative autoradiography images of short-term northern blot analysis to determine RNA decay kinetics of selected replicons used in Fig 3b. **(d)** Quantification of northern blots from (c). All values were normalized to the 0 h value from the respective experiment. **(e)** Luciferase production at the time points assessed by northern blotting. Mean values (±SD), n=3, in technical duplicates. *RLU*: relative light units; *ΔGDD*: replication deficient mutant. *: P<=0.05, **: P<=0.01, ***: P<0.001. In the translation assay, significance was determined for each mutant against wild type with miR^{mut}. For northern blots and translation experiments, statistical significance was determined for miR^{WT} against miR^{mut}.

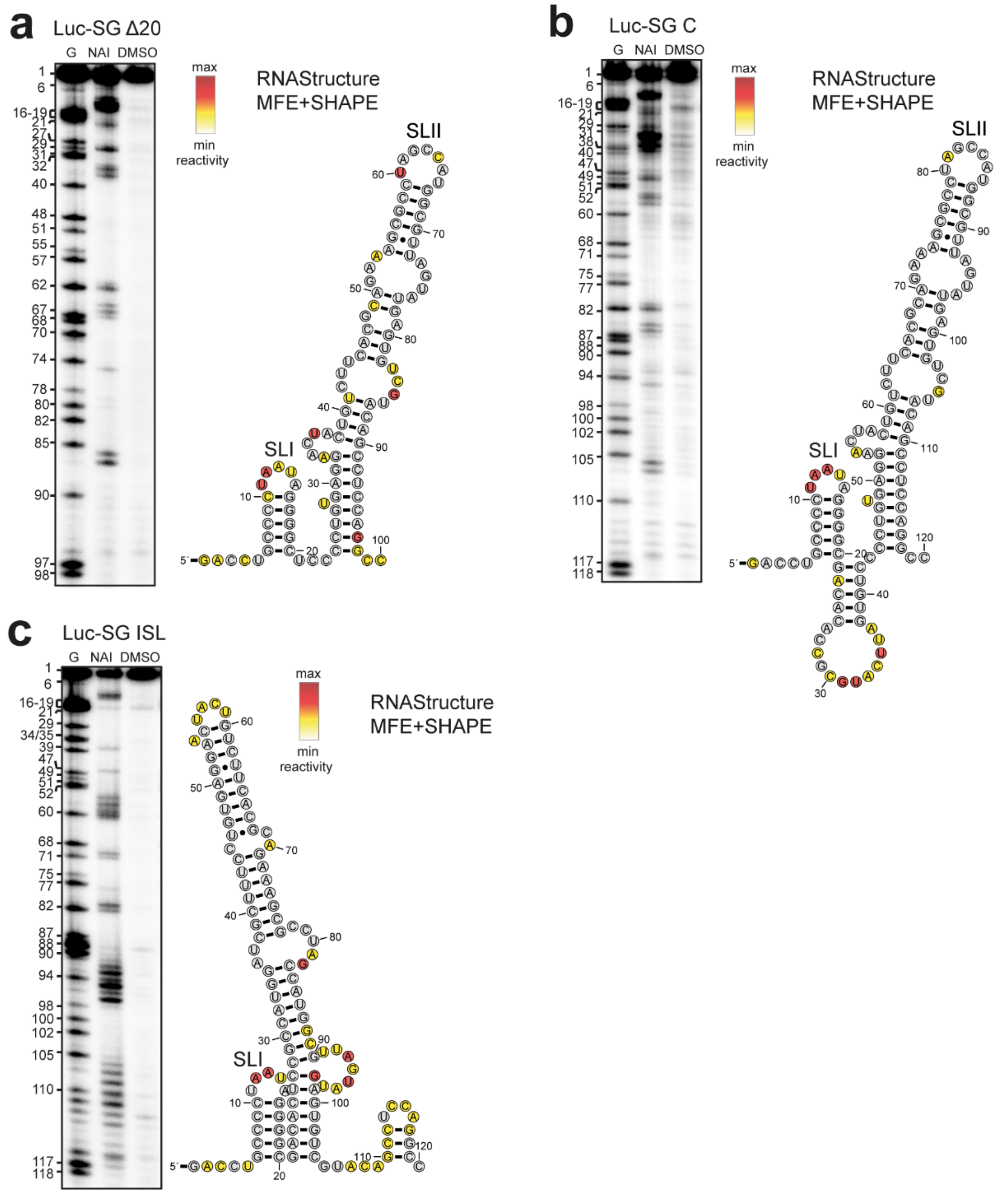

Supplementary Figure 8 – Structural assessment of selected DI mutants. SHAPE assays of selected mutants. Representative autoradiography images showing the *in vitro* SHAPE reactivity of nucleotides 1-120 of a JFH reporter replicon with the **(a)** Δ20, **(b)** C, or **(c)** ISL mutations in DI. The SHAPE data were used to predict the corresponding secondary structure of DI and II with the RNAstructure web tool and visualized with R2R. The nucleotides are colored according to their respective normalized reactivity.

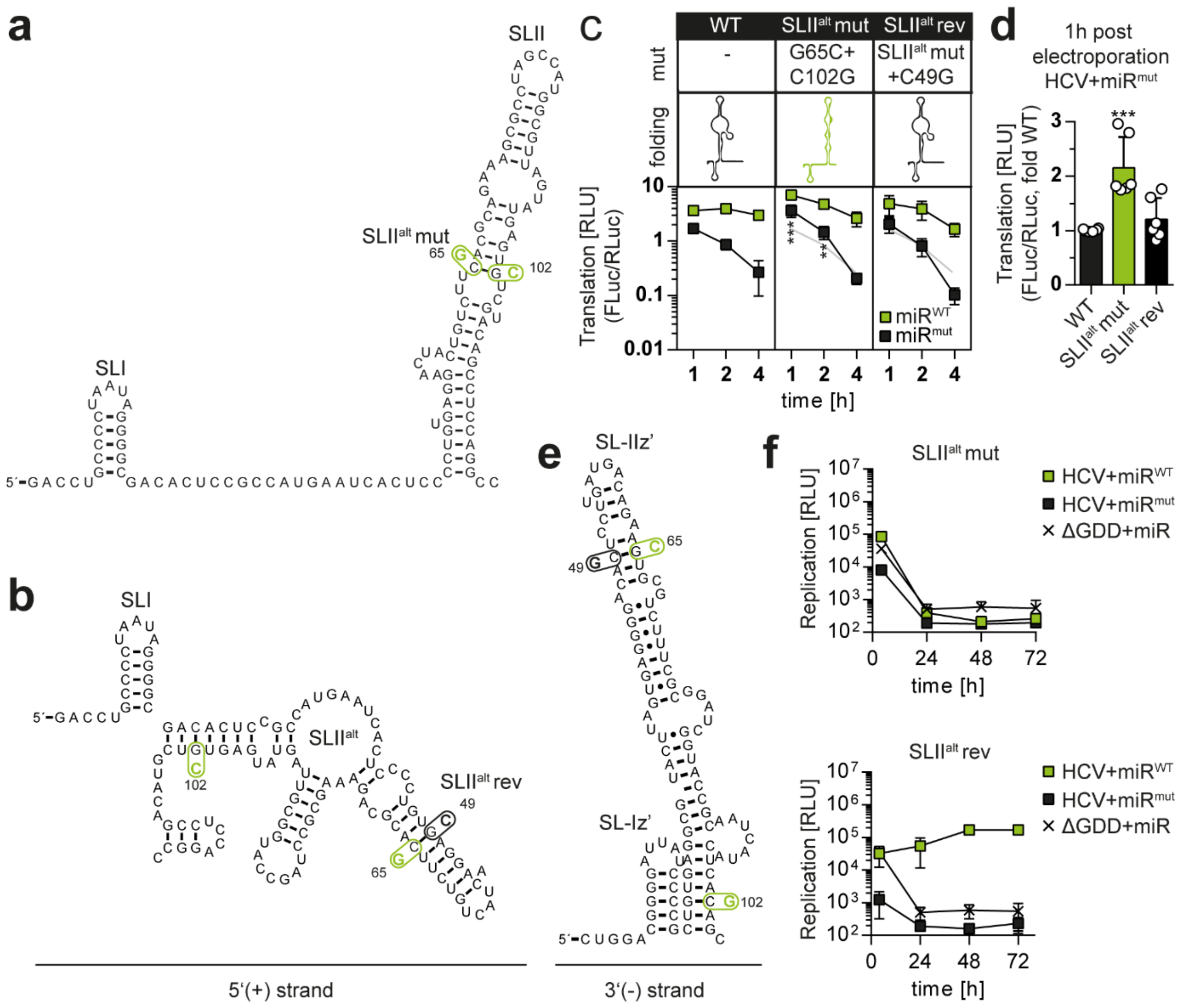

Supplementary Figure 9 – Additional mutations outside the MBR affecting SLII^{alt} **(a)** Introduced mutations and predicted MFE structure of SLII^{alt} mut. SLII is maintained by the complementary exchange, while SLII^{alt} is destabilized. **(b)** Structure of the reversion mutant, SLII^{alt} rev, forming SLII^{alt} as MFE structure. **(c)** Translation assay using SLII^{alt} mut and SLII^{alt} rev in presence of miR^{WT} or miR^{mut}. **(d)** Enlarged view of the 1h timepoint with miR^{mut}. **(e)** SLIIz' is also destabilized by mutations G65C/C102G and restored by C49G. **(f)** Replication of SLII^{alt} mut and SLII^{alt} rev in presence of miR^{WT} or miR^{mut}. Mean values (±SD), n=3, in technical duplicates. *ΔGDD*: replication deficient mutant. For translation experiments, statistical significance was determined for WT against the mutant in presence of miR^{mut}. The reference graph used to calculate is given in light grey for each subpanel. *: P<=0.05, **: P<=0.01, ***: P<0.001.

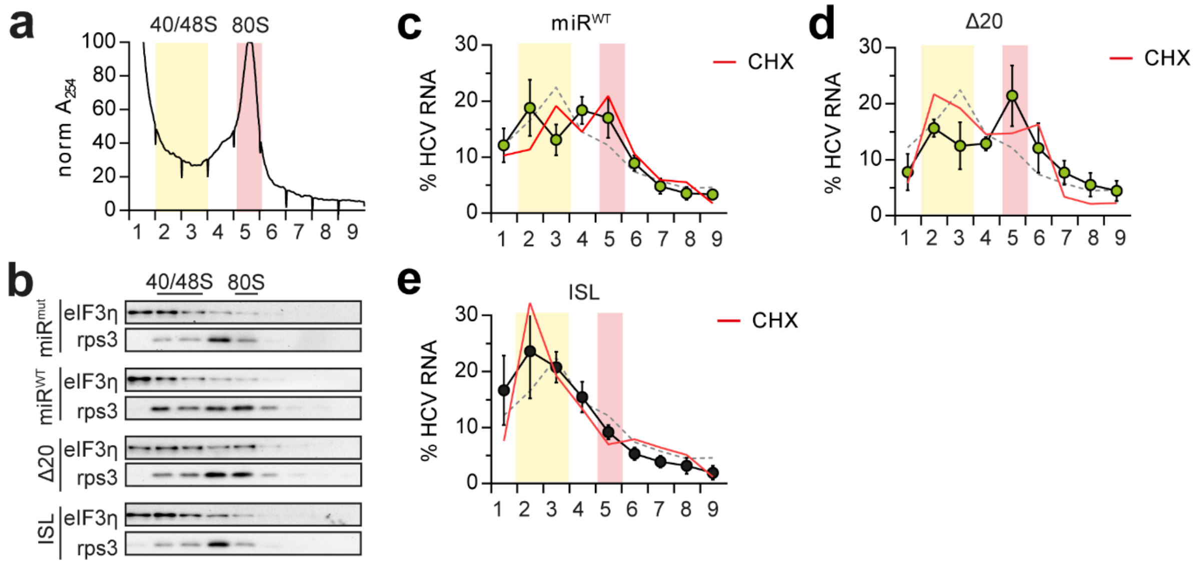

Supplementary Figure 10 – Supporting data from sucrose gradient fractionation. (a) Representative absorbance (A_{254}) profile of a fractionation experiment (HCV+miR^{mut}). Fractions containing the 40/48S (yellow) and 80S (red) complexes are indicated. **(b)** Western blots against eIF3η and rps3 as markers for pre 80S complexes and small ribosomal subunit, respectively, corresponding to the fractions shown in Fig 6c-f. **(c-e)** Translation initiation efficiency of Luc-SG in presence of miR^{WT} (c) or DI mutants Δ20 (d) and ISL (e) in absence or presence of 5µg/µl CHX. The control with miR^{mut} is shown as dashed line for comparison. Mean values (±SD), n=3 or n=1 for the CHX condition. 40/48S and 80S containing fractions are highlighted by yellow or orange boxes, respectively.

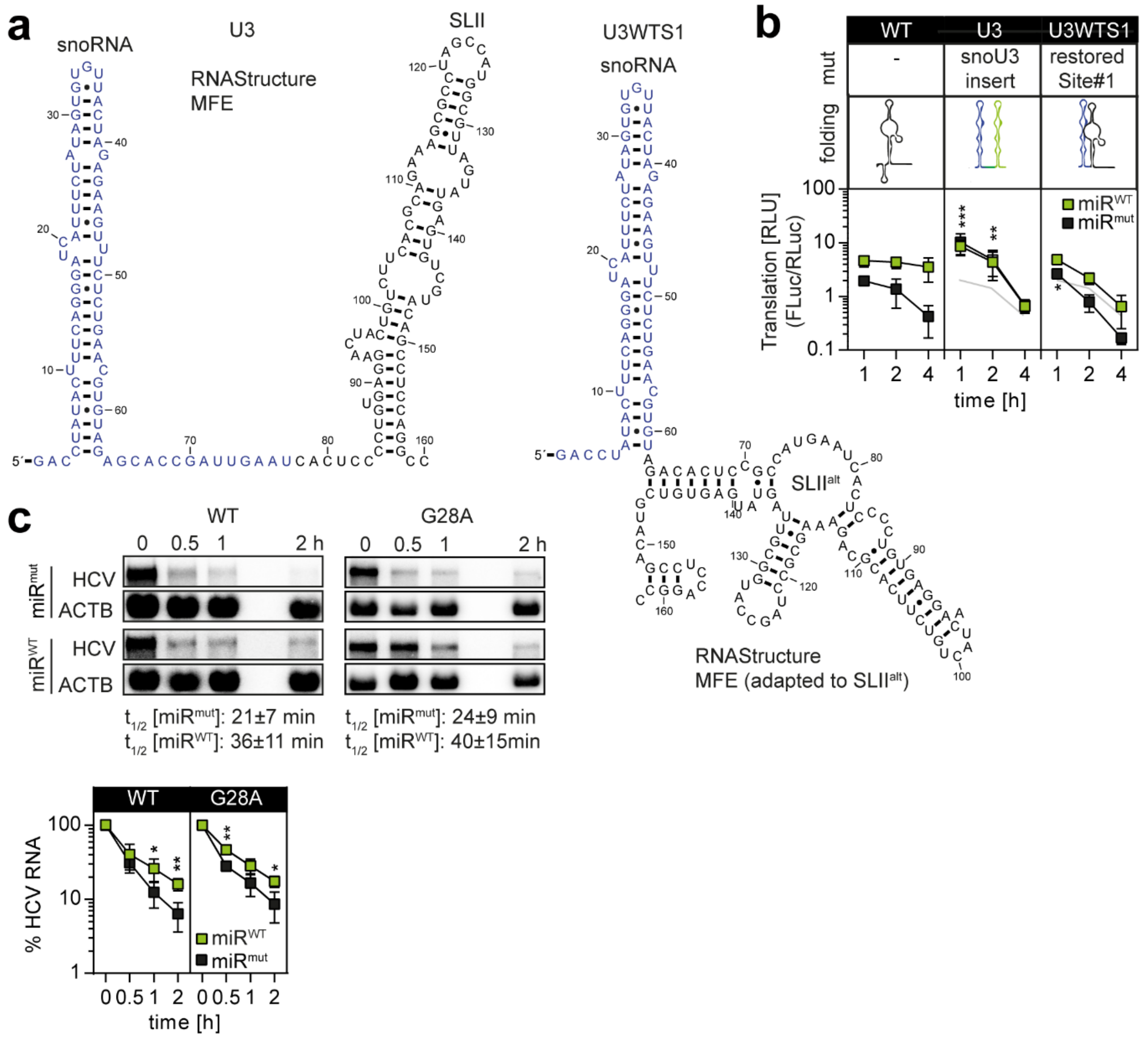

Supplementary Figure 11 – G28A and U3 mutants. (a) Representation of the 2D structure of U3 and the structural changes after reintroduction of miR-binding site 1 (U3WTS1). The primary prediction yielded a slightly different stem-loop at the position of SLII^{alt}, which also appeared in the WT 5' UTR sequence, without the implementation of SHAPE data. Therefore, the formation of SLII^{alt} was inferred from the previous data. **(b)** Translation assay using the Luc-SG replicon harboring U3 and U3WTS mutants versus wild-type in presence of miR^{mut} or miR^{WT}. **(c)** Representative autoradiography image and quantification of short term northern blot of WT and G28A to assess RNA decay kinetics. Mean values (±SD), n=3, in technical duplicates. *RLU*: relative light units; *ΔGDD*: replication deficient mutant. *: P<=0.05, **: P<=0.01, ***: P<0.001. For translation experiments, statistical significance was determined for WT against the mutant in presence of miR^{mut}, for northern blots, miR^{WT} against miR^{mut}.

Original northern blot from figure 2

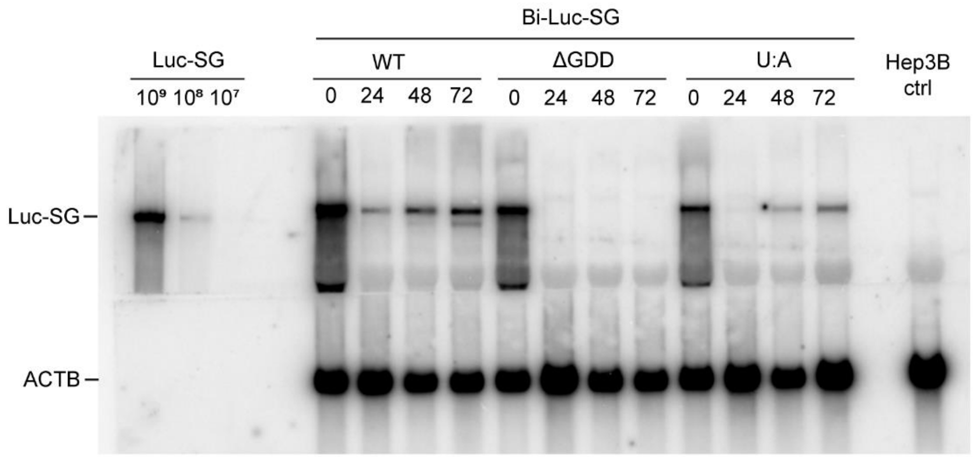

Original agaose gels from figure 6

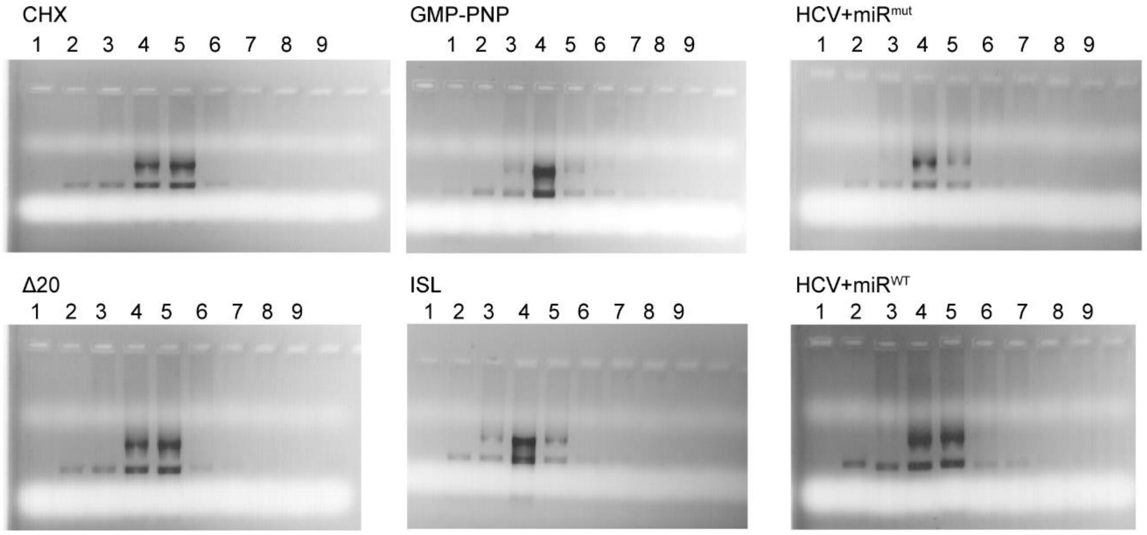

Supplementary Figure 12 – Uncropped northern blot and agarose gels from Figures 2 and 6

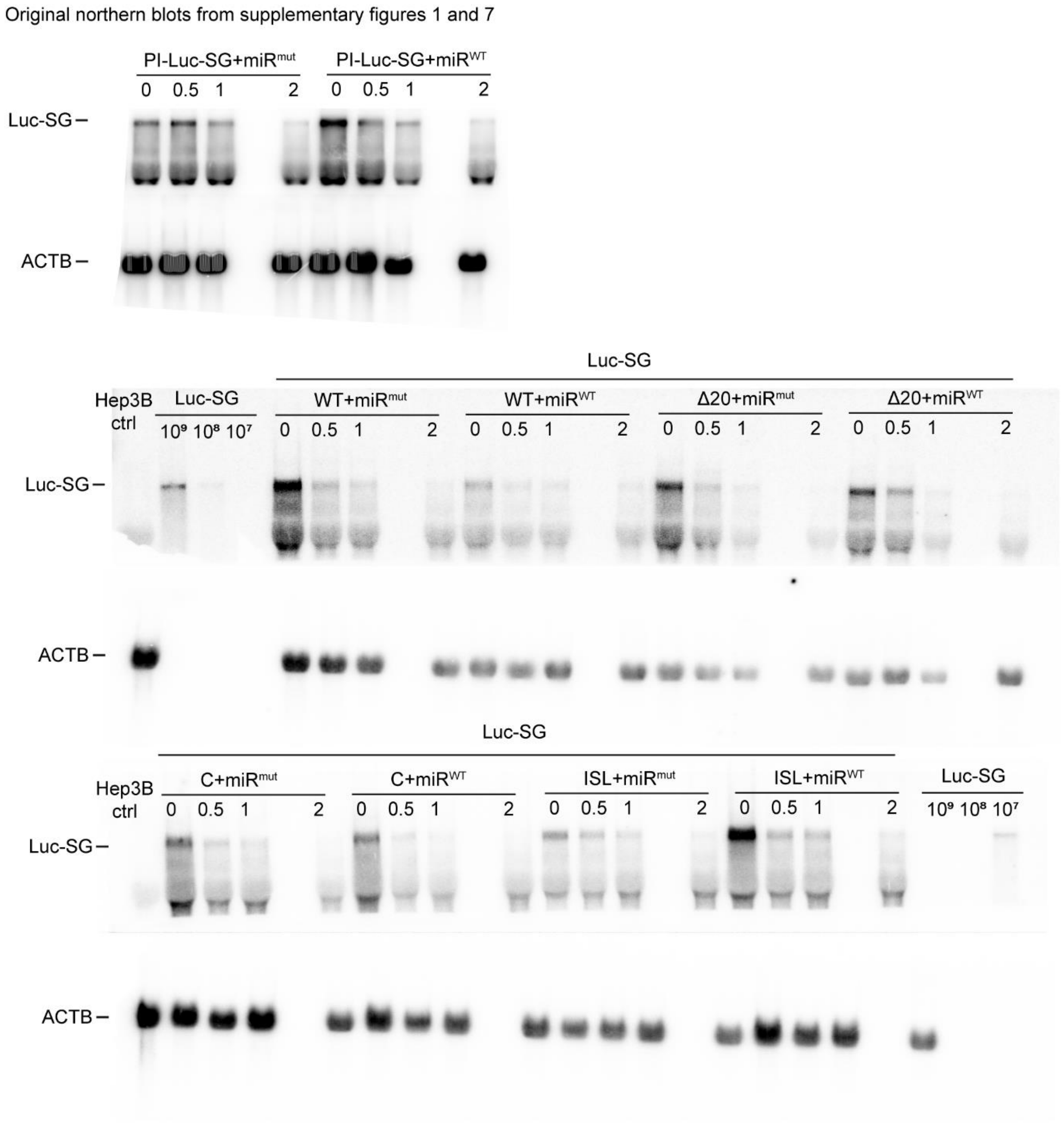

Supplementary Figure 13 – Uncropped northern blots from Supplementary Figures 1 and 7

Original western blots from supplementary figure 10

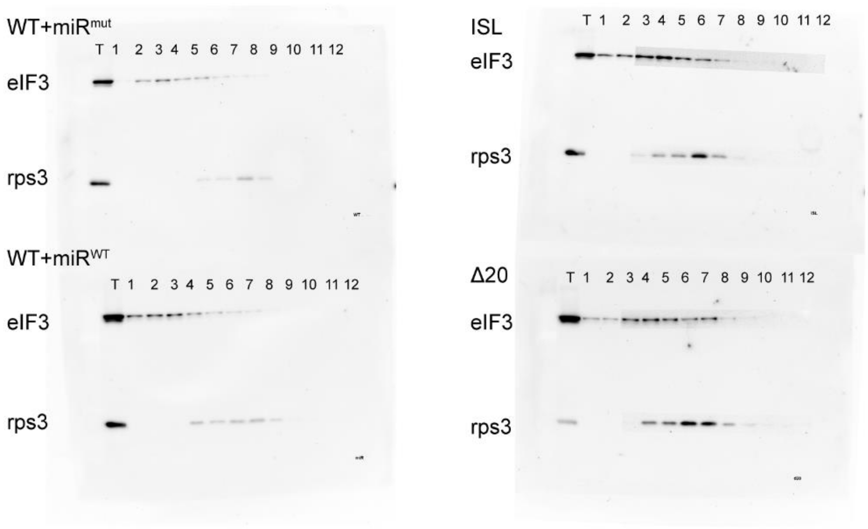

Original northern blots from supplementary figure 11

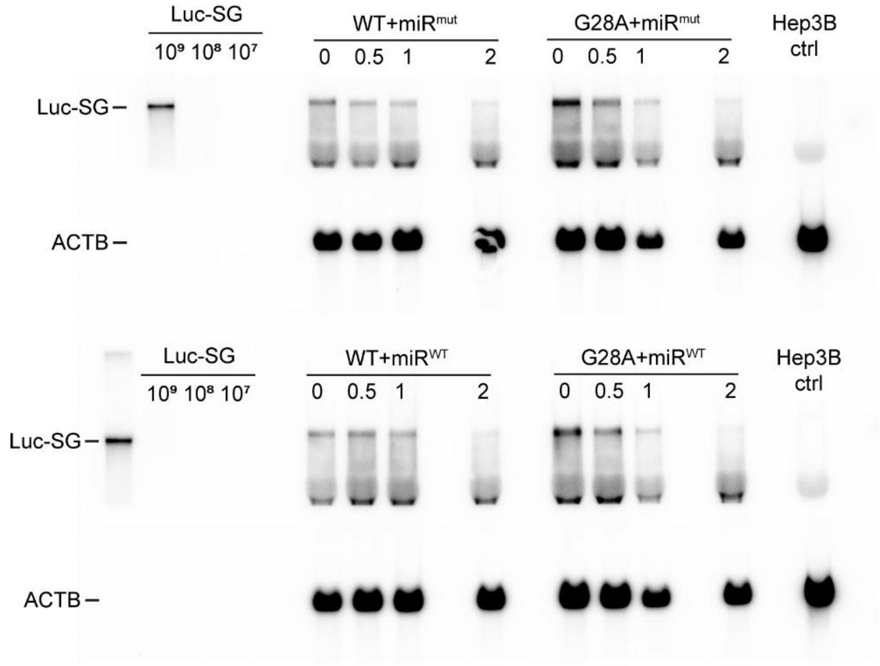

Supplementary Figure 14 – Uncropped western and northern blots from Supplementary Figures 10 and 11
